# *Portlandemys gracilis* n. sp., a New Coastal Marine Turtle from the Late Jurassic of Porrentruy (Switzerland) and a Reconsideration of Plesiochelyid Cranial Anatomy

**DOI:** 10.1371/journal.pone.0129193

**Published:** 2015-06-24

**Authors:** Jérémy Anquetin, Christian Püntener, Jean-Paul Billon-Bruyat

**Affiliations:** Section d'archéologie et paléontologie, Office de la culture, République et Canton du Jura, 2900 Porrentruy, Switzerland; University of Oxford, UNITED KINGDOM

## Abstract

**Background:**

Several groups of stem cryptodires became adapted to coastal marine environments as early as the Late Jurassic, 40 million years before the Pan-Chelonioidea. The Plesiochelyidae are a major component of this first radiation of crown-group turtles into marine habitats. They are abundant in many European localities, but their systematics is still greatly confused. Only three species are represented by cranial material: *Plesiochelys etalloni*, *Plesiochelys planiceps*, and *Portlandemys mcdowelli*.

**Methodology/Principal Findings:**

In the present study, we describe a cranium and a mandible from the Kimmeridgian of Porrentruy (Switzerland), which we refer to a new species, *Portlandemys gracilis* n. sp. This new taxon differs from *Portlandemys mcdowelli* in several aspects of the cranium and mandible, notably in being generally more gracile, but the two species share a narrow skull, a more acute angle between the labial ridges on the mandible, and a unique configuration of the anterodorsal part of the basicranium. The cranial anatomy of plesiochelyid turtles is discussed in details based primarily on these new specimens and new cranial material of *Plesiochelys etalloni* from Solothurn, Switzerland.

**Conclusions/Significance:**

Several characters (e.g., the contribution of the parietal to the foramen nervi trigemini, the configuration of the dorsum sellae and sella turcica, the presence of an infolding ridge on the posterior surface of the quadrate) appear as potential candidates to help elucidate plesiochelyid relationships. Some of these characters are included in a previously published phylogenetic dataset and help to stabilize the relationships of plesiochelyid turtles and closely related taxa. For the first time, our results suggest that plesiochelyids, *'Thalassemys' moseri*, and *Solnhofia parsonsi* (representing the Eurysternidae) form a clade at the base of Eucryptodira.

## Introduction

During the Kimmeridgian and Tithonian (Late Jurassic), most of Europe was covered by shallow epicontinental seas. Several groups of potentially related basal eucryptodire turtles (Plesiochelyidae Baur, 1888 [1], Thalassemydidae Zittel, 1889 [2], and Eurysternidae Dollo, 1886 [3]) thrived in those coastal marine environments. They represent the first radiation of eucryptodires into marine ecosystems and are likely not closely related to Pan-Chelonioidea Joyce et al., 2004 [4] that diversified later during the Early Cretaceous [5–13]. Eurysternids are usually interpreted as lagoonal forms because they are mostly found in lithographic limestones, such as those of Solnhofen, Cerin, and Canjuers [14]. In contrast, plesiochelyids and thalassemydids are regarded as forms inhabiting more open coastal environments. This interpretation has been partly confirmed by oxygen isotope composition of shell bones [15] and histology [16].

**Fig 1:**
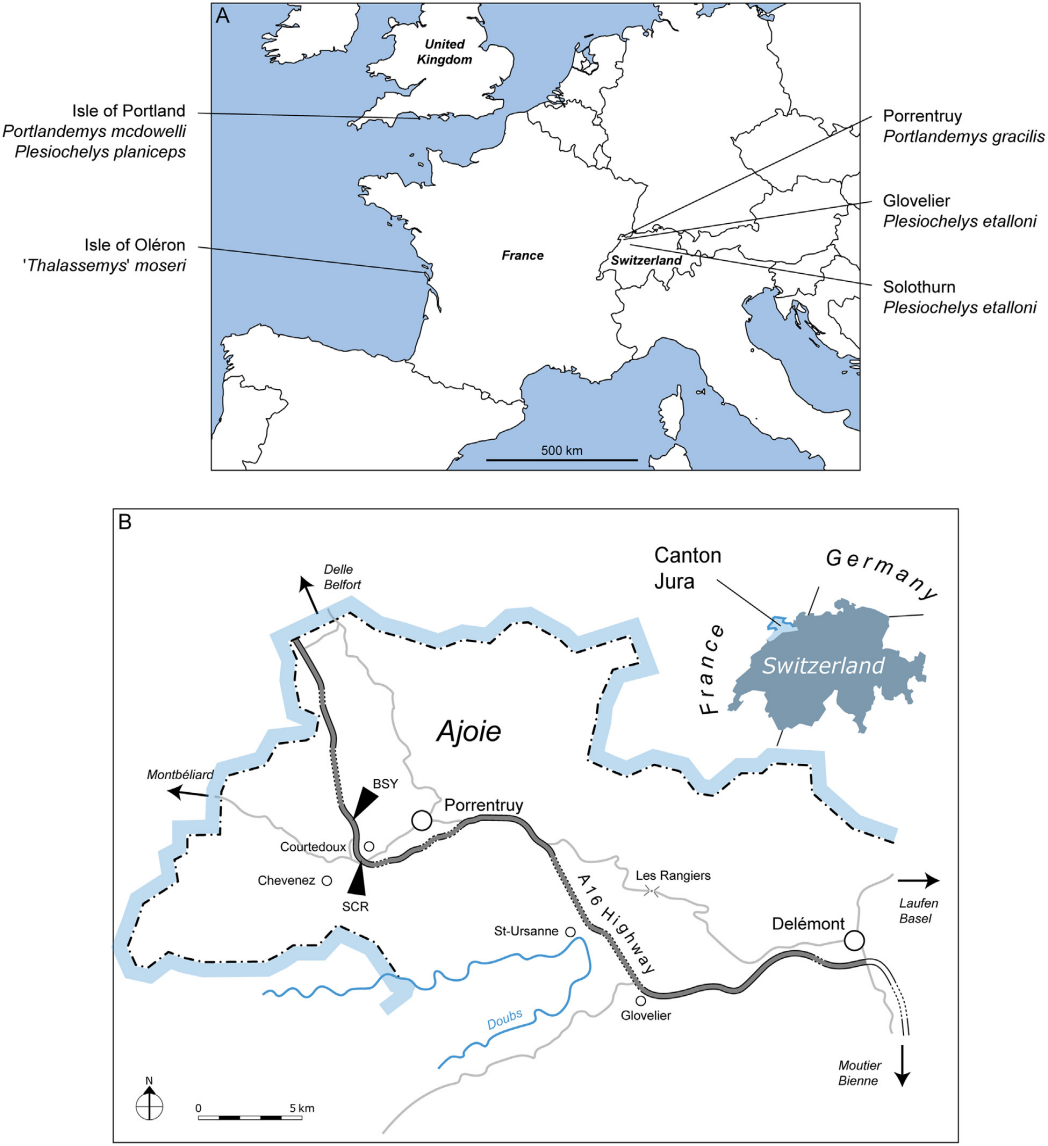
Localisation of the discussed specimens. (A) Map of Europe showing the localities of the specimens discussed in the text; (B) Map of the Ajoie region, Canton of Jura, Switzerland. The localities Bois de Sylleux (BSY) and Sur Combe Ronde (SCR) are indicated on the track of the A16 Transjurane highway (gray) in B.

Plesiochelyid systematics is a complex subject. Numerous species have been described during the 19th century based on more or less complete shell material from various European localities. This abundant material was given very little attention during the 20th century and needs to be revised in a global context [17]. In contrast, cranial material is much less common for plesiochelyids, but was given more attention during the past 40 years [18–22]. Plesiochelyid skulls are known from the Solothurn Turtle Limestone (Solothurn, Canton of Solothurn, Switzerland; Kimmeridgian; Fig 1) since the early 1820's [23–25], but they have been prepared and studied in details only recently [19]. The four skulls from Solothurn (NMS 8738, NMS 8739, NMS 8740, and NMS 9145) and a fifth skull (NMB 435) found associated with a shell in Glovelier (Canton of Jura, Switzerland; Kimmeridgian; Fig 1) were all referred to *Plesiochelys etalloni* (Pictet and Humbert, 1857 [26]) by Gaffney [19]. Parsons and Williams [18] described three skulls (NHMUK R2914, NHMUK R3163, and NHMUK R3164) from the Portland Beds of the Isle of Portland (Dorset, UK; Tithonian; Fig 1), which they identified as belonging to a single species. This species was subsequently named *Portlandemys mcdowelli* Gaffney, 1975a [19]. Finally, another skull from the Portland Beds (Tithonian) of the Isle of Portland (OUMNH J.1582), initially named *Chelone planiceps* Owen, 1842 [27], was later referred to the genus *Plesiochelys* Rütimeyer, 1873 [24] [19]. Therefore, nine plesiochelyid skulls representing three different taxa were described in the literature prior to the present study.

Between 2000 and 2011, controlled excavations along the future course of the A16 Transjurane highway in the vicinity of Porrentruy (Canton of Jura, NW Switzerland; Fig 1) led to the discovery of several vertebrate-bearing stratigraphical layers dated from the Kimmeridgian. Plesiochelyids, thalassemydids, and eurysternids have been found in great number during these excavations [28–31]. In the present paper, we describe a new plesiochelyid from the late Kimmeridgian Lower *Virgula* Marls (Chevenez Member, Reuchenette Formation). *Portlandemys gracilis* n. sp. is represented by a partial, isolated cranium (basicranium and skull roof). An isolated mandible from the same stratigraphical level is also tentatively referred to this new species. In order to understand the relationships of this new species, several aspects of the cranial anatomy of plesiochelyid turtles needed to be revised. In this, we were aided by the study of new cranial material of *Plesiochelys etalloni* from Solothurn, which allowed us to extend comparisons between *Portlandemys gracilis* and other known plesiochelyids. Based on all these new specimens, we discuss the cranial anatomy of plesiochelyid turtles, notably characters that could potentially help to resolve their uncertain relationships. In an attempt to evaluate their impact on plesiochelyid relationships, the most pertinent of these characters are included in a previously published phylogenetic dataset.

## Material and Methods

### Institutional abbreviations

AMNH, American Museum of Natural History, New York, USA; MAJ, Musée d'archéologie du Jura, Lons-le-Saunier, France; MJSN, JURASSICA Museum (formerly Musée jurassien des sciences naturelles), Porrentruy, Switzerland; NHMUK, Natural History Museum, London, UK; NMB, Naturhistorisches Museum Basel, Switzerland; NMS, Naturmuseum Solothurn, Switzerland; OUMNH, Oxford University Museum of Natural History, Oxford, UK; PIMUZ, Paläontologisches Institut und Museum, Universität Zürich, Switzerland; TM, Teyler Museum, Haarlem, Netherlands.

### Abbreviations of localities

BAN, Banné (Porrentruy, Switzerland); BSY, Bois de Sylleux (Courtedoux, Switzerland); SCR, Sur Combe Ronde (Courtedoux, Switzerland).

### Material

Four new specimens are described herein. Specimens MJSN BSY009-708 (a partial cranium) and MJSN SCR011-441 (a complete mandible) are from the late Kimmeridgian of Porrentruy, Switzerland. They are identified herein as a new plesiochelyid turtle, *Portlandemys gracilis* n. sp. The remaining two specimens, NMS 40870 (a sub-complete cranium) and NMS 40871 (a partial basicranium), are from the late Kimmeridgian of Solothurn, Switzerland. They are referred to *Plesiochelys etalloni* and used as comparative material for the description of the new taxon *Portlandemys gracilis* and as a starting point for the discussion on plesiochelyid cranial anatomy.

### Geological setting

Solothurn (Canton of Solothurn, Switzerland) is the richest known European locality for Late Jurassic turtles. More than 200 shells, five skulls, and thousands of disarticulated remains were collected from the 13 producing quarries between the beginning of the 19th century and the early 1920's [32,33]. The diversity of this turtle assemblage is also remarkable with eight species present [17,24,25]. The Solothurn Turtle Limestone is the uppermost member of the Reuchenette Formation and was dated from the Autissiodorensis ammonite zone (late Kimmeridgian). Most of the turtles were collected from the Rätschenbank, a highly fossiliferous layer of biomicritic limestone [32]. The Zetter quarry in St Niklaus, one of the original quarries, was briefly reopened from 1986 to 1989, which allowed the NMS and the Geological Institute of the University of Bern to led several excavation campaigns in order to investigate the depositional environment and paleoecology of the Rätschenbank [32,34]. Numerous turtle remains were collected during these excavation campaigns, but this material is still largely unstudied. For example, a complete, but still undescribed mandible of *Plesiochelys etalloni* was figured by Meyer ([35], pl. 1, Fig 7). NMS 40870 (a sub-complete cranium) and NMS 40871 (a partial braincase) are part of this material.

From 2000 to 2011, the PAL A16 team ("Section d'archéologie et paléontologie") led controlled excavations prior to the construction of the A16 Transjurane highway throughout the Jura Mountains (Canton of Jura, NW Switzerland; Fig 1). Excavations south of Porrentruy revealed the existence of several fossiliferous calcareous and marly horizons within the Reuchenette Formation [36]. The Lower *Virgula* Marls (Chevenez Member, Reuchenette Formation [37]) are by far the most productive horizon. A rich and diverse coastal marine assemblage was collected from this layer, including invertebrates (bivalves, gastropods, cephalopods, crustaceans, and echinoids), vertebrates (chondrichthyans, osteichthyans, turtles, crocodilians, and pterosaurs), and wood remains [28,29,38]. The Lower *Virgula* Marls are dated from the Eudoxus zone (late Kimmeridgian) [37]. These deposits are therefore slightly older than the Solothurn Turtle Limestone. The Lower *Virgula* Marls were excavated in several localities close to the small town of Courtedoux, southwest of Porrentruy. The cranium MJSN BSY009-708 was collected in Bois de Sylleux (BSY) in 2009. The mandible MJSN SCR011-441 was collected in Sur Combe Ronde (SCR) in 2011 (Fig 1).

### Nomenclatural acts

The electronic edition of this article conforms to the requirements of the amended International Code of Zoological Nomenclature, and hence the new names contained herein are available under that Code from the electronic edition of this article. This published work and the nomenclatural acts it contains have been registered in ZooBank, the online registration system for the ICZN. The ZooBank LSIDs (Life Science Identifiers) can be resolved and the associated information viewed through any standard web browser by appending the LSID to the prefix "http://zoobank.org/". The LSID for this publication is: urn:lsid:zoobank.org:pub:01BC7E0A-28CB-4191-8CDF-F6BDB962BEB6. The electronic edition of this work was published in a journal with an ISSN, and has been archived and is available from the following digital repositories: PubMed Central and LOCKSS.

### Anatomical comparisons

The following species and specimens have been used for comparative purposes: *Plesiochelys etalloni* (NMB 435, NMS 8738, NMS 8739, NMS 8740, and NMS 9145), *Plesiochelys planiceps* (OUMNH J.1582), *Portlandemys mcdowelli* (NHMUK R2914 and NHMUK R3164), and PIMUZ A/III 514 a specimen from the Tithonian of the Isle of Oléron (Department of Charente-Maritime, France) referred to *Thalassemys moseri* Bräm, 1965 [25] by Rieppel [39]. All of these specimens have been studied firsthand and original descriptions have been scrutinized as well [18–20,39]. Among the aforementioned taxa, the first three are traditionally referred to the Plesiochelyidae. The relationships of '*Thalassemys*' *moseri* sensu [39] are still confused [17]. The present study follows the anatomical nomenclature established by Gaffney [40,41], as updated by Rabi et al. [13] for the nomenclature of foramina and canals associated with the passage for the internal carotid artery and its subsidiaries.

### Phylogenetic methods

The parsimony analysis aims to evaluate the impact of new cranial characters on the relationships of plesiochelyids. It is based on a previously published character-taxon matrix [10], with the inclusion of five new characters (see Phylogenetic Analysis) and three additional plesiochelyid taxa (*Portlandemys gracilis*, *Plesiochelys planiceps*, and *Tropidemys langii* Rütimeyer, 1873 [24]). *Portlandemys gracilis* was scored based on the material described herein. *Plesiochelys planiceps* was scored based on firsthand observation of the holotype specimen (OUMNH J.1582). *Tropidemys langii* was scored following Püntener et al. [30]. The scoring of several key taxa was also altered based either on recently published literature or personal observations (see S1 File). The modified matrix consists of 141 characters scored for 71 taxa (S1 and S2 Files). It is also available on MorphoBank [42] as project P1166 (http://morphobank.org/permalink/?P1166).

The dataset was analysed using TNT v. 1.1 [43] with all of the characters equally weighted. Branches were set to collapse if they lack unambiguous support (rule 1 of [44]). As in the original phylogenetic analysis [10], the following 15 characters were ordered (note that the numbering of characters in TNT starts at 0, so that character numbers in MorphoBank would be n+1): 6 (Prefrontal A), 27 (Vomer A), 33 (Quadrate C), 35 (Quadrate E), 58 (Stapedial Artery B), 64 (Carapace A), 65 (Carapace B), 69 (Peripheral A), 72 (Costal B), 75 (Supramarginal A), 89 (Mesoplastron A), 102 (Abdominal A), 124 (Cleithrum A), 137 (Manus B), and 138 (Manus C). The hypothetical ancestor (taxon 0 in TNT) was set as outgroup. A heuristic search of 1000 replicates followed by TBR branch swapping keeping 10 trees per replicate was conducted. The best trees obtained at the end of this initial search were subjected to a final round of TBR branch swapping. Bremer support values were calculated using an iterative procedure where successively longer suboptimal trees are computed and compared to the strict consensus tree (see S1 File). A second analysis was run with a molecular scaffold constraining the relationships among living cryptodires following the results of Crawford et al. [45]. The objective was to test the impact of such a constraint on plesiochelyid relationships (see Results).

### 3D models

In order to facilitate and enhance future comparisons, we have built 3D models of the specimens described herein. These models were computed with the photogrammetry software Agisoft Photoscan 1.0.4 Standard Edition using sets of photographs of the specimens. We followed the procedures described recently by Mallison and Wings [46]. These models are provided herein as 3D PDFs (reduced resolution; to be opened with Adobe Acrobat): NMS 40870 (S3 File), NMS 40871 (S4 File), MJSN BSY009-708 (S5 File), and MJSN SCR011-441 (S6 File). Scaled and textured high-resolution meshes in PLY format are also available freely on figshare (http://figshare.com/authors/J_r_my_Anquetin/651097).

## Systematic Paleontology

Testudines Batsch, 1788 [47]

Eucryptodira Gaffney, 1975c [48]

Plesiochelyidae Baur, 1888 [1]

### Remarks

For the purpose of the present study, Plesiochelyidae is used according to its traditional acceptance [14,19,49] and includes *Plesiochelys etalloni*, *Plesiochelys planiceps*, *Portlandemys mcdowelli*, *Portlandemys gracilis*, as well as several shell-based taxa such as *Tropidemys langii*, *Craspedochelys picteti*, and *Craspedochelys jaccardi* (see [17,30]). The Plesiochelyidae, Thalassemydidae, and Eurysternidae are currently being revised as a whole, which is why we will refrain from discussing their definitions further herein.

*Plesiochelys* Rütimeyer, 1873 [24]

### Type species

*Plesiochelys solodurensis* Rütimeyer, 1873 [24]

### Revised diagnosis

Type genus of the Plesiochelyidae. Differing from *Portlandemys* in: skull proportionally shorter (lower length/width ratio); more obtuse angle between labial ridges of maxilla and dentary; dorsum sellae not overhanging sella turcica, but foramina anterius canalis carotici cerebralis located only slightly anterior to dorsum sellae. See Anquetin et al. [17] for shell characters.

### Included valid species

*Plesiochelys etalloni* (Pictet and Humbert, 1857 [26]); *Plesiochelys planiceps* (Owen, 1842 [27]).

*Plesiochelys etalloni* (Pictet and Humbert, 1857 [26])

(Figs 2–4)

**Fig 2:**
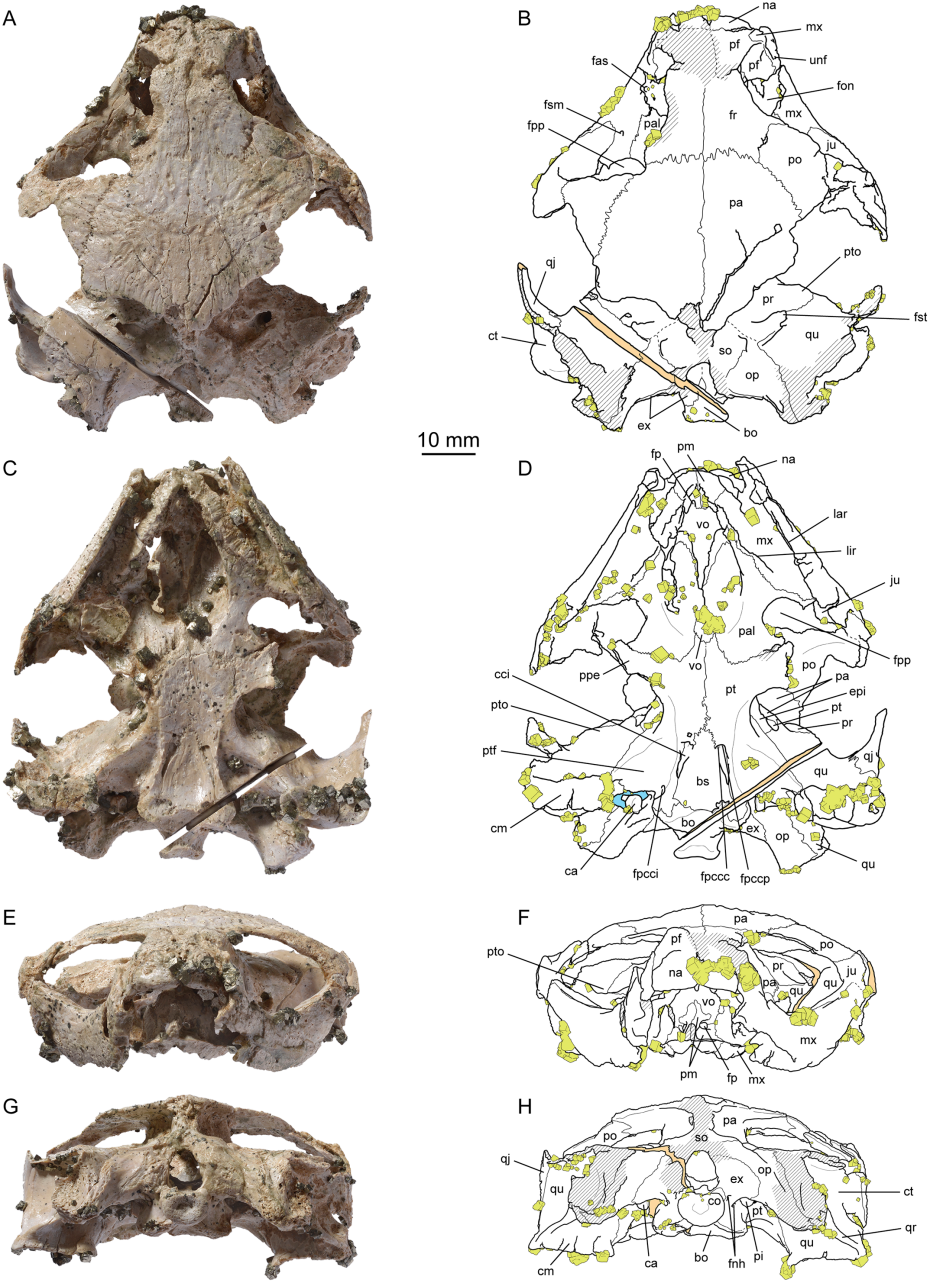
*Plesiochelys etalloni*, cranium, NMS 40870. (A) photograph in dorsal view; (B) interpretative drawing in dorsal view; (C) photograph in ventral view; (D) interpretative drawing in ventral view; (E) photograph in anterior view; (F) interpretative drawing in anterior view; (G) photograph in posterior view; (H) interpretative drawing in posterior view. Abbreviations: bo, basioccipital; bs, basisphenoid; ca, columella auris; cci, canalis caroticus internus; cm, condylus mandibularis; co, condylus occipitalis; ct, cavum tympani; epi, epipterygoid; ex, exoccipital; fas, foramen alveolare superius; fnh, foramen nervi hypoglossi; fon, foramen orbito-nasale; fp, foramen praepalatinum; fpccc, foramen posterius canalis carotici cerebralis; fpcci, foramen posterius canalis carotici interni; fpccp, foramen posterius canalis carotici palatinum; fpp, foramen palatinum posterius; fr, frontal; fsm, foramen supramaxillare; fst, foramen stapedio-temporale; ju, jugal; lar, labial ridge; lir, lingual ridge; mx, maxilla; na, nasal; op, opisthotic; pa, parietal; pal, palatine; pf, prefrontal; pi, processus interfenestralis; pm, premaxilla; po, postorbital; ppe, processus pterygoideus externus; pr, prootic; pt, pterygoid; ptf, pterygoid fossa; pto, processus trochlearis oticum; qj, quadratojugal; qr, quadrate ridge; qu, quadrate; so, supraoccipital; unf, unnamed foramen; vo, vomer. Hatchings represent damaged areas. The sectioned surface is represented in orange. Pyrite crystals are in yellow. Blue in (D) represent consolident infilling.

**Fig 3:**
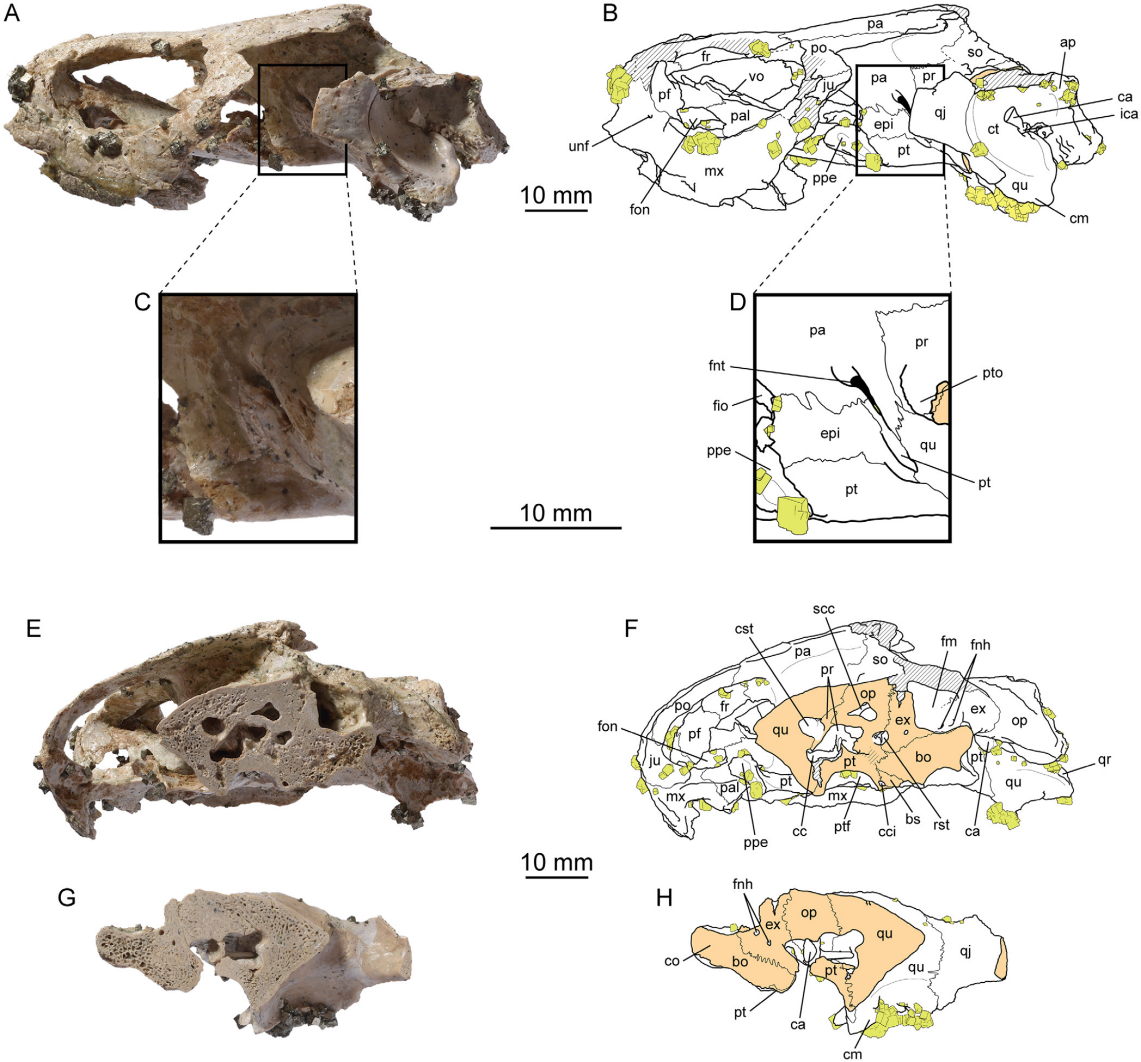
*Plesiochelys etalloni*, cranium, NMS 40870. (A) photograph in left lateral view; (B) interpretative drawing in left lateral view; (C) photograph of the left ethmoid region; (D) interpretative drawing of the left ethmoid region; (E) photograph of the sectioned left otic chamber in posterolateral view; (F) interpretative drawing of the sectioned left otic chamber in posterolateral view; (G) photograph of the sectioned left otic chamber in anteromedial view; (H) interpretative drawing of the sectioned left otic chamber in anteromedial view. Abbreviations: ap, antrum postoticum; bo, basioccipital; bs, basisphenoid; ca, columella auris; cc, canalis cavernosus; cci, canalis caroticus internus; cm, condylus mandibularis; co, condylus ocipitalis; cst, canalis stapedio-temporalis; ct, cavum tympani; epi, epipterygoid; ex, exoccipital; fio, foramen interorbitale; fm, foramen magnum; fnh, foramen nervi hypoglossi; fnt, foramen nervi trigemini; fon, foramen orbito-nasale; fr, frontal; ica, incisura columellae auris; ju, jugal; mx, maxilla; op, opisthotic; pa, parietal; pal, palatine; pf, prefrontal; po, postorbital; ppe, processus pterygoideus externus; pr, prootic; pt, pterygoid; ptf, pterygoid fossa; pto, processus trochlearis oticum; qj, quadratojugal; qr, quadrate ridge; qu, quadrate; rst, recessus scalae tympani; scc, semicircular canals; so, supraoccipital; unf, unnamed foramen; vo, vomer. Hatchings represent damaged areas. The sectioned surface is represented in orange. Pyrite crystals are in yellow.

**Fig 4:**
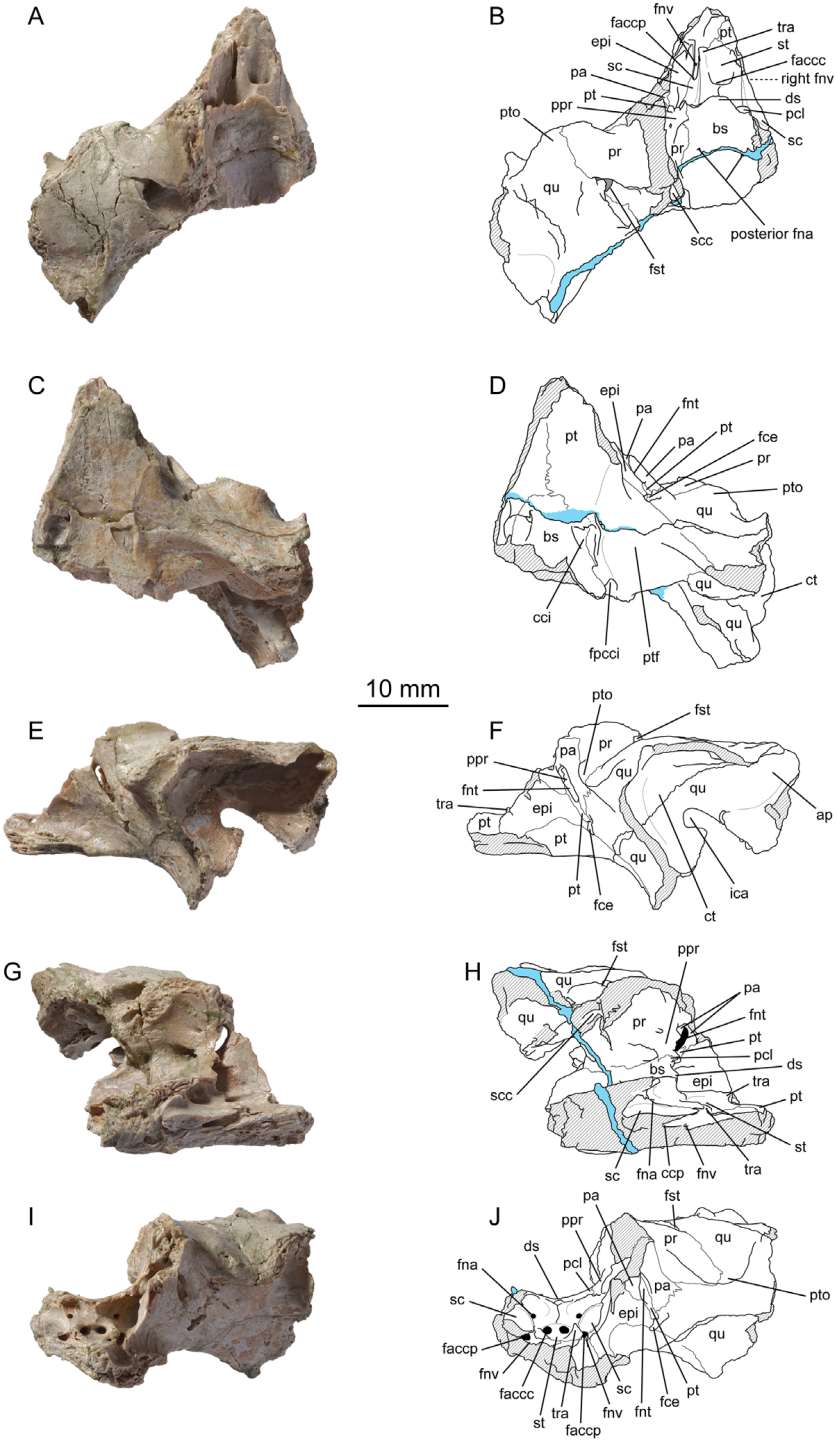
*Plesiochelys etalloni*, basicranium, NMS 40871. (A) photograph in dorsal view; (B) interpretative drawing in dorsal view; (C) photograph in ventral view; (D) interpretative drawing in ventral view; (E) photograph in left lateral view; (F) interpretative drawing in left lateral view; (G) photograph in medial view; (H) interpretative drawing in medial view; (I) photograph in anterior view; (J) interpretative drawing in anterior view. Abbreviations: ap, antrum postoticum; bs, basisphenoid; cci, canalis caroticus internus; ccp, canalis caroticus palatinum; ct, cavum tympani; ds, dorsum sellae; epi, epipterygoid; faccc, foramen anterius canalis carotici cerebralis; faccp, foramen anterius canalis carotici palatinum; fce, fossa cartilaginis epipterygoidei; fna, foramen nervi abducentis; fnt, foramen nervi trigemini; fnv, foramen nervi vidiani; fpcci, foramen posterius canalis carotici interni; fst, foramen stapedio-temporale; ica, incisura columellae auris; pa, parietal; pcl, processus clinoideus; ppr, pila prootica; pr, prootic; pt, pterygoid; ptf, pterygoid fossa; pto, processus trochlearis oticum; qu, quadrate; sc, sulcus cavernosus; scc, semicircular canals; st, sella turcica; tra, trabecula. Hatchings represent damaged areas. Blue represents a fissure filled with consolident.

### Holotype

MAJ 2005-11-1, a shell missing a large part of the carapace medially [26,50].

### Referred material

See Anquetin et al. [17]; NMS 40870, a sub-complete cranium with sectioned left otic chamber; NMS 40871, a fragment of basicranium consisting of the basisphenoid and partial left otic chamber.

### Type locality and horizon

"Forêt de Lect" near Moirans-en-Montagne, Jura, France. Kimmeridgian or early Tithonian, Late Jurassic [50].

### Other occurrences

Late Jurassic (Kimmeridgian) of Solothurn, Canton of Solothurn, and Glovelier, Canton of Jura, Switzerland.

### Revised diagnosis

Differing from *Plesiochelys planiceps* in: smaller skull size; complete ossification of pila prootica; rounded foramen palatinum posterius; parietal meeting quadrate posteroventral to foramen nervi trigemini; processus trochlearis oticum moderately developed; foramen posterius canalis carotici interni on ventral surface of pterygoid; superficial canalis caroticus internus; contribution of exoccipital to condylus occipitalis minor or absent; lower lingual ridge on maxilla; lingual ridges of the lower jaw curving anteriorly in the symphyseal area. See Anquetin et al. [50] for shell characters.

*Portlandemys* Gaffney, 1975a [19]

### Type species

*Portlandemys mcdowelli* Gaffney, 1975a [19]

### Revised diagnosis

Differing from *Plesiochelys* in: skull proportionally narrower (higher length/width ratio); more acute angle between labial ridges of maxilla and dentary; dorsum sellae not overhanging sella turcica and foramina anterius canalis carotici cerebralis located about halfway between dorsum sellae and tip of trabeculae.

### Included valid species

*Portlandemys mcdowelli* Gaffney, 1975a [19]; *Portlandemys gracilis* n. sp.

*Portlandemys gracilis* n. sp.

urn:lsid:zoobank.org:act:EEA3C356-8A08-4687-B182-BB2A650F48A2

(Figs 5–8)

**Fig 5:**
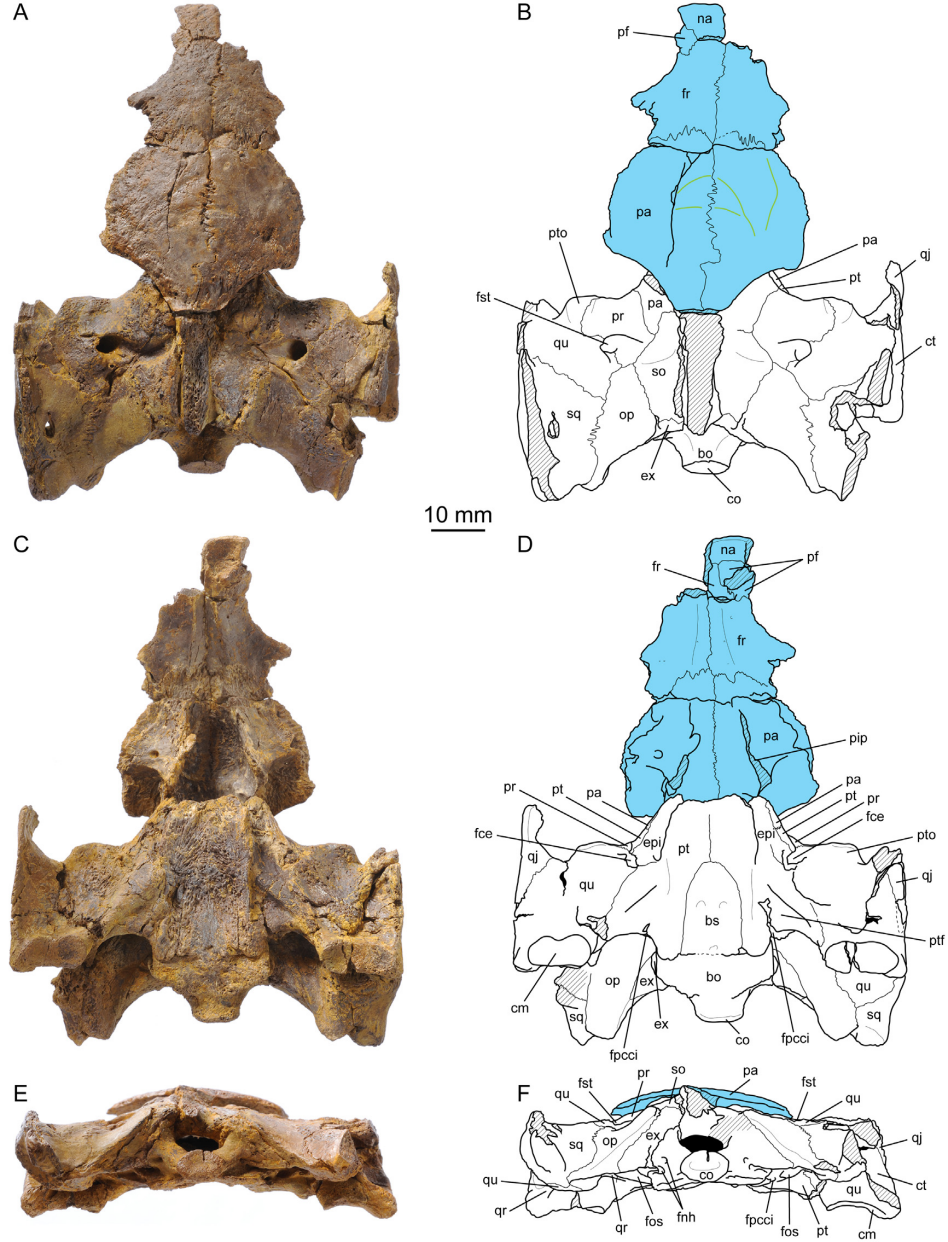
*Portlandemys gracilis* n. sp., cranium, MJSN BSY009-708 (holotype). (A) photograph in dorsal view; (B) interpretative drawing in dorsal view; (C) photograph in ventral view; (D) interpretative drawing in ventral view; (E) photograph in posterior view; (F) interpretative drawing in posterior view. Abbreviations: bo, basioccipital; bs, basisphenoid; cm, condylus mandibularis; co, condylus occipitalis; ct, cavum tympani; epi, epipterygoid; ex, exoccipital; fce, fossa cartilaginis epipterygoidei; fnh, foramen nervi hypoglossi; fos, fossa; fpcci, foramen posterius canalis carotici interni; fr, frontal; fst, foramen stapedio-temporale; na, nasal; op, opisthotic; pa, parietal; pf, prefrontal; pip, processus inferior parietalis; pr, prootic; pt, pterygoid; ptf, pterygoid fossa; pto, processus trochlearis oticum; qj, quadratojugal; qr, quadrate ridge; qu, quadrate; sq, squamosal; so, supraoccipital. Skull roof (now separated) is in blue. Hatchings represent damaged areas. Green lines on the skull roof represent scale sulci.

**Fig 6:**
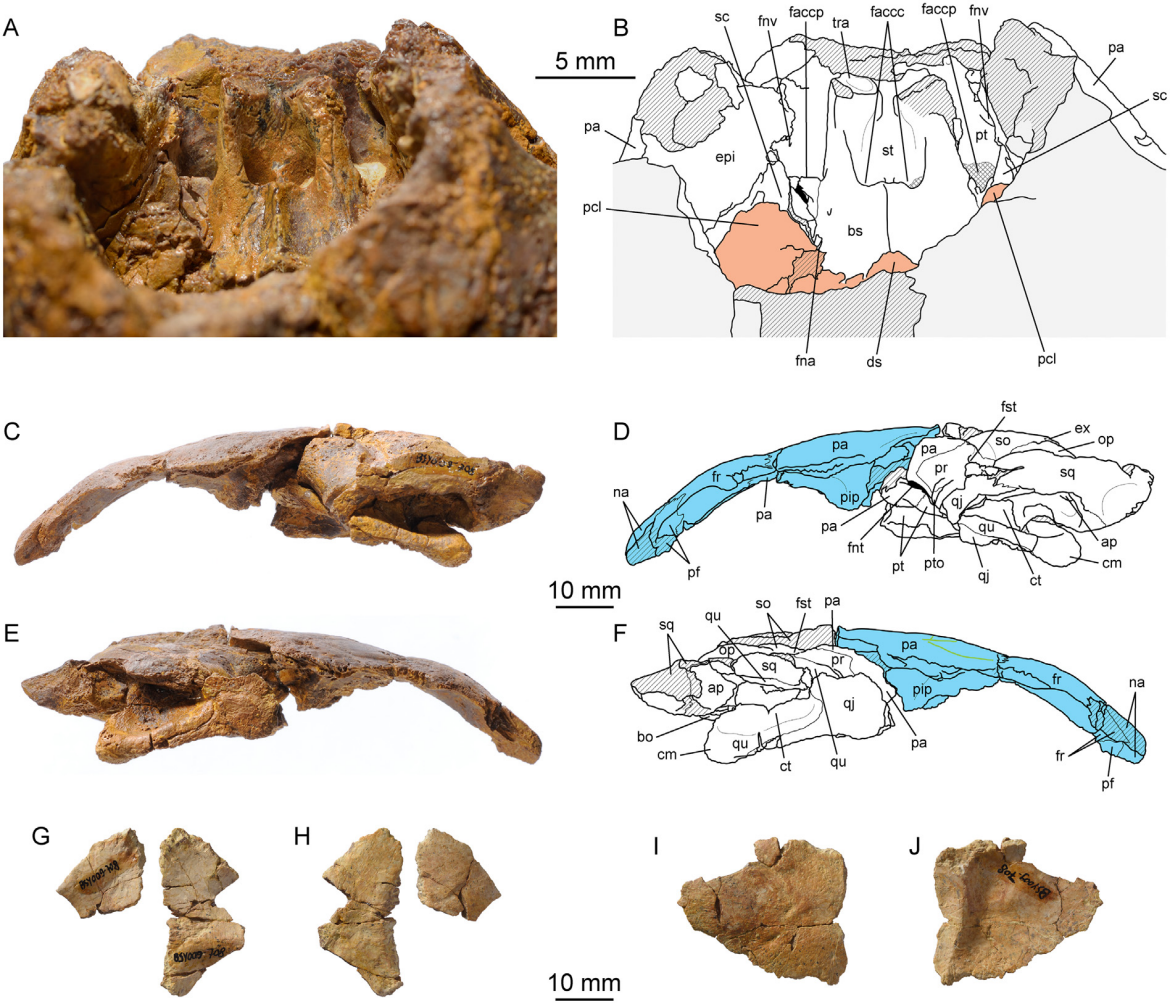
*Portlandemys gracilis* n. sp., cranium, MJSN BSY009-708 (holotype). (A) photograph of the area of the sella turcica in dorsal view; (B) interpretative drawing of the area of the sella turcica in dorsal view; (C) photograph in left lateral view; (D) interpretative drawing in left lateral view; (E) photograph in right lateral view; (F) interpretative drawing in right lateral view; (G) photograph of the palatines in dorsal view; (H) photograph of the palatines in ventral view; (I) photograph of the right postorbital in lateral view; (J) photograph of the right postorbital in medial view. Abbreviations: ap, antrum postoticum; bo, basioccipital; bs, basisphenoid; cm, condylus mandibularis; ct, cavum tympani; ds, dorsum sellae; epi, epipterygoid; ex, exoccipital; faccc, foramen anterius canalis carotici cerebralis; faccp, foramen anterius canalis carotici palatinum; fna, foramen nervi abducentis; fnt, foramen nervi trigemini; fnv, foramen nervi vidiani; fr, frontal; fst, foramen stapedio-temporale; na, nasal; op, opisthotic; pa, parietal; pcl, processus clinoideus; pf, prefrontal; pip, processus inferior parietalis; pr, prootic; pt, pterygoid; pto, processus trochlearis oticum; qj, quadratojugal; qu, quadrate; sc, sulcus cavernosus; so, supraoccipital; sq, squamosal; st, sella turcica; tra, trabecula. Orange in (B) represents the dorsum sellae and clinoid processes. Skull roof (now separated) is in blue. Hatchings represent damaged areas. Green lines on the skull roof represent scale sulci.

**Fig 7:**
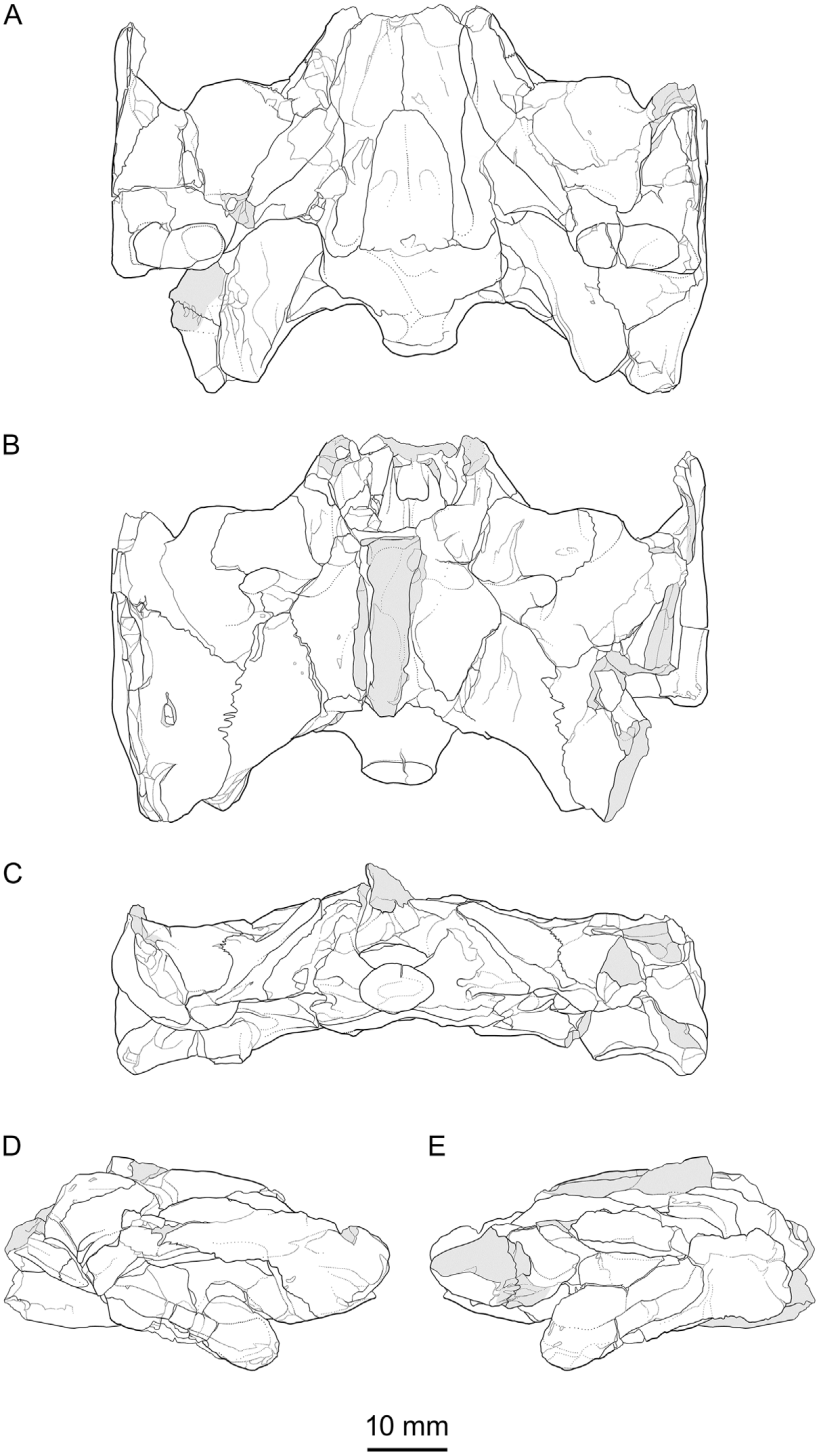
*Portlandemys gracilis* n. sp., cranium, MJSN BSY009-708 (holotype). High precision line drawings based on laser orthographic projection. (A) ventral view; (B) dorsal view; (C) posterior view; (D) left lateral view; (E) right lateral view. Original file at scale 1.

**Fig 8:**
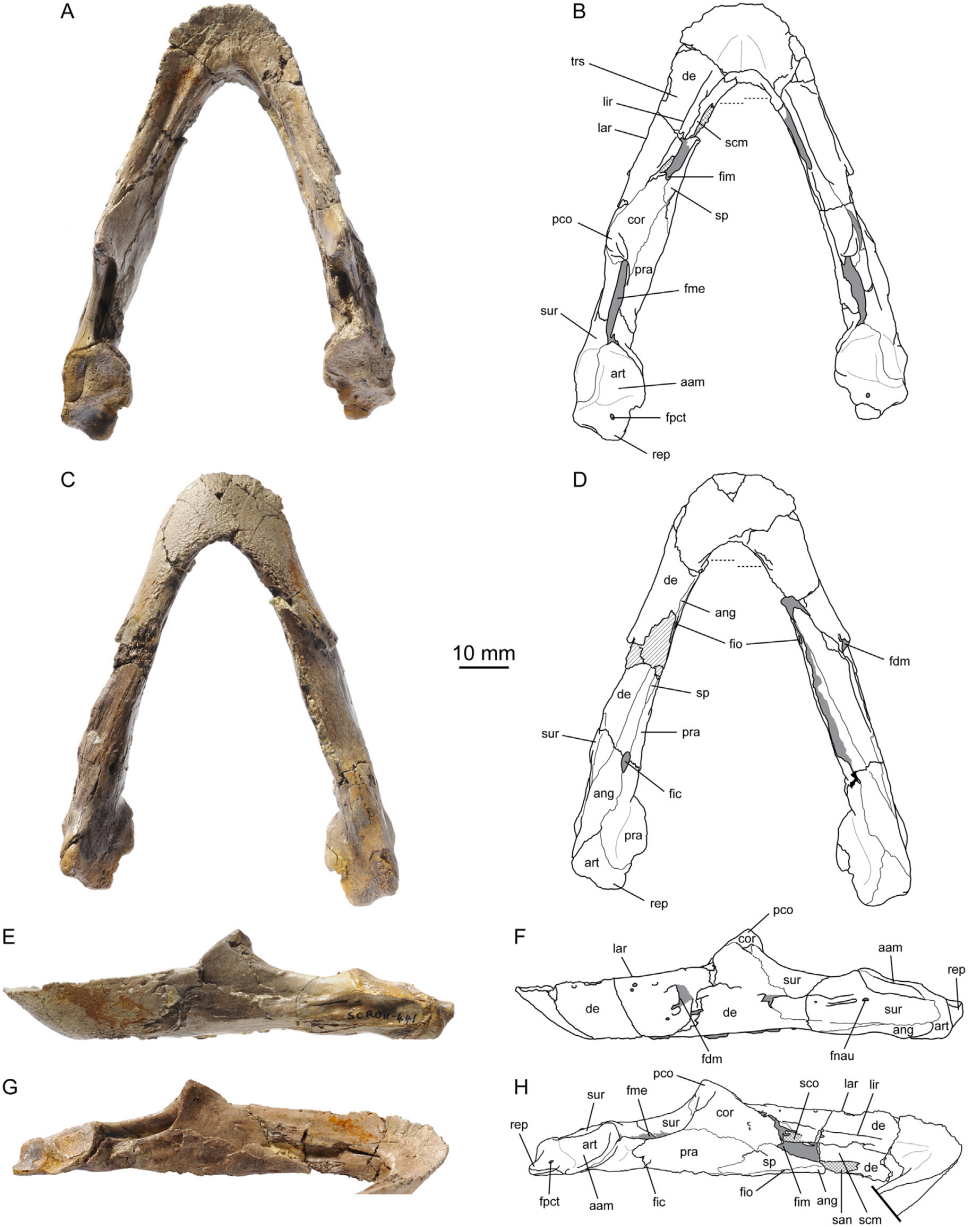
*Portlandemys gracilis* n. sp., mandible, MSJN SCR011-441. (A) photograph in dorsal view; (B) interpretative drawing in dorsal view; (C) photograph in ventral view; (D) interpretative drawing in ventral view; (E) photograph in left lateral view; (F) interpretative drawing in left lateral view; (G) photograph of the medial surface of the left ramus; (H) interpretative drawing of the medial surface of the left ramus. Abbreviations: aam, area articularis mandibularis; ang, angular; art, articular; cor, coronoid; de, dentary; fdm, foramen dentofaciale majus; fic, foramen intermandibularis caudalis; fim, foramen intermandibularis medius; fio, foramen intermandibularis oralis; fme, fossa meckelii; fnau, foramen nervi auriculotemporalis; fpct, foramen posterius chorda tympani; lar, labial ridge; lir, lingual ridge; pco, processus coronoideus; pra, prearticular; rep, retroarticular process; san, suture with angular; scm, sulcus cartilaginis meckelii; sco, suture with coronoid; sp, splenial; sur, surangular; trs, triturating surface. Matrix is in gray. Hatchings represent damaged areas. Cross-hatchings represent sutural surfaces. Dashed lines in (B) and (D) mark the anteriormost extension of the angular.

### Holotype

MJSN BSY009-708, a partial cranium consisting of the basicranium and the skull roof (Figs 5–7).

### Etymology

From the Latin *gracilis* for "slender". The name refers to the gracile morphology of this taxon compared to that of the type species, *Portlandemys mcdowelli*.

### Type locality and horizon

Bois de Sylleux, Courtedoux, near Porrentruy, Switzerland. Lower *Virgula* Marls, Chevenez Member, Reuchenette Formation, late Kimmeridgian, Late Jurassic [37].

### Referred specimen

MJSN SCR011-441, a complete mandible (Fig 8). This specimen is tentatively referred to this new species based on morphological correspondences between the mandible and the holotype (see below).

### Diagnosis

Differing from *Portlandemys mcdowelli* in: cranium more gracile (skull roof and basicranium less robustly ossified); reduced, narrow processus trochlearis oticum formed mostly by quadrate; broad contact between nasal and frontal on dorsal surface of skull roof preventing midline contact of prefrontals; pterygoid fossa not bordered posteriorly by ridge; foramen posterius canalis carotici interni located on posterior margin of pterygoid (barely visible in ventral view) further away from basisphenoid-pterygoid suture; raised pedestal on dorsal surface of pterygoid contacting processus interfenestralis of opisthotic; basisphenoid ogival in outline in ventral view; mandible more gracile; mandible with low profile in lateral view and broadly rounded symphyseal region; splenial triangular in shape with extended anterior part.

## Descriptions

### New information on the cranium of *Plesiochelys etalloni* (NMS 40870 and NMS 40871)

#### General description

NMS 40870 is an isolated, sub-complete cranium only missing parts of the temporal skull roof, the anterior part of the premaxillae, and the squamosals. This specimen is covered by numerous pyrite crystals (Figs 2 and 3; S3 File). On the field, the left otic chamber was sectioned from the foramen magnum to the processus trochlearis oticum with a circular saw. Apart from this incident, the preservation of this specimen is excellent. Taking into account the few millimeters of bone missing in the left otic chamber, the dimensions of the cranium are as follow: length = 78 mm; width = 69 mm (Table 1). These dimensions are very close to those of NMS 8739, which makes these two skulls the largest yet known for *Plesiochelys etalloni*. The skull length/width ratio of *Plesiochelys etalloni* is comparable to that of *Plesiochelys planiceps* and '*Thalassemys*' *moseri*, but is notably shorter than that of *Portlandemys gracilis* and *Portlandemys mcdowelli* (Table 1). NMS 40870 is the most complete skull of *Plesiochelys etalloni* known from Solothurn and can be directly compared with the smaller specimen from Glovelier (NMB 435). Interestingly, the outline of the cranium is different between the two specimens. In NMB 435, the widest part of the cranium is just anterior to the cavum tympani, then the width gradually decreases anteriorly resulting in a pointed outline in dorsal or ventral view. In NMS 40870, the cranium remains wide from the cavum tympani to the posterior border of the orbit, then decreases abruptly in width. As a result, the outline of the cranium appears stouter in dorsal or ventral view (Fig 2A–2D). However, it should be noted that the width of the skull just behind the orbits may have been slightly increased by the moderate downward deformation that affected the interorbital region of the skull roof. NMS 40871 consists of a fragment of basicranium with basisphenoid and partial left otic chamber (Fig 4; S4 File). This specimen is particularly interesting because the internal structures and ethmoid region are perfectly preserved. Unless otherwise stated, the following description is based primarily on NMS 40870.

**Table 1:** Length, width, and length/width ratio of selected plesiochelyid skulls.

	Total length from condylus occipitalis to tip of the snout (mm)	Length from pt-vo/pal suture to condylus occipitalis (mm)	Width at the level of the condyli mandibularis (mm)	Skull length/width ratio (only for the most complete specimens)
*Plesiochelys etalloni*				
NMB 435	55^a^	25.4^a^	54.6^b^	–
NMS 8738	–	34	53^b^	–
NMS 8739	76.5	40.4	–	–
NMS 8740	72^c^	31.7^c^	58.6^b^	–
NMS 9145	–	34.9	57.5	–
NMS 40870	78^d^	40.5^d^	69^d^	1.13
*Plesiochelys planiceps*				
OUMNH J.1582	82^c^	48	72^b^	1.14
*Portlandemys mcdowelli*				
NHMUK R2914	95.5	47.5	70^b^	1.36
NHMUK R3164	–	34	37^b^	–
*Portlandemys gracilis* n. sp.				
MJSN BSY009-708	92^c^	42^c^	73.4	1.25
'*Thalassemys*' *moseri*				
PIMZH A/III 514	48.5	26.1	43^b^	1.13

Abbreviations: pal, palatine; pt, pterygoid; vo, vomer.

^a^Condylus occipitalis and basioccipital missing (should be about 10 mm longer).

^b^Deduced from approximate half-width (specimen incomplete).

^c^Specimen incomplete.

^d^Left otic chamber sectioned (some bone missing).

#### Nasal

The nasal is preserved on both sides and forms the dorsal margin of the apertura narium externa (Fig 2A and 2B). Most of the dorsal surface of the left nasal is eroded, obscuring the contacts of this bone. On the right hand side, the dorsal surface of the nasal is better preserved. The bone is sub-quadrangular in outline, but the posterolateral corner is bevelled. The nasal contacts the other nasal medially and the maxilla laterally. Posteriorly, the nasal appears to contact only the prefrontal along a transverse suture (see Prefrontal). On the ventral surface, there is a medial contact between the nasal and the frontal as the latter extends anteriorly fully separating the prefrontals. The nasal slopes down strongly anteriorly and appears almost vertical in lateral view. The dorsal surface is slightly convex, whereas the ventral surface is strongly concave forming a dorsal recess in the roof of the fossa nasalis. This description is congruent with what is known in other specimens referred to *Plesiochelys etalloni* (NMB 435, NMS 8738, and NMS 8740).

#### Prefrontal

The dorsal part of each prefrontal is poorly preserved. It is likely that the prefrontals contacted one another in the midline for most of their length on the dorsal surface, but this contact is mostly obscured because the bone surface is eroded in this area (Fig 2A and 2B). Therefore, it would seem that there was no nasal-frontal contact on the dorsal surface of the skull roof. The dorsal part of the prefrontal contacts the nasal anteriorly, the maxilla anteroventrally, and the frontal posteriorly. The shape of the latter contact is uncertain due to poor preservation. On the ventral surface, an anterior medial process of the frontals fully separates the prefrontals. The dorsal plate of the prefrontal forms the anterodorsal corner of the orbit. The ventral, vertical plate of the prefrontal forms the anterior wall of the orbit, the lateral margin of the fissura ethmoidalis, and the anteromedial margin of the foramen orbito-nasale. The ventral plate of the prefrontal contacts the maxilla laterally, the vomer ventromedially and the palatine posteriorly. The description of this bone is consistent with what is known in other specimens referred to *Plesiochelys etalloni* (NMB 435, NMS 8738, NMS 8739, and NMS 8740).

#### Frontal

The frontal is better preserved on the right side of the specimen. On the left hand side, the posterodorsal orbital margin is damaged, making the orbit appear much larger and obliterating the frontal-postorbital suture (Fig 2A and 2B). The frontal forms the dorsal margin of the orbit. It contacts the other frontal medially, the prefrontal anteriorly, the postorbital posterolaterally, and the parietal posteriorly. As discussed above, the shape of the suture with the prefrontal is uncertain. The posterior suture with the parietal is transverse. On the ventral surface of the frontal, a longitudinal ridge defines the lateral margin of the sulcus olfactorius. The ridge is not developed posteriorly and the sulcus olfactorius is not properly defined by bone in the area of the frontal-parietal suture. The ridge increases gradually in height anteriorly up to the fossa nasalis. Anteriorly, the sulcus olfactorius is only formed by the frontal, with the prefrontal only bracing the lateral part of the ridge at the level of the fissura ethmoidalis. This anteroventral extension of the frontals fully separates the prefrontals on the ventral surface of the skull roof and reaches the nasals anteriorly. The description of this bone is consistent with what is known in other specimens referred to *Plesiochelys etalloni* (NMB 435, NMS 8738, and NMS 8739).

#### Parietal

The posterior part of the parietal is missing on both sides. On the skull roof, the parietal has a transverse anterior suture with the frontal and an anteromedially trending lateral suture with the postorbital (Fig 2A and 2B). Only a small portion of the posterior margin of the left parietal is natural and reveals that the upper temporal emargination exposes the foramen spatedio-temporale in dorsal view. Some scale sulci appear to be preserved on the parietal, but the bone surface is damaged and it is difficult to identify sulci with certainty. The ethmoid region is particularly well preserved in both NMS 40870 and NMS 40871 (Figs 3 and 4). The length of the processus inferior parietalis is reduced compared to most turtles (large foramen interorbitale), but not as much as in Pan-Chelonioidea [7]. Ventrally, the processus inferior parietalis has a broad contact with the epipterygoid. A short contact with the pterygoid excludes the epipterygoid from the anterior margin of the foramen nervi trigemini. The configuration is similar on the medial surface of the braincase wall (Fig 4G and 4H). The parietal forms the upper half, the dorsal part, and the entire posterior margin of the foramen nervi trigemini in lateral view. The long ventral process of the parietal posterior to the foramen nervi trigemini contacts the pterygoid and the quadrate, effectively excluding the prootic from the margin of this foramen in lateral view. NMS 40871 reveals that the pterygoid and prootic actually meet medial to the descending process of the parietal (see Prootic).

#### Jugal

Only the part of the jugal that frames the orbit is preserved on both sides (Figs 2 and 3). There, the jugal contacts the postorbital dorsally, the orbit anteriorly, and the maxilla ventrally. Medially, the jugal forms a sub-horizontal crest that forms the lateral margin of the foramen palatinum posterius and that was in contact with, or came very close to, the palatine anteromedially (Fig 2C–2D). This contact may not have been present in NMB 435, a juvenile individual in which the foramen palatinum posterius is larger. This area is missing in all other crania referred to *Plesiochelys etalloni*.

#### Quadratojugal

Only a small portion of the quadratojugal is preserved on both sides. The bone braces the cavum tympani anteriorly, but does not participate to its margin (Fig 3A–3B). Posteroventrally, the quadratojugal extends down to the level of the most ventral part of the cavum tympani, just above the condylus mandibularis. Anteriorly, the quadratojugal is a sheet-like element. The ventral margin of the left quadratojugal is natural and reveals the posterior outline of the lower temporal emargination. This description is broadly consistent with the morphology exhibited by NMB 435, but the lower temporal emargination appears to be slightly more developed in the latter specimen.

#### Squamosal

Both squamosals were entirely disarticulated and lost. Their contacts with the quadrate and opisthotic are clearly visible on the posterodorsal surface of the otic chamber (Fig 2A, 2B, 2G and 2H), but nothing can be said of their morphology.

#### Postorbital

The posterior half of each postorbital is missing on both side. On the skull roof, the postorbital contacts the frontal anteromedially and the parietal posteromedially, and forms the posterodorsal margin of the orbit (Fig 2). The orbital margin is damaged on the left hand side, leaving the misleading impression that the frontal-postorbital contact is absent. The anteroventral part of the right postorbital is severely damaged. However, the ventral contact with the jugal is visible on the left hand side. A low ridge extends on the ventral surface of the postorbital to mark the posterodorsal limit of the fossa orbitalis. This morphology agrees with what is known in other specimens referred to *Plesiochelys etalloni*, notably NMB 435.

#### Premaxilla

The parts of the premaxilla that form the anterior tip of the labial ridge and the floor of the fossa nasalis are missing. As preserved, the premaxilla contacts the maxilla laterally, the vomer posteriorly, and the other premaxilla medially (Fig 2C–2F). Posteriorly the premaxilla form most of the foramen praepalatinum. Interestingly, NMS 40870 exhibits a number of differences compared to NMB 435, a juvenile and the only other specimen referred to *Plesiochelys etalloni* where similar parts of the premaxilla are preserved. The most striking difference is the much stronger lingual ridge in NMS 40870. As a result, the median channel between the lingual ridges is much more narrow in that specimen. Ontogenetic changes in the development of labial and lingual ridges have been documented in Pan-Chelonioidea [51]. For that matter, there is therefore no real difference between *Plesiochelys etalloni* and *Portlandemys mcdowelli*. Based primarily on NMB 435, Gaffney [20] indicated that the maxilla formed the posterior edge of the foramen praepalatinum. This observation is correct for NMB 435, as long as the palatal view is concerned, but this contribution of the maxilla is only minimal in that specimen. In NMS 40870, the foramen praepalatinum is formed exclusively by the premaxilla and vomer, both in dorsal and palatal views. In NMS 8739 and '*Thalassemys*' *moseri*, the foramen praepalatinum is formed exclusively by the premaxilla (contra [39]). The condition in *Plesiochelys planiceps* and *Portlandemys mcdowelli* is similar to that exhibited by NMS 40870. A contribution of the maxilla to the foramen praepalatinum is therefore the exception, not the rule.

#### Maxilla

The maxillae are equally preserved on both sides. The maxilla contacts the premaxilla and vomer anteromedially, the nasal and prefrontal anterodorsally, the palatine medially, and the jugal posterodorsally (Figs 2 and 3). The bone forms the lateral margin of the foramen orbito-nasale and the ventral edge of the orbit. As for the jugal, the maxilla lacks a posteromedial process that would contact the pterygoid and close the foramen palatinum posterius posterolaterally. The maxilla does not enter the margin of this foramen in ventral view. In dorsal view, the lateral surface of the maxilla appears to be moderately concave anterolaterally. When preserved, the lateral surface of the maxilla is mostly straight in dorsal view in other specimens referred to *Plesiochelys etalloni* (NMB 435, NMS 8738, NMS 8739, and NMS 8740). A small foramen supramaxillare penetrates the maxilla just medial to the anteriormost extension of the jugal in the floor of the fossa orbitalis. The anterolateral margin of the left foramen orbito-nasale is damaged revealing a relatively large foramen alveolare superius (Fig 2A and 2B). This agrees with the condition described by Gaffney [20]. Similarly, as noted by Gaffney [20], there is a small foramen in the maxilla just outside the anteroventral corner of the orbit. This unnamed foramen seems to lead to a sub-vertical canal going down into the maxilla. A similar foramen exists in the same position in '*Thalassemys*' *moseri*. In *Portlandemys mcdowelli* and *Plesiochelys planiceps*, a potentially homologous foramen is present within the fossa orbitalis lateral to the foramen orbito-nasale. According to Gaffney [20], this foramen is also present in *Toxochelys* Cope, 1873 [52], but the systematic distribution and primary homology of this character should be further investigated. The labial ridge is slender and very high. It decreases in height anteriorly and it is convex ventrally in lateral view. The angle formed by the two labial ridges is more open than in *Portlandemys mcdowelli*, as already noted by Gaffney [20]. The trough separating the labial and lingual ridges is deep. The broad and high lingual ridge is entirely formed by the maxilla. It is stronger than in NMB 435. The latter specimen is a juvenile individual, as evidenced by its open sutures, so this difference in the development of the lingual ridge is probably linked to ontogeny. The lingual ridge is poorly preserved in other known skulls of *Plesiochelys etalloni*, which prevents comparison. The lingual ridge becomes less pronounced as it extends posteriorly and is generally lower than in *Plesiochelys planiceps*.

#### Vomer

The vomer is well preserved. Only a small part of the posteroventral median septum is missing (Fig 2C and 2D). Anterodorsally, the vomer forms the posteroventral wall of the fossa nasalis (Fig 2E and 2F). It contacts the premaxillae anteriorly and the maxillae laterally. Anteroventrally, the vomer forms the dorsal part of the foramina praepalatinum. More posteriorly, the dorsal surface of the vomer is gouged by a deep sulcus vomeri. The vomer meets the prefrontals at the level of the fissura ethmoidalis and this contact extends posteriorly into the interorbital region up to the level of the suture between the prefrontal and the palatine. There, the vomer separates the palatines for a short length then is completely covered by the meeting of these bones in the midline. In ventral view, the vomer separates the palatines for about half of their length and then forms an incipient sagittal septum that extends posteriorly on the ventral surface of these bones. This septum is mostly broken in NMS 40870. The vomer widens again posteriorly just anterior to the palatine-pterygoid suture. There again, the contact appears to be mostly with the ventral surface of the palatine and pterygoid.

#### Palatine

The palatine is well preserved on both sides (Fig 2C and 2D). It contacts the vomer anteromedially and ventromedially, the prefrontal anteriorly, the maxilla laterally, the pterygoid posteriorly, and the other palatine posteromedially, although this contact is usually masked in ventral view by the posteromedian extension of the vomer on its ventral surface (see Vomer). The palatine forms most of the posterior margin of the foramen orbito-nasale. Ventrally, the palatine contributes largely to the roof and lateral margin of the apertura narium interna. Posterolaterally, the palatine forms most of the edge of the open foramen palatinum posterius. This area is remarkably preserved in NMS 40870, notably on the left hand side, and can be compared in details with the same area in NMB 435. The foramen palatinum posterius is round instead of oval in the latter specimen. This foramen is also round in NMS 8739, a specimen of comparable size with NMS 40870 (Table 1), so this feature may be linked to ontogeny. This difference in shape is mostly the result of the greater posterolateral development of the process of the palatine that braces the posteriormost part of the lingual ridge. This process excludes the maxilla from the margin of the foramen palatinum posterius in ventral view. There was probably also a contact with the medial crest present on the jugal. This process of the palatine is absent in NMB 435, and this region is not sufficiently preserved in other skulls referred to *Plesiochelys etalloni*.

The dorsal surface of the palatine forms the medial part of the floor of the fossa orbitalis. The vomer separates the palatines medially only for the first half of their length. Posteriorly, the palatine has a broad contact with the pterygoid and forms the anterior part of a large concavity in front of the side wall of the braincase (see Pterygoid).

#### Quadrate

On the dorsal surface of the otic chamber, the quadrate contacts the prootic anteromedially, the opisthotic posteromedially, and the squamosal (now disarticulated and lost) posterolaterally (Fig 2A and 2B). Anterolaterally, the quadrate and quadratojugal are in contact along a long curved suture that is mostly parallel to the anterior margin of the cavum tympani, but lies just anterior to it (Fig 3). Ventromedially, the quadrate also has an extensive sutural contact with the pterygoid. In NMS 40870, the fossa cartilaginis epipterygoidei is obliterated and the quadrate has a short contact with the epipterygoid in the ethmoid region (Fig 3C and 3D). This contact is absent in NMS 40871, because the fossa cartilaginis epipterygoidei is not obliterated in this specimen (Fig 4E and 4F). As already noted above, the quadrate also contacts the posteroventral process of the parietal behind the foramen nervi trigemini, excluding the prootic from the margin of this foramen and preventing a contact between the prootic and pterygoid. The quadrate is involved in many structures of the otic chamber. It forms about half of the processus trochlearis oticum dorsally, slightly more ventrally. The processus trochlearis oticum is moderately developed, contrasting with the condition in *Portlandemys mcdowelli* and *Plesiochelys planiceps*. As in most turtles, the quadrate and prootic contribute equally to the formation of the foramen stapedio-temporale and canalis stapedio-temporalis.

Laterally, the quadrate also forms the cavum tympani, including all of its anterior margin. The incisura columellae auris remains open posteroventrally. A prominent ventrally-infolding ridge occurs on the posterior surface of the processus articularis of the quadrate (Fig 2G and 2H). This ridge extends dorsomedially from the posterolateral corner of the condylus mandibularis, then curves medially below the incisura columellae auris, and finally merges with the posterior margin of the pterygoid. A similar ridge is present in *Plesiochelys planiceps*, *Portlandemys mcdowelli*, *Portlandemys gracilis*, and *'Thalassemys' moseri*, but also in the eurysternids *Solnhofia parsonsi* Gaffney, 1975c [53] and *Parachelys eichstaettensis* Meyer, 1864 [54] (NHMUK 42888; JA, unpublish. data; see Discussion). The condylus mandibularis consists of two moderately concave facets separated by a relatively flat area. In NMS 8738, the two facets are separated by a very modest parasagittal furrow.

#### Columella auris

The columella auris, or stapes, is preserved on both sides (Figs 2 and 3). The lateral part of the right columella auris is broken at the level of the incisura columellae auris. The left columella is complete. As preserved, the columella auris curves around the incisura columellae auris. Since neither of the columellae auris is exactly in anatomical position, it is uncertain whether this curvature is natural. However, the columella auris of *Plesiochelys planiceps* is similarly curved around the incisura. The basis columellae is relatively thick and, as preserved, does not fit into the fenestra ovalis. Its medial surface is deeply concave and round in outline. The part of the columella auris lateral to basis columellae is mostly straight and round in section. Lateral to the incisura columellae auris, the columella flattens significantly and terminates by a slightly concave surface, which was undoubtedly extended in life by the cartilaginous extracolumella [55]. This is the first time that the lateral part of the columella auris is documented in plesiochelyids.

#### Epipterygoid

The epipterygoid is preserved in both NMS 40870 and NMS 40871 (Figs 2–4). The bone is located between the crista pterygoidea of the pterygoid and the processus inferior parietalis of the parietal and is roughly triangular in lateral view. The epipterygoid is exposed in the internal surface of the anterior braincase wall (Fig 4G and 4H). It contacts the pterygoid ventrally and posteriorly, and the parietal dorsally. In NMS 40870, there is also a short posteroventral contact with the quadrate as the fossa cartilaginis epipterygoidei is obliterated in this specimen. The fossa remains open in NMS 40871, preventing the epipterygoid-quadrate contact in this smaller specimen. Anteriorly, the epipterygoid forms the posteroventral margin of the foramen interorbitale. Posteriorly, it is prevented from entering the anterior margin of the foramen nervi trigemini by a contact between the parietal and pterygoid. The contacts are the same on the internal surface of the anterior braincase wall. As noted by Gaffney [20], there is a low longitudinal crest halfway up the posterior part of the internal surface of the epipterygoid (Fig 4G–4J).

#### Pterygoid

The pterygoids are better preserved in NMS 40870 (Figs 2 and 3) than in NMS 40871, but the latter provides valuable information on their morphology in the ethmoid region and inside the cavum epiptericum (Fig 4). On the ventral surface of the cranium, the pterygoid contacts the palatine anteriorly, the vomer anteromedially, the basisphenoid posteromedially, the basioccipital posteriorly, and the quadrate posterolaterally. Anterolaterally, the processus pterygoideus externus is well-developed. Its lateral margin is expended dorsally to form a low parasagittal plate. The anterolateral corner of the processus pterygoideus externus forms a small part of the posterior margin of the foramen palatinum posterius. There is no ossified contact with the jugal or maxilla in this region and the foramen palatinum posterius remains open posterolaterally. An arched ridge extends from the posteromedial border of the processus pterygoideus externus to the foramen posterius canalis carotici interni. This ridge becomes increasingly more pronounced posteriorly and forms the medial border of a deep pterygoid fossa. This fossa is proportionally less deep in NMS 40871, NMB 435, and NMS 8739 than in NMS 40870, which suggests that its development may be linked to ontogeny. The canalis caroticus internus is superficial. The anterior half of the canal runs along the basisphenoid-pterygoid suture, but remains open ventrally. Anteriorly, the split between the cerebral and palatine branches of the internal carotid artery is not concealed by bone ventrally. The foramen posterius canalis carotici cerebralis and foramen posterius canalis carotici palatinum are therefore apparent in ventral view (Fig 2C and 2D). The posterior half of the canalis caroticus internus is floored by a thin ventromedial flap of the pterygoid. The foramen pro ramo nervi vidiani is apparent on the left side of NMS 40870 at the point where the flap of the pterygoid starts to floor the canalis caroticus internus. The foramen posterius canalis carotici interni is formed by the pterygoid only, but the basisphenoid forms the medial part of the canalis caroticus internus internally (Figs 2C, 2D, 3E and 3F). NMS 40870 is the only available specimen referred to *Plesiochelys etalloni* that documents this area in such a pristine condition. Posterior to the foramen posterius canalis carotici interni, the pterygoid is a thin sheet of bone that wraps the anterolateral corner of the tuberculum basioccipitale. This sheet of bone is variously damaged in all other specimens referred to *Plesiochelys etalloni*, which explains diverging morphologies in these specimens. For example, NMS 40870 clearly shows that the pterygoid wraps itself around the tuberculum basioccipitale and meets the exoccipital laterodorsally. This contact was previously thought to be absent in *Plesiochelys etalloni* [20].

Between the basisphenoid and the quadrate, the pterygoid forms a well-developed pterygoid fossa and floors the cavum acustico-jugulare. The posterior margin of the pterygoid folds dorsally. Moderate medially, this infolding becomes more pronounced laterally and then merges with the ridge on the posterior surface of the quadrate. Inside the cavum acustico jugulare, the pterygoid has extensive contacts with the quadrate and prootic. The processus interfenestralis of the opisthotic also contacts the pterygoid.

Contacts of the pterygoid in the ethmoid region have already been described in detail under Epipterygoid and Parietal. As in the ventral surface, the pterygoid has a long transverse suture with the palatine anteriorly. In front of the anterior margin of the braincase wall, the pterygoid forms the posterior part of a pronounced depression. This depression likely served for the attachment of one of the eye muscles and is particularly well developed in plesiochelyids [20]. Medial to this concavity, the pterygoid forms a raised shelf that decreases anteriorly in width. This shelf is level with the sella turcica. The foramen anterius canalis carotici palatinum opens just anterior to the level of the foramen anterius canalis carotici cerebralis. The palatine branch of the internal carotid artery continues forward in a groove within the floor of the sulcus cavernosus. The foramen nervi vidiani can only be observed in NMS 40871 (Fig 4). On the left side, it opens in the aforementioned groove slightly anterior to the foramen anterius canalis carotici palatinum. On the right side of the specimen, it opens posterior to the foramen anterius canalis carotici palatinum within the canalis caroticus palatinum, and is only visible because the specimen is damaged in this area. In NMS 40871, the groove in which runs the palatine branch of the internal carotid artery extends anteriorly unobstructed and allows the artery to exit the braincase. In contrast, in NMS 40870, the crista pterygoidea extends anteromedially and merges with the raised midline shelf of the pterygoid, obstructing the path of the aforementioned groove. The palatine branch of the internal carotid artery seems to penetrate this wall and to exit at the base of the anteromedial extension of the crista pterygoidea (but see Discussion).

#### Supraoccipital

The supraoccipital forms the posterior part of the roof of the cavum cranii (Fig 2). It contacts the parietal anteriorly, the prootic anterolaterally, the opisthotic posterolaterally, and the exoccipital posteromedially. The dorsal part of the supraoccipital is damaged, and the crista supraoccipitalis is entirely broken. The dorsal half of the foramen magnum is also severely damaged, revealing part of the floor of the cavum cranii in dorsal view. A short, arched ridge is present on the dorsal surface of the supraoccipital close to the suture with the opisthotic (Fig 2A and 2B).

#### Exoccipital

The exoccipital contacts the supraoccipital dorsomedially, the opisthotic laterally, the pterygoid anteroventrally, and the basioccipital posteroventrally (Figs 2 and 3). Two foramina nervi hypoglossi perforate the exoccipital anterolateral to the upper part of the condylus occipitalis. The foramen jugulare posterius is formed by the exoccipital and remains open ventrolaterally. Ventral to the foramen jugulare posterius, the pterygoid wraps around the tuberculum basioccipitale and contacts the exoccipital (see Pterygoid above). This contact is however rather difficult to see and the thin sheet of the pterygoid that wraps around the tuberculum basioccipitale is variously damaged in other specimens referred to *Plesiochelys etalloni*, which explains why Gaffney [20] considered this contact to be absent. The exoccipital forms the anterodorsal portion of the neck of the condylus occipitalis, but is excluded from the condylus itself (Fig 2A and 2B). Gaffney [20] concluded that the exoccipitals formed the anterolateral portions of the condylus occipitalis in *Plesiochelys*, but his description was based on OUMNH J.1582, the holotype of *Plesiochelys planiceps*. This area is actually not sufficiently preserved in any specimen referred to *Plesiochelys etalloni*, except NMS 40870. The basioccipital separates the exoccipitals in the midline on the floor of the foramen magnum, but the exoccipitals do meet medially more anteriorly within the cavum cranii.

#### Basioccipital

The basioccipital contacts the basisphenoid anteriorly, the pterygoid anterolaterally, and the exoccipital dorsally (Fig 2). Internally, the basioccipital also has a sutural contact with the processus interfenestralis of the opisthotic, but this contact is only visible thanks to the sectioning of the left otic chamber in NMS 40870 (Fig 3E and 3F). As mentioned for the exoccipital, the basioccipital forms the entire condylus occipitalis and separates the exoccipitals medially on the floor of the foramen magnum. More anteriorly, the exoccipitals meet in the midline, although it is uncertain whether they do so up until the level of the anterior suture with the basisphenoid. It seems that they do not and that the basioccipital re-emerges at the level of the posterior border of the hiatus acusticus. However, the sutures on the floor of the cavum cranii are relatively difficult to make out. Anteriorly, the basis tuberculi basalis is relatively large and preceded by an oval cavity located on the basisphenoid-basioccipital suture.

#### Prootic

Externally, the prootic forms the anteromedial part of the otic chamber. There, it contacts the parietal anteromedially, the supraoccipital posteromedially, the opisthotic posteriorly, and the quadrate posterolaterally (Figs 2 and 3). Due to the overlapping nature of the sutures in this region, the development of the posterior contact with the opisthotic is variable among available specimens. This contact is relatively developed in NMB 435, moderately developed in NMS 40870, and absent or almost absent in the remaining referred specimens. In those specimens where this contact is absent, the supraoccipital and quadrate contact for a short length instead. The prootic forms about half of the moderately developed processus trochlearis oticum dorsally, slightly less ventrally. As in most turtles, the prootic and quadrate contribute equally to the formation of the canalis stapedio-temporalis and foramen stapedio-temporale. In lateral view, a large descending process from the parietal excludes the prootic form the posterior margin of the foramen nervi trigemini and prevents a contact between the prootic and pterygoid in this region (Figs 3 and 4). Medial to this process of the parietal, the prootic does meet the pterygoid and enters the posteromedial margin of the foramen nervi trigemini. This configuration is particularly well illustrated by specimen NMS 40871.

Internally, the prootic forms the anterolateral wall of the cavum cranii and accommodates the anterior part of the inner ear. There, the prootic contacts the quadrate anterolaterally, the pterygoid anteroventrally, the opisthotic posterolaterally, the supraoccipital posteromedially, the parietal anteromedially, and the basisphenoid ventromedially. The internal aspect of the prootic is better seen in NMS 40871 (Fig 4G and 4H). The prootic forms the dorsomedial roof of the canalis cavernosus. Anteromedially, the prootic sends a flat ventromedial process that articulates with the processus clinoideus of the basisphenoid. Gaffney [20] interpreted this ossified band as a complete ossification of the pila prootica. A foramen perforates the dorsal part of this process. As far as we know, the complete ossification of the pila prootica is unique to *Plesiochelys etalloni* (see Discussion). This ossified band defines a large opening in the anterolateral wall of the cavum cranii. This opening is bordered mainly by the prootic with a minor anteromedial contribution from the basisphenoid. The purpose of this opening is probably to allow the passage of the ramus maxillaris (V2) and mandibular ramus (V3) of the trigeminal (V) nerve toward the foramen nervi trigemini.

#### Opisthotic

The opisthotic contacts the prootic anteriorly (but see Prootic), the supraoccipital medially, the exoccipital posteriorly and posteroventrally, the squamosal posterolaterally, and the quadrate anterolaterally (Figs 2 and 3). The processus interfenestralis also has sutural contacts with the pterygoid, the basioccipital, and the exoccipital. The dorsal base of the foramen is pierced by the foramen externum nervi glossopharyngei. The processus interfenestralis is relatively poorly preserved in NMS 40870. On the left, sectioned side, the fenestra perilymphatica is apparently visible, but its contours are uncertain. On the right side of the cranium, a small opening is present in the ventral margin of the processus interfenestralis and may correspond to the hiatus postlagenum.

#### Basisphenoid

In ventral aspect, the basisphenoid contacts the pterygoid anterolaterally and the basioccipital posteromedially. The bone is roughly triangular in outline. NMS 40870 is undoubtedly the best specimen documenting this area (Fig 2C and 2D). As described above under Pterygoid, the anterior half of the canalis caroticus internus is not floored exposing the foramen posterius canalis carotici cerebralis and the foramen posterius canalis carotici palatinum in ventral view. These foramina open anteriorly in the basisphenoid. The anterior portion of the canalis caroticus internus runs just medial to the basisphenoid-pterygoid suture, and its roof is formed mostly by the basisphenoid. The posterior part of the canalis caroticus internus is floored by a ventromedial flap of the pterygoid. Although the foramen posterius canalis carotici interni is formed only by the pterygoid, the sectioned left otic chamber reveals that the basisphenoid forms the medial wall of the canalis caroticus internus up to the level of the foramen (Fig 3E and 3F). The ventral surface of the basisphenoid is slightly concave. The basioccipital is disarticulated in NMS 40871, revealing that the parasphenoid and basisphenoid actually remain distinguishable posteriorly. A similar condition was extensively described in NMB 435 [21].

The dorsal aspect of the basisphenoid is better preserved in NMS 40871 (Fig 4). The following description is therefore based primarily on that specimen, but also applies to NMS 40870 in most cases. As in most turtles, the basisphenoid forms the anterior part of the floor of the cavum cranii. This part of the basisphenoid is dorsally concave. The canalis nervi abducentis opens posteriorly about halfway between the posterior suture with the basioccipital and the dorsum sellae anteriorly. Laterally, the basisphenoid has a broad sutural contact with the prootic. Anterolaterally, the processus clinoideus is also sutured to a flat process of the prootic, this structure being interpreted as a complete ossification of the pila prootica ([20]; see Prootic). The anterior foramen nervi abducentis opens ventral and posterior to the base of the processus clinoideus, close to the suture between the basisphenoid and the pterygoid. The dorsum sellae is high and does not overhang the sella turcica. As noted by Gaffney [20], this is a peculiar arrangement that *Plesiochelys* and *Portlandemys* share with Pan-Chelonioidea (see Discussion). The bone surface below the dorsum sellae is almost vertical dorsally, then slopes anteroventrally in its lower half. A distinct sagittal ridge is present on this surface. Anteroventrally, this surface forms a horizontal shelf between the two trabeculae. The foramina anterius canalis carotici cerebralis open just underneath this shelf and lie relatively close to one another. As a result, the foramina anterius canalis carotici cerebralis open a short distance anterior to the level of the dorsum sellae, but not as far anteriorly as in *Portlandemys mcdowelli* and *Portlandemys gracilis* (see below). The trabeculae are straight and do not constrain the sella turcica anteriorly. The floor of the sella turcica is level with the midline triangular shelf formed by the pterygoid anteriorly. The extension of the basisphenoid beyond the anterior tip of the trabeculae is very limited.

### The cranium of *Portlandemys gracilis* (MJSN BSY009-708)

#### General description

MJSN BSY009-708 is an isolated, partial cranium missing the posterolateral skull roof, the orbital region, the palate, the upper jaw, and the nasal area (Figs 5–7). The premaxilla, the maxilla, the vomer, and the jugal are lacking. Three disarticulated bones associated with the skull may represent fragments of the palatines and right postorbital (Fig 6G and 6J), although it is ultimately unsure whether these pertain to the same individual. The skull has been severely crushed dorsoventrally with a slight translation toward the left side of the specimen (S5 File). During preparation, the remaining part of the skull roof and the basicranium were glued back together (Fig 5). In order to describe the cavum epiptericum and the area of the sella turcica, these two fragments were later separated, which revealed that there was truly no natural surface of contact between them. Despite being slightly incorrect, the initial configuration with the two parts glued together gave a rough idea of the length of the skull, which is about 92 mm from the nasal to the condylus occipitalis. The width taken at the level of the condyli mandibularis is 73.4 mm. These dimensions are comparable to those of NHMUK R2914, the holotype of *Portlandemys mcdowelli*. Both *Portlandemys mcdowelli* and *Portlandemys gracilis* have proportionally narrower skulls (higher length/width ratio) than *Plesiochelys etalloni*, *Plesiochelys planiceps*, and '*Thalassemys*' *moseri* (Table 1).

#### Nasal

Only the left nasal is preserved, but it is fairly complete (Fig 5). The nasals contacted one another in the midline along their entire length. As preserved, the nasal also contacts the prefrontal posterolaterally and the frontal posteriorly. In *Portlandemys mcdowelli* (NHMUK R2914), *Plesiochelys etalloni*, and *Plesiochelys planiceps*, the nasal-frontal contact is prevented in dorsal view by a contact of the prefrontals in the midline. In '*Thalassemys*' *moseri*, a nasal-frontal contact prevents the prefrontals from meeting in the midline in dorsal view. The anterolateral part of the left nasal is slightly damaged. The nasal is sub-quadrangular and longer than wide, although this is not readily apparent in dorsal view as the bone dips anteriorly (Fig 6C and 6F). In ventral view, the nasal also contacts the prefrontal and the frontal, although the contact with the former is more extensive and the contact with the latter reduced. The ventral surface of the nasal is concave and forms the roof of the fossa nasalis. The median surface of the left nasal reveals how the frontal extends beneath the nasal anteriorly up to half the dorsal length of this bone (Fig 6E and 6F).

#### Prefrontal

Only a small portion of the left prefrontal is preserved in MJSN BSY009-708 (Fig 5). It consists of the most dorsal and medial part of the prefrontal, where the bone meets the nasal anteromedially and the frontal posteromedially. In dorsal view, the prefrontals were separated from one another by a broad median contact of the nasals and frontals, which contrasts with the condition observed in *Portlandemys mcdowelli* (NHMUK R2914). In ventral view, the nasal-frontal contact is more reduced, but still present. The ventral surface of the prefrontal is concave anteromedially, where it forms the posterolateral corner of the roof of the fossa nasalis, and posterolaterally, where it forms part of the orbital margin. Between these two concave areas, the dorsomedial part of the descending process of the prefrontal is still visible.

#### Frontal

Both frontals are equally preserved and almost complete (Figs 5 and 6). They contact one another medially along their entire length. Anteriorly, the frontal has a broad, transverse contact with the nasal. As noted above (see Nasal), this contact is more reduced in ventral view and the frontal actually extends anteriorly beneath the nasal. The suture between the prefrontal and frontal is relatively short and oblique in dorsal view. Ventrally the prefrontal is more developed and this suture is longer. Laterally, the frontal forms the posterodorsal margin of the orbit. The suture with the postorbital (not articulated) is better preserved on the left side of the specimen. Posteriorly, the frontal meets the parietal along a strongly interdigitating suture that is slightly concave posteriorly. Ventrally, the frontals form most of the sulcus olfactorius, the ridges of which become increasingly higher anteriorly. Although MJSN BSY009-708 and NHMUK R2914 are about the same size (Table 1), the thickening of the interorbital region of the skull roof is less developed in the former than in the latter.

#### Parietal

The parietals are equally preserved on both sides (Figs 5 and 6). On the skull roof, the parietals are almost complete. A few scale sulci are visible on the dorsal surface of the parietal. The parietal contacts the frontal anteriorly and the postorbital laterally. It is unknown whether a posterolateral contact with the squamosal was present. Posteriorly, the parietal forms the medial half of a moderately developed upper temporal emargination. This emargination must have revealed most of the floor of the upper temporal fossa in dorsal view. The upper temporal emargination is more reduced in *Plesiochelys etalloni* (NMB 435, NMS 40870), *Plesiochelys planiceps*, and *'Thalassemys' moseri*. In these taxa, the posteromedial margin of the parietal curves sharply laterally, whereas in MJSN BSY009-708 this margins arcs more gently anterolaterally, extending the emargination further anteriorly. In NHMUK R2914, the holotype of *Portlandemys mcdowelli*, the extension of the upper temporal fossa is uncertain because the posterior and lateral parts of the parietal are damaged. The upper temporal emargination appears to be more reduced in NHMUK R3164 than in MJSN BSY009-708. However, it should be noted that the skull NHMUK R3164 belonged to a young individual and is much smaller than MJSN BSY009-708 and NHMUK R2914 (Table 1). It is uncertain if and how ontogeny would have affected this morphology.

On the ventral surface of the skull roof, the dorsal part of the processus inferior parietalis is preserved. A prominent ridge extends ventrally on the lateral surface of the processus, buttressing it against the skull roof dorsally. A similar ridge is also present in *Portlandemys mcdowelli* (NHMUK R2914). A large foramen opens anterior and dorsal to that ridge on the right side of the specimen. A similar pair of foramina occurs in OUMNH J.1582 (holotype of *Plesiochelys planiceps*), but they are located more posteriorly and face toward the rear of the skull. Posterior to the level of the prominent ridge described above, a ridge extending anterodorsally is also present in the roof of the cavum cranii.

The ventral part of the processus inferior parietalis is preserved in the basicranium fragment of MJSN BSY009-708 (Figs 6 and 7). The parietal forms a prominent part of the foramen nervi trigemini. Anterior to the foramen nervi trigemini, the parietal sends a process that meets the pterygoid about halfway up the foramen, excluding the epipterygoid from the foramen margin. The parietal also sends a long posteroventral process forming most of the posterior margin of the foramen nervi trigemini. This process contacts the pterygoid and quadrate ventrally and excludes the prootic from the margin of the foramen nervi trigemini. This remarkable configuration of the parietal around the foramen nervi trigemini also occurs in *Plesiochelys etalloni*, *Portlandemys mcdowelli*, *'Thalassemys' moseri*, and to some extent in *Plesiochelys planiceps* (see Discussion).

#### Quadratojugal

On the left side of the skull, only a strip of the quadratojugal remains bracing the quadrate and the cavum tympani. The right quadratojugal is slightly more complete (Figs 5 and 6). The quadratojugal has a long and arched posterior suture with the quadrate, but never enters the margin of the cavum tympani. The posteroventral process of the quadratojugal is long and extends down to the level where the processus mandibularis of the quadrate starts to inflate to form the condylus mandibularis. Posterodorsally, the quadratojugal meets the squamosal above the cavum tympani. This region is deformed in MJSN BSY009-708, but it seems that the suture with the squamosal is not located as posteriorly as it is in *Plesiochelys etalloni* and *'Thalassemys' moseri*. The suture with the squamosal is oblique in lateral view and extends anterodorsally from the rim of the cavum tympani (Fig 6C–6D). This is different from *Plesiochelys etalloni* (NMB 435, NMS 9145), in which the suture extends mostly dorsally and slightly posteriorly. This region is not preserved in specimens referred to *Portlandemys mcdowelli* and *Plesiochelys planiceps*, preventing comparison.

#### Squamosal

Most of the right squamosal is missing. The bone is better preserved on the left side of the skull, but the dorsal part is broken (Figs 5–7). As in most turtles, the squamosal contacts the quadrate anteriorly and ventrally, the quadratojugal anterolaterally, and the opisthotic medially. It is unknown whether the squamosal also contacted the postorbital or the parietal anterodorsally. The squamosal extends farther posteriorly than it does in *Plesiochelys etalloni* and *'Thalassemys' moseri*. Despite this posterior extension, the antrum postoticum is moderately developed. Posteriorly, the squamosal forms a large rugose area for the attachment of the M. depressor mandibulae (Fig 5E and 5F; see [56]). Anterior and lateral to this rugose area, the surface of the squamosal forms a wide concavity (Fig 6C and 6D).

#### Postorbital

An isolated bone associated with the basicranium and skull roof probably corresponds to a fragment of the right postorbital (Fig 6I and 6J). The coloration of this isolated bone is light brown when the rest of the skull is much darker, but changes in coloration are common in fossils from the Lower *Virgula* Marls. Furthermore, this fragment cannot be articulated with the rest of the skull. Therefore, it remains possible that this bone pertains to a different individual. It consists of a slightly curved, triangular sheet of bone. A dorsoventral ridge occurs on the internal surface of the bone. This structure probably corresponds to the ridge that extends along the posterior margin of the orbit on the internal surface of the postorbital in many turtles. This bone fragment does not reveal much valuable information.

#### Palatine

In addition to the element described above as a potential fragment of the right postorbital, two further isolated bones were found in association with the skull MJSN BSY009-708. Similarly, these two additional fragments are light brown in color and cannot be articulated with the rest of the skull (Fig 6G and 6H). Therefore, it is unsure whether they pertain to the same individual. These two bones are tentatively identified as fragments of the left and right palatines. The right palatine is more complete and consists of the anteromedial part of the bone. The left palatine consists only of the anterior half of the right element. The ventral surface of the palatine shows a long median suture with the vomer. Posteriorly, the palatines probably contacted one another in the midline beneath the vomer. Anteriorly, the palatine has two oblique sutural contacts. The anteromedial contact was probably with the vomer, whereas the anterolateral was certainly with the prefrontal. Posterolateral to the latter contact, the palatine forms the posteromedial margin of the foramen orbito-nasale. The remaining borders of each palatine are broken. A sub-transverse ridge occurs posteriorly on the dorsal surface of the right palatine. This ridge is slightly concave posteriorly and corresponds to the anterior border of the shallow concavity that occurs on the dorsal surface of the pterygoid and palatine notably in *Portlandemys mcdowelli*, *Plesiochelys planiceps*, and to a lesser extent in *Plesiochelys etalloni*.

#### Quadrate

Because of the dorsoventral compression of the skull during fossilization, the anterior surface of the quadrate below the processus trochlearis oticum now faces mostly ventrally, instead of anteriorly and slightly ventrally as in most turtles. Similarly, the cavum tympani collapsed as a consequence of this deformation (Fig 6C–6F; S5 File). In addition to the usual contacts with the pterygoid, the prootic, the opisthotic, the squamosal, and the quadratojugal, the quadrate also articulates the descending process of the parietal that forms most of the posterior margin of the foramen nervi trigemini. A similar contact occurs in *Plesiochelys etalloni*, *Portlandemys mcdowelli*, and *'Thalassemys' moseri*, but is notably absent in *Plesiochelys planiceps*. The quadrate forms a large part of the processus trochlearis oticum: about two thirds of the process in dorsal view, and most of it in ventral view (see Prootic; Fig 5A–5D). The processus trochlearis oticum is proportionally similar in development to that of *Plesiochelys etalloni* and *'Thalassemys' moseri*. Clearly, it does not reach the proportions observed in either *Portlandemys mcdowelli* or *Plesiochelys planiceps*, in which the processus is relatively robust. The processus trochlearis oticum of MJSN BSY009-708 is narrow. For example, NMS 40870, which is of comparable size with MJSN BSY009-708 (see Table 1), has a significantly wider processus trochlearis oticum. MJSN BSY009-708 is also characterized by the presence of two arched ridges on the dorsal surface of the processus trochlearis oticum, defining the lateral margins of the trochlea. These two ridges converge anteroventrally and form a broad U-shaped margin as seen in anterior view.

The cavum tympani is well developed. The incisura columellae auris is damaged on each side, but on the right side of the skull it is apparent that it remained open posteroventrally. The quadrate forms the entire border of the cavum tympani, but the squamosal enters the lateral margin of the antrum postoticum posterodorsally (Fig 6C and 6D). The part of the quadrate that lies posterior and dorsal to the incisura columellae auris and that meets the squamosal and the processus paroccipitalis of the opisthotic served for muscular attachment, as can be seen by its rugose surface. A prominent ventrally-infolding ridge occurs on the posterior surface of the processus articularis of the quadrate (Fig 5E and 5F). This ridge extends dorsomedially from the posterolateral corner of the condylus mandibularis, then curves medially below the incisura columellae auris, and finally merges with the posterior margin of the pterygoid. As already mentioned above, this ridge is known in all plesiochelyids, but also in '*Thalassemys*' *moseri* and at least some eurysternids (see Discussion). The ventrolateral extremity of this ridge is significantly developed and forms a rounded process in the posterolateral corner of the condylus mandibularis. This rounded process is notably apparent in ventral view (Fig 5C and 5D). The condylus mandibularis is better preserved on the right side of the cranium. It consists of two concave facets separated by a low parasagittal ridge. The referred mandible MJSN SCR011-441 has a corresponding furrow on the area articularis mandibularis along the surangular-articular suture (see below). The articular surface of the condylus mandibularis is notably smaller than in *Plesiochelys etalloni*. For example, the articular surface is about 11.5 mm wide in MJSN BSY009-708 compared to 16.7 mm wide in NMS 40870, although the former specimen is slightly larger than the latter. The condylus mandibularis is not known in *Portlandemys mcdowelli*.

#### Epipterygoid

Only fragments of the epipterygoid are preserved. The exact limits of the bone are difficult to interpret, but it is clear that it is excluded from the anterior margin of the foramen nervi trigemini by a contact between the parietal and the pterygoid. This condition is found in all plesiochelyids, as well as in '*Thalassemys*' *moseri*.

#### Pterygoid

The anterior part of the pterygoid where the bone usually expands laterally to form the processus pterygoideus externus is broken on each side (Fig 5C and 5D). On the ventral surface, a parasagittal ridge extends from the lateral border of the pterygoid waist anteriorly almost to the posteromedial margin of the bone. A second ridge extending obliquely on the quadrate process of the pterygoid meets anteriorly with the previous one approximately at the level of the anterior tip of the basisphenoid (as seen in ventral view). These ridges define a well-developed, triangular pterygoid fossa. In contrast to *Portlandemys mcdowelli*, this fossa is not bordered posteriorly by a ridge formed by the posterior margin of the pterygoid. Posteromedially, the pterygoid has a broad sutural contact with the basioccipital. The foramen posterius canalis carotici interni is formed entirely by the pterygoid and opens within the posterior margin of the bone. It is barely apparent in ventral view and located a relatively long distance from the basisphenoid-pterygoid suture. This contrasts from *Portlandemys mcdowelli* in which the foramen posterius canalis carotici interni is located closer to the basisphenoid-pterygoid suture and is clearly visible in ventral view. On the left side of the specimen, a crack reveals the course of the canalis caroticus internus for a very short length. Shortly after entering the foramen posterius canalis carotici interni, the internal carotid artery curves sharply medially and does not follow the course of the aforementioned crack as seen in ventral view. The canalis caroticus internus is otherwise entirely contained within bone.

The dorsal aspect of the posterior margin of the pterygoid is remarkable in MJSN BSY009-708. There, the pterygoid forms a sort of raised pedestal that probably met the processus interfenestralis of the opisthotic dorsally. In *Portlandemys mcdowelli*, this area is also notably thickened and a contact with the processus interfenestralis is also present. However, MJSN BSY009-708 is unique in that a deep fossa is developed ventral to the aforementioned pedestal and lateral to the foramen posterius canalis carotici interni (Fig 5E and 5F). The rest of the cavum acustico-jugulare is crushed dorsoventrally and it is uncertain whether the pterygoid contacted the exoccipital there, as in *Portlandemys mcdowelli*.

On the anterodorsal surface of the pterygoid, the foramen anterius canalis carotici palatinum opens in the floor of the sulcus cavernosus at about the same level as the foramen anterius canalis carotici cerebralis. As in *Plesiochelys etalloni* (see above), the palatine branch of the internal carotid artery continues forward in a groove running parallel to the trabecula. Approximately halfway between this foramen and the anterior tip of the trabecula a smaller foramen opens in the lateral wall of this groove. This foramen corresponds to the foramen nervi vidiani (see Discussion).

#### Supraoccipital

The supraoccipital contacts the parietal anteriorly, the prootic anterolaterally, the opisthotic posterolaterally, and the exoccipital posteriorly (Fig 5). In contrast to *Portlandemys mcdowelli* and most specimens of *Plesiochelys etalloni*, the supraoccipital does not reach the quadrate laterally to form a tetraradiate suture with the prootic and opisthotic. In this matter, MJSN BSY009-708 is similar to *Plesiochelys planiceps* and *'Thalassemys' moseri*. Posteriorly, the supraoccipital forms the dorsal margin of the foramen magnum. The crista supraoccipitalis is broken in MJSN BSY009-708. Therefore, it is unclear how much it projected posteriorly. As a result of postmortem compression, parasagittal cracks occur on each side at the base of the crista supraoccipitalis and the medial part of the supraoccipital has been pushed down into the cavum cranii.

#### Exoccipital

The exoccipital forms the dorsolateral and lateral margins of the foramen magnum (Fig 5). It contacts the supraoccipital dorsally, the opisthotic dorsolaterally, and the basioccipital ventrally. For the most part, the ventral suture with the basioccipital is not visible. It is therefore unclear to which extent the exoccipital contributed to the condylus occipitalis and whether it contacted the other exoccipital medially hence excluding the basioccipital from the margin of the foramen magnum, as in some plesiochelyids. Two foramina nervi hypoglossi occurs on each side. The cavum acustico-jugulare collapsed during the postmortem compression of the skull, so the contacts of the exoccipital in this region are difficult to assess. Anterior to the tuberculum basioccipitale, the exoccipital and basioccipital continue forward as an indistinct sheet of bone. This structure appears to contact what remains of the processus interfenestralis of the opisthotic, but it is unclear which of the basioccipital or the exoccipital actually comes into contact with the processus.

#### Basioccipital

In ventral view, the basioccipital contacts the basisphenoid anteromedially and the pterygoid anterolaterally (Fig 5C and 5D). Dorsally, it contacts the exoccipital, but the suture with this bone is mostly effaced. As the cavum acustico-jugulare has collapsed during the postmortem compression of the skull, the contacts of the basioccipital in this region are not apparent (see Exoccipital). As preserved, the tubercula basioccipitale are flatten sheet of bone, but this is the result of the deformation endured by the skull during fossilization. The condylus occipitalis is set on a relatively long neck and protrudes significantly from the occipital plan (Fig 5A and 5B).

#### Prootic

The prootic forms the anteromedial part of the otic chamber. On its dorsal surface, it contacts the parietal anteriorly and anteromedially, the quadrate laterally and anteroventrally, the opisthotic posteriorly, and the supraoccipital posteromedially (Fig 5A and 5B). The prootic forms the anteromedial half of the large foramen stapedio-temporale. In other plesiochelyids, the prootic usually forms about half of the processus trochlearis oticum. In MJSN BSY009-708, the contribution of the prootic is comparatively reduced. In dorsal view, the prootic forms only one third of the processus. The prootic-quadrate suture curves sharply medially as it reaches the anterior border of the processus and the processus trochlearis oticum is almost entirely formed by the quadrate in ventral view. As described above (see Parietal), the prootic is excluded from the posterior margin of the foramen nervi trigemini by a contact of the descending process of the parietal with the quadrate and pterygoid.

As noted above, the cavum acustico-jugulare collapsed during fossilization. The sutures of the prootic in this cavity are now mostly obliterated. However, the aditus canalis stapedio-temporalis and the posterior opening of the canalis cavernosus, structures usually formed at least in part by the prootic, are still observable. As in all plesiochelyids in which these structures can be observed, the aditus canalis stapedio-temporalis opens more posteriorly than the posterior opening of the canalis cavernosus (i.e., not in the roof of the latter). In *Portlandemys mcdowelli* and *Portlandemys gracilis*, the distance between the aditus canalis stapedio-temporalis and the posterior opening of the canalis cavernosus is greater than in *Plesiochelys etalloni* and *Plesiochelys planiceps*.

#### Opisthotic

On the dorsal surface of the otic chamber, the opisthotic contacts the prootic anteriorly, the quadrate anterolaterally, the squamosal posterolaterally, the supraoccipital medially, and the exoccipital posteromedially. The processus paroccipitalis is well-developed posteriorly and forms a distinct crest for muscular attachment posterolaterally (Fig 5). Ventral to this crest, a relatively large rugose area extends the one already described on the quadrate. This area probably also served for muscular attachment. This part of the opisthotic is relatively thickened. Medially and anteromedially, the thickness of the bone decreases rapidly. From there, the opisthotic continues anteromedially to form most of the roof of the cavum acustico-jugulare. In ventral view, the opisthotic contacts the squamosal posterolaterally, the quadrate laterally, and the exoccipital medially. Anteriorly, the opisthotic must have contacted the prootic, but this contact is now obliterated because the cavum acustico-jugulare has collapsed on each side following postmortem compression. The dorsal part of the processus interfenestralis pierced by the foramen externum nervi glossopharyngei is visible on each side of the skull. The ventral part of each processus interfenestralis is broken, but comparison with *Portlandemys mcdowelli* (NHMUK R2914 and NHMUK R3164) suggests that the processus contacted, or came in close juxtaposition with, the pterygoid (raised pedestal) and the basioccipital and/or exoccipital (see Exoccipital).

#### Basisphenoid

In ventral view, the basisphenoid is sutured to the pterygoid anteriorly and laterally. Posteriorly, the basisphenoid has a transverse suture with the basioccipital. This suture is difficult to follow in the midline. In other plesiochelyids, the ventral aspect of the basisphenoid is usually triangular in shape. In MJSN BSY009-708, the basisphenoid is more ogival in shape with parasagittal lateral sides and a rounded anterior part (Fig 5C and 5D). The ventral surface of the basisphenoid is slightly concave and there are three small, shallow cavities, two that are paired and located around the middle of the bone, and one posteriorly in the midline. Posteriorly, the basisphenoid is narrower than the basioccipital.

As described above, the medial part of the supraoccipital has collapsed inside the cavum cranii and this cavity remains mostly hidden from investigation. However, as the skull roof is partly broken and disarticulated anteriorly, the cavum epiptericum and the area of the sella turcica can be described in details (Fig 6A and 6B). The processus clinoideus are very large. As preserved, their base is broken and they now rest flat above the posterior part of the sulcus cavernosus. During life, they must have extended high and dorsolaterally, in an arrangement similar to the condition observed in *Portlandemys mcdowelli* and *Plesiochelys planiceps*. The pila prootica is not ossified. The anterior foramen nervi abducentis opens ventral and slightly anteromedial to the base of the processus clinoideus relatively close to the suture with the pterygoid. The dorsum sellae is high. The surface below the dorsum sellae slopes gently anteriorly and extends up to half of the distance between the dorsum sellae and the anterior tip of the trabeculae. An acute sagittal ridge occurs on this surface. Anteroventrally, the surface forms a horizontal shelf between the trabeculae. The foramina anterius canalis carotici cerebralis are located just beneath this shelf and lie relatively close to one another. Comparatively, they open further anteriorly from the dorsum sellae than in *Plesiochelys etalloni* or *Plesiochelys planiceps*, and this arrangement clearly recalls the condition in *Portlandemys mcdowelli* (see Discussion). The foramina anterius canalis carotici cerebralis are located at the same level as the foramina anterius canalis carotici palatinum in MJSN BSY009-708. As a result of the long, sloping surface below the dorsum sellae, the trabeculae appear stout and short. They are straight and do not constrain the sella turcica anteriorly. The basicranium is broken just anterior to the tip of the trabeculae. The anterior extent of the basisphenoid is therefore uncertain.

### The mandible of *Portlandemys gracilis* (MJSN SCR011-441)

#### General description

MJSN SCR011-441 consists of a complete mandible (Fig 8), which has suffered only minor postmortem deformation, resulting in a slight counterclockwise rotation of the left ramus around its anteroposterior axis (as seen in posterior view). Otherwise, the mandible is complete and pristine. The length of the mandible is 87.7 mm (measured on the left ramus). The length of the symphysis is 15.7 mm. The maximal width at the level of the areas articularis mandibularis is 67.2 mm. With these dimensions, the mandible fits almost perfectly with the cranium MJSN BSY009-708. The triturating surface of this mandible is relatively different from that of the holotype of *Portlandemys mcdowelli*, but the angle formed by the labial ridges is remarkably acute (40° in MJSN SCR011-441 and 45° in NHMUK R2914) compared to that of other plesiochelyids (65° in OUMNH J.1582 and NMB 435, 70° in NMS 8738 and NMS 8740). We are also confident about the attribution of this mandible to *Portlandemys gracilis* because of the presence of a parasagittal furrow on the area articularis mandibularis. This furrow corresponds to the low parasagittal ridge that separates the two facets of the condylus mandibularis of the quadrate in MJSB BSY009-708 (see above).

#### Dentary

As noted above, the mandible is complete. It is characterized by its low profile in lateral view and by the broadly rounded outline of the labial ridge anteriorly (Fig 8; S6 File). The dentary forms the anterior half of the mandible and bears all of the triturating surface. The labial ridge is relatively low, but increases slightly in height anteriorly. There is no symphyseal hook anteriorly. In lateral view, the labial ridge is flat. The lingual ridge is poorly developed and is barely visible on the symphysis. However, the triturating surface is expanded posteromedial to the lingual ridges in this region, forming a flat, triangular area crossed by a broad, low median ridge. The triturating surface is therefore broad in the symphysis region. In *Portlandemys mcdowelli*, the triturating surface is much more strongly developed with high labial and lingual ridges and a strong symphyseal hook. During life, a large part of the dentary was covered by a horny beak, the rhamphotheca, as apparent from the numerous small nutrient foramina on the lateral surface of the dentary. Just behind this area, a large foramen dentofaciale majus opens on the dentary. On the medial surface of the mandible, the dentary forms most of the sulcus cartilaginis meckelii and the dorsal margin of the foramen intermandibularis medius. The dentary contacts the coronoid posterodorsally, the surangular posterolaterally, the angular ventromedially and posteroventrally, and the splenial medially. There was probably also a contact with the prearticular internally, but it is not apparent externally.

#### Splenial

A large, plate-like, triangular splenial is present. The splenial is more trapezoidal in outline in *Plesiochelys etalloni* and *Portlandemys mcdowelli*. In *Plesiochelys planiceps*, the splenial is also triangular, but the posterior half is more extended than the anterior part. In the mandible MJSN SCR011-441, the anterior part of the splenial is the longest (Fig 8G and 8H). The splenial contacts the coronoid dorsally, the prearticular posterodorsally, the angular ventrally, and probably the dentary ventrolaterally (not visible externally). Anterodorsally, it forms the lower margin of the foramen intermandibularis medius and the posteroventral margin of the sulcus cartilaginis meckelii. Anteroventrally, the splenial extends as far as the angular along the ventral margin of the sulcus cartilaginis meckelii. An oval foramen intermandibularis oralis opens on the suture between the splenial and the angular ventral and slightly anterior to the foramen intermandibularis medius.

#### Angular

The angular bone is remarkable by its anterior extension, almost reaching the symphysis anteriorly (Fig 8). On the left ramus, only the articular surface between the anterior tip of the angular and the dentary is visible, but this part of the bone is complete on the right ramus. The angular clearly extends farther anteriorly than the splenial. The condition of the angular in *Portlandemys mcdowelli* is poorly known. Parsons and Williams [18] concluded that the angular was absent in NHMUK R2914, whereas this bone was illustrated, but not discussed, by Gaffney ([20], fig 50). Based on our observations, the angular is present in *Portlandemys mcdowelli* (NHMUK R2914) and extends anteriorly as a narrow sheet of bone as far as the anteroventral tip of the splenial. A configuration that recalls that in *Portlandemys gracilis*. In *Plesiochelys planiceps*, the angular extends anteriorly up to the level of the foramen intermandibularis medius, whereas in *Plesiochelys etalloni* the angular only extends halfway along the splenial. The angular curves around the ventral margin of the ramus from the medial surface of the ramus anteriorly to the lateral surface posteriorly. The width of the bone increases posteriorly. It contacts the dentary anterolaterally for most of its length, the splenial anterodorsally, the prearticular posterodorsally on the medial surface of the ramus, the surangular posterodorsally on the lateral surface of the ramus, and the articular posteriorly. The contact between the angular and surangular prevents the dentary from reaching the articular posterolaterally. As noted above, the foramen intermandibularis oralis opens on the suture between the angular and splenial. Similarly, a large foramen intermandibularis caudalis opens more posteriorly on the suture between the angular and prearticular.

#### Surangular

The surangular forms most of the posterolateral part of the ramus and the lateral wall of the fossa meckelii (Fig 8). The surangular contacts the coronoid anterodorsally, the dentary anteroventrally, the angular posteroventrally, and the articular posteriorly and posteromedially. A single foramen nervi auriculotemporalis opens on its lateral surface. The posterodorsal part of the surangular forms the lateral part of the area articularis mandibularis. There, a furrow on the surangular-articular suture matches a low parasagittal ridge on the condylus mandibularis of the quadrate. The part of the area articularis mandibularis that lies on the surangular is slightly convex.

#### Coronoid

The coronoid is a triangular element that contacts the dentary anterolaterally, the surangular posterolaterally, the prearticular posteroventrally, and the splenial anteroventrally (Fig 8). The processus coronoideus is well developed. In lateral view, the coronoid is only exposed at the tip of the processus coronoideus. The coronoid forms the anteromedial margin of the dorsal opening of the fossa meckelii and also enters the dorsal margin of the foramen intermandibularis medius. Anteromedially, the coronoid extends below the lingual ridge of the dentary, but does not take part in the triturating surface. A foramen (two on the left coronoid) is present on the anteroventral part of the bone. Parsons and Williams [18] described a similar ventrolaterally-leading foramen in *Portlandemys mcdowelli*. Gaffney [20] referred to this foramen as the foramen alveolare inferius in his figures, but based on available definitions [40,41] this is incorrect as the foramen alveolare inferius is an opening in the dentary that leads into the canalis alveolaris inferior. This foramen in the coronoid is therefore unnamed. A similar foramen also occurs in *Plesiochelys etalloni*, *Plesiochelys planiceps*, and *Solnhofia parsonsi*.

#### Articular

The articular forms a large part of the posterior tip of the ramus. It contacts the surangular dorsolaterally, the prearticular medially, and the angular ventrolaterally (Fig 8). The articular enters the posteriormost margin of the dorsal opening of the fossa meckelii and bears most of the area articularis mandibularis. As noted above, the articular and surangular define a furrow along their common suture dividing the area articularis mandibularis into two parts. The medial part on the articular is moderately concave, whereas the lateral part on the surangular is slightly convex. The posterior third of the articular forms a well developed, posteromedial retroarticular process. The dorsal surface of the retroarticular process is concave and bears a well-defined foramen posterius chorda tympani.

#### Prearticular

The prearticular is a long, plate-like element that forms most of the posterior part of the medial surface of the ramus. It contacts the coronoid anterodorsally, the splenial anteroventrally, the angular ventrally, and the articular posteriorly (Fig 8). The prearticular forms most of the medial wall of the fossa meckelii. About halfway along the ventral suture with the angular, the prearticular forms the medial half of the foramen intermandibularis caudalis. In contrast to *Plesiochelys etalloni*, *Plesiochelys planiceps*, and *Solnhofia parsonsi*, the prearticular does not bear the anteromedial part of the area articularis mandibularis. The condition in *Portlandemys mcdowelli* is unknown.

## Discussion

### Note on *Plesiochelys planiceps*

*Plesiochelys planiceps* is based on a single specimen (OUMNH J.1582) from the Portland Beds (Tithonian) of the Isle of Portland, UK. The material, which consists of a cranium, a mandible, and remains of the hyoids and cervical vertebrae, was initially briefly described by Owen [27] as *Chelone planiceps*. The material was prepared using acetic acid and air abrasives at the AMNH in 1972–73. This species was later referred to *Plesiochelys* by Gaffney [19] and differentiated from *Plesiochelys etalloni* mainly based on characters pertaining to the lower jaw. The cranial anatomy of *Plesiochelys* was discussed by Gaffney [20], but only minor references were made to *Plesiochelys planiceps*. Consequently, no detailed description of the anatomy of *Plesiochelys planiceps* yet exists in the literature.

In contrast to Gaffney [20], our observations indicate that *Plesiochelys planiceps* differs from *Plesiochelys etalloni* in many aspects of the cranium: e.g., elongate foramen palatinum posterius; absence of ossification of the dorsal part of the pila prootica; absence of contact between the parietal and the quadrate posterior to the foramen nervi trigemini; strongly developed processus trochlearis oticum; foramen posterius canalis carotici interni not located on the ventral surface of the basicranium; canalis caroticus internus located deep within bone; each exoccipital forming one third of the condylus occipitalis. Several of these aspects will be discussed below. Furthermore, the specimen is larger than any referred to *Plesiochelys etalloni* (Table 1), yet many sutures of the cranium and mandible are still open, which indicates that the individual had not reached the adult stage at the time of death. These observations strongly support *Plesiochelys planiceps* as a distinct taxon.

### Note on *Portlandemys mcdowelli*

*Portlandemys mcdowelli* is based on three partial crania and one incomplete mandible from the Portland Beds (Tithonian) of the Isle of Portland, UK. The material arrived in the collection of the NHMUK in 1899 (by exchange with R. Damon) and was acid prepared by A. E. Rixon in 1953. Parsons and Williams [18] provided a detailed description of this material, but did not attempt to assign it a name. Gaffney [19] recognized it as a new species, *Portlandemys mcdowelli*, then compared it notably with *Plesiochelys etalloni* [20]. The holotype, NHMUK R2914, is a fairly complete skull associated with a partial mandible. The cranium NMHUK R3164 belongs to a smaller individual and is less complete than the holotype. NHMUK R3163 is the less complete of the specimens initially referred to *Portlandemys mcdowelli*. It consists of an eroded braincase fragment associated, but not articulated, with a portion of skull roof (parietals, frontals, and right postorbital), as well as the palatines, the left maxilla, and the vomer. In April 1985, R. Hirayama visited the NHMUK and left a note with specimen NMHUK R3163 expressing his doubt as to the identification of this material. He notably noted the presence of a large foramen palatinum posterius and the absence of contact between the maxilla and the pterygoid. These features are known in *Plesiochelys etalloni*, *Plesiochelys planiceps*, and '*Thalassemys*' *moseri*, but not in the holotype of *Portlandemys mcdowelli*. We confirm these observations and further add that the configuration of the dorsum sellae and sella turcica in NHMUK R3163 is different from that characterizing NHMUK R2914 and NHMUK R3164. The surface below the dorsum sellae is almost vertical dorsally before sloping anteroventrally. As a result, the dorsum sellae does not overhang the sella turcica, but the foramina anterius canalis carotici cerebralis open only a short distance in front of the dorsum sellae. Such a configuration is found in *Plesiochelys etalloni* and *Plesiochelys planiceps*. We therefore exclude NHMUK R3163 from *Portlandemys mcdowelli*. Based on the absence of complete ossification of the pila prootica and the well-developed processus trochlearis oticum, it might represent a new specimen of *Plesiochelys planiceps*, but the poor preservation prevents a definitive conclusion.

As noted above, NHMUK R3164 is a juvenile individual, which gives us some insights into the ontogenetic development of *Portlandemys mcdowelli*. These insights are important with respect to the description of *Portlandemys gracilis*. For example, the processus trochlearis oticum is strong in NHMUK R3164, and the configuration of the dorsum sellae and sella turcica is similar to that of NHMUK R2914. In contrast, NHMUK R3164 is less strongly ossified and proportionally less robust than NHMUK R2914. For example, the interorbital region of the skull roof is thinner, the triturating surface of the upper jaw is more gracile, and the structures in the ventral surface of the basicranium are less pronounced. These characteristics must have appeared late in the ontogeny of *Portlandemys mcdowelli*. Since the material of *Portlandemys gracilis* described herein is within the same size range as NHMUK R2914, it is reasonable to assume that the more gracile features of our new species are independent of ontogeny.

Under the same catalogue number as the holotype of *Portlandemys mcdowelli* (NHMUK R2914), the NHMUK also houses several postcranial elements: a costal plate, a fragment of carapace rim, a right humerus, and a complete coracoid. Based on available information, these are also from the Portland Beds of the Isle of Portland and were also exchanged with R. Damon in 1899. Whether they belong to the same individual as the cranium and mandible should however be investigated.

### Foramen nervi trigemini

The area of the foramen nervi trigemini is highly characteristic in plesiochelyids and '*Thalassemys*' *moseri*. The anterior part of the braincase wall formed mostly by the processus inferior parietalis is shorter than in most turtles due to the great development of the foramen interorbitale. The processus inferior parietalis also extends further ventrally forming most of the anterior and posterior margins of the foramen nervi trigemini. Anteriorly, the parietal meets the pterygoid about halfway up the foramen nervi trigemini, hence excluding the epipterygoid from the margin of that foramen, both on the lateral and medial surface of the braincase wall (Fig 4E–4H). Posteriorly, the parietal covers the prootic anterolaterally, excluding this element from the posterior margin of the foramen nervi trigemini, and usually reaches the quadrate posteroventral to that foramen.

This typical morphology is found in *Plesiochelys etalloni*, *Portlandemys mcdowelli*, *Portlandemys gracilis*, and '*Thalassemys*' *moseri*. Gaffney [20] argued that the epipterygoid enters the anterior margin of the foramen nervi trigemini in NMS 8738, one of the Solothurn skulls referred to *Plesiochelys etalloni*. Close examination reveals that the posterodorsal corner of the epipterygoid indeed comes very close to the anteroventral margin of the foramen nervi trigemini, but that a thin anterodorsal process from the pterygoid still reaches the parietal and excludes the epipterygoid from the foramen margin. This is also the morphology observed in NMS 40870 and NMS 40871 (see above). In other specimens referred to *Plesiochelys etalloni*, the pterygoid-parietal contact excluding the epipterygoid from the margin of the foramen nervi trigemini is broader.

The morphology in *Plesiochelys planiceps* (OUMNH J.1582) differs in some aspects from that of other plesiochelyids and '*Thalassemys*' *moseri*. First, the part of the braincase wall anterior to the foramen nervi trigemini is shorter in *Plesiochelys planiceps* than in other plesiochelyids. As a result, the epipterygoid is notably shorter anteroposteriorly in this species. Posterior to the foramen nervi trigemini, a parietal-pterygoid contact still prevents the prootic from entering the margin of the foramen nervi trigemini in lateral view, but the posteroventral extension of the parietal is considerably reduced compared to other plesiochelyids and the parietal does not reach the quadrate.

In contrast to other interpretations [18,53], we think that the parietal of *Solnhofia parsonsi* also develops a broad posteroventral process that meets the pterygoid and quadrate, excluding the prootic from the posterior margin of the foramen nervi trigemini in lateral view. Our conclusions are based on the study of the skull NMS 8741 from Solothurn and of a yet undescribed skull from the Kimmeridgian of Porrentruy (MJSN BAN001-2). They are also confirmed by photographs of the nicely preserved holotype skull (TM 4023) of this species.

It should also be noted that the xinjiangchelyid *Annemys levensis* Sukhanov and Narmandakh, 2006 [57] exhibits a somewhat similar condition. In this species, the parietal sends a long posteroventral process that forms a large part of the posterodorsal margin of the foramen nervi trigemini [58]. However, in contrast to the taxa discussed herein, this process does not reach either the pterygoid or the quadrate, and the prootic does enter the posterodorsal margin of the foramen nervi trigemini, albeit for a short length. Other xinjiangchelyids in which this area is known do not seem to possess this condition.

### Dorsum sellae and sella turcica

In *Plesiochelys etalloni*, *Plesiochelys planiceps*, *Portlandemys mcdowelli*, and *Portlandemys gracilis*, the dorsum sellae is high and does not overhang the posterior part of the sella turcica. As a result, the foramina anterius canalis carotici cerebralis open anterior to the dorsum sellae instead of underneath it, and the surface below the dorsum sellae is well developed and extends anteroventrally instead of posteroventrally (Fig 4). As noted by Gaffney [20], this peculiar arrangement of the dorsum sellae and sella turcica is also known in Pan-Chelonioidea. However, all recent phylogenetic analyses (e.g., [8–12,59,60]) do not consider plesiochelyids as closely related to pan-chelonioids and this condition probably evolved convergently in the two groups. It is possible that this condition is linked to the adaptation to marine environments, like the large foramina interorbitale accommodating the hypertrophied salt glands in these two groups [7]. Interestingly, '*Thalassemys*' *moseri* and *Solnhofia parsonsi* lack this unique configuration of the dorsum sellae and sella turcica. This is therefore an important character uniting *Plesiochelys etalloni*, *Plesiochelys planiceps*, *Portlandemys mcdowelli*, and *Portlandemys gracilis* relatively to other Late Jurassic coastal marine turtles. However, the dorsum sellae is relatively high in '*Thalassemys*' *moseri*. With a high dorsum sellae that does not overhang the sella turcica, '*Thalassemys*' *moseri* might represent an intermediate state toward the plesiochelyid condition.

As described above, the condition in *Portlandemys mcdowelli* and *Portlandemys gracilis* is more developed than in *Plesiochelys etalloni* and *Plesiochelys planiceps*. The foramina anterius canalis carotici cerebralis open more anteriorly in the *Portlandemys* species. In fact, the cerebral branch of the internal carotid artery exits the basisphenoid about halfway between the dorsum sellae and the tip of the trabeculae in these forms (Fig 6A and 6B). Additionally, the surface below the dorsum sellae slopes more gently anteroventrally and reaches farther anteriorly, therefore appearing longer in dorsal view. These characteristics are then important for differentiating *Portlandemys mcdowelli* and *Portlandemys gracilis* from other plesiochelyids, and are one of the main reasons why we unite these two species within the same genus.

A sagittal ridge is usually present on the surface below the dorsum sellae, but its development is apparently subject to individual variation, as discussed by Gaffney [20]. In *Plesiochelys etalloni*, a ridge is present in most specimens (NMB 435, NMS 8738, and NMS 40870), but it is bifurcated ventrally in NMS 8740 and relatively reduced in NMS 40871. The ridge is absent in NMS 8739, whereas the condition in NMS 9145 remains conjectural. A sagittal ridge was probably also present in *Plesiochelys planiceps*, although the posterodorsal half of that ridge and the medial part of the dorsum sellae were apparently cartilaginous in that specimen. In *Portlandemys mcdowelli* (NHMUK R2914 and NHMUK R3164), a ridge is also present and it is more pronounced anteriorly than posteriorly. The ridge is acute and well-developed in *Portlandemys gracilis* (see above). This ridge and the anteriorly-sloping surface below the dorsum sellae are absent in '*Thalassemys*' *moseri* and *Solnhofia parsonsi*.

Another aspect of interest in this region is the relative closeness of the foramina anterius canalis carotici cerebralis in the posterior part of the sella turcica. In plesiochelyids (except NMS 8739, referred to *Plesiochelys etalloni*), the foramina anterius canalis carotici cerebralis lie close to one another. The bar of bone that separates the foramina is about the size of one of these foramina. In most other turtles, including '*Thalassemys*' *moseri* and NMS 8739, the foramina anterius canalis carotici cerebralis open in the posterolateral corner of the sella turcica and are therefore more widely separated. Of course, there are exceptions. The condition in cheloniids is greatly modified with the internal carotid running in the sulcus cavernosus and the cerebral branch entering the sella turcica laterally (see [20,41]). The condition in the eurysternid *Solnhofia parsonsi* is also interesting since the foramina anterius canalis carotici cerebralis are joined into a common opening at the back of the sella turcica and below the dorsum sellae [53].

### Anterior foramen nervi abducentis

According to Gaffney [20], the anterior foramen nervi abducentis opens ventral and posterior to the base of the processus clinoideus close to the suture with the pterygoid and almost in the floor of the sulcus cavernosus in *Plesiochelys* and *Portlandemys*. In most other turtles, the anterior foramen nervi abducentis is described as opening more anteriorly and dorsally. However, our observations are in partial contradiction with these propositions. It is correct that the anterior foramen nervi abducentis is mostly ventral and slightly posterior to the base of the processus clinoideus in *Plesiochelys etalloni* (Fig 4I and 4J). In other plesiochelyids and '*Thalassemys*' *moseri*, the anterior foramen nervi abducentis is better described as being moderately anteromedial to the base of the processus clinoideus, usually at the level or slightly behind the level of the dorsum sellae (Fig 6A and 6B). In all the turtles considered in the present study, the anterior foramen nervi abducentis opens relatively close to the basisphenoid-pterygoid suture. Therefore, to the possible exception of *Plesiochelys etalloni*, the position of the anterior foramen nervi abducentis is of poor systematic value.

### Foramen anterius canalis carotici palatinum and foramen nervi vidiani

The position of the foramen anterius canalis carotici palatinum and foramen nervi vidiani was extensively discussed by Gaffney [20] and proposed to be more advanced in *Plesiochelys etalloni* (foramen anterius canalis carotici palatinum opening more posteriorly and foramen nervi vidiani opening within the cavum epiptericum) than in *Plesiochelys planiceps* and *Portlandemys mcdowelli* (foramen anterius canalis carotici palatinum close to the anterior tip of the trabecula and foramen nervi vidiani outside the cavum epiptericum). Based on a reassessment of the specimens, we think that the situation is more complex (see below). The foramen anterius canalis carotici palatinum usually opens in the floor of the sulcus cavernosus between the trabecula medially and the crista pterygoidea laterally. The relative position of the foramen anterius canalis carotici palatinum is subject to interspecific and intraspecific variations, as already noted by Gaffney [20]. The architecture of the vidian canals, which carry the various portions of the palatine (vidian) branch of the facial (VII) nerve and several small arteries, is poorly documented in modern turtles and mostly unknown in fossil turtles [41]. The facial (VII) nerve emerges from the vestibular ganglion in the fossa acustico-facialis and penetrates the prootic. In cryptodires, the nerve travels laterally through the prootic and joins the canalis cavernosus, where it forms the geniculate ganglion. The palatine (vidian) branch emerges from the facial (VII) nerve at the geniculate ganglion and travels through the foramen pro ramo nervi vidiani (usually a short canal) to join the canalis caroticus internus. From there, the palatine (vidian) branch apparently travels for a time along one or several branches of the internal carotid artery before entering its own canal, the canalis nervi vidiani. This part is poorly documented in fossil turtles. The canalis nervi vidiani extends anteriorly and usually emerges in the dorsal surface of the pterygoid or palatine, forming the foramen (or foramina) nervi vidiani. However, this foramen is not always easy to identify in fossil turtles and can be easily confounded with other small openings that sometimes occur in this area.

In *Plesiochelys etalloni*, the foramen anterius canalis carotici palatinum opens slightly anterior to the level of the foramen anterius canalis carotici cerebralis (NMS 8739, NMS 8740, NMS 9145, NMS 40871; Fig 4A and 4B), or more anteriorly along the trabecula (NMB 435, NMS 8738, NMS 40870). The position of the foramen nervi vidiani is relatively variable in *Plesiochelys etalloni*. It may open slightly within the cavum epiptericum in front of the foramen anterius canalis carotici palatinum (NMS 8738), or about midway along the trabecula (left side of NMS 40871), or anteriorly a few millimeters behind the anterior tip of the trabecula (left side of NMS 8739, NMS 8740, left side of NMS 9145), or posteriorly within the still roofed canalis caroticus palatinum (right side of NMS 40871). The groove formed by the unroofed canalis caroticus palatinum is either open anteriorly (NMB 435, NMS 8738, right side of NMS 8739, NMS 8740, NMS 9145, NMS 40871), or closed by an anteromedial extension of the crista pterygoidea meeting the raised midline shelf of the pterygoid (left side of NMS 8739, NMS 40870). When the groove is barred anteriorly, a short canal within the pterygoid connects it to the outside of the braincase and opens anterior to the base of the epipterygoid.

In *Plesiochelys planiceps* (OUMNH J.1582), there is no proper opening of the canalis caroticus palatinum in the sulcus cavernosus, only a slit-like opening close to the anterior tip of the trabecula. The crista pterygoidea extends anteromedially and meets the midline shelf of the pterygoid. A short canal connects the canalis caroticus palatinum with the outside of the braincase through this ridge and exits anteromedial to the base of the epipterygoid (this large opening was interpreted as the foramen nervi vidiani by Gaffney [20]).

In *Portlandemys mcdowelli*, the foramen anterius canalis carotici palatinum opens close to the anterior tip of the trabecula in NHMUK R2914, but at the level of the foramen anterius canalis carotici cerebralis in NHMUK R3164. In NHMUK R2914 and NMHUK R3164, no sign of a potential foramen nervi vidiani is visible. The skull roof does not allow to study the sulcus cavernosus in details in any of the specimens. The crista pterygoidea forms an anteromedial ridge that meets the midline shelf of the pterygoid, but this ridge is not pierced by a short canal. In NHMUK R2914, moderately-sized, paired foramina open relatively high on the anterolateral surface of the triangular midline shelf of the pterygoid. In NHMUK R3164, the concave surface below the ridge is pierced by several moderately-sized foramina, some of them appearing to be paired. A fissure-like opening also occurs anterolaterally along the epipterygoid-pterygoid suture and was interpreted by Gaffney [20] as the foramen nervi vidiani. It is probable that at least some of the aforementioned foramina are part of the palatine artery or vidian nerve system, although we are unsure which one.

In *Portlandemys gracilis* (see description above), the foramen anterius canalis carotici palatinum opens approximately at the level of the foramen anterius canalis carotici cerebralis, which occurs in a relatively anterior position due to the configuration of the dorsum sellae and sella turcica in this taxon. Anterior to that foramen, the palatine branch of the internal carotid artery runs in a groove in the floor of the sulcus cavernosus. The foramen nervi vidiani opens in the lateral wall of that groove a few millimeters in front of the foramen anterius canalis carotici palatinum. The groove in which runs the palatine branch of the internal carotid artery is not barred anteriorly.

Based on the above survey, we now have a better understanding of this region in plesiochelyids. The anterior part of the canalis caroticus palatinum runs underneath the sulcus cavernosus just lateral to the trabecula. The canalis caroticus palatinum converges progressively anterodorsally toward the sulcus cavernosus, so that the layer of bone between these two structures becomes thinner anteriorly and eventually disappears, marking the position of the foramen anterius canalis carotici palatinum. From that point, the canalis caroticus palatinum is no longer roofed and forms a groove in the floor of the sulcus cavernosus. The foramen anterius canalis carotici palatinum is relatively variable in position in all the species for which several skulls are available (*Plesiochelys etalloni* and *Portlandemys mcdowelli*). It usually occurs between the level of the foramen anterius canalis carotici cerebralis and the anterior tip of the trabecula. The foramen anterius canalis carotici palatinum can also occurs at different position on each side of the same specimen.

The groove formed by the canalis caroticus palatinum in the sulcus cavernosus is variable in length due to the variable position of the foramen anterius canalis carotici palatinum. In many specimens (see above), it is also barred anteriorly by an anteromedial ridge extending from the crista pterygoidea and meeting the midline shelf of the pterygoid. When this ridge is present, a short canal usually connects the canalis caroticus palatinum with the outside of the braincase and exits anterior to the base of the epipterygoid. This opening was interpreted as the foramen nervi vidiani in *Plesiochelys planiceps* by Gaffney [20]. Although it may carry the palatine (vidian) branch of the facial (VII) nerve, its size suggests that this canal probably also carries at least some part of the palatine branch of the internal carotid artery. We think that the proper foramen nervi vidiani (the anterior opening of the canalis nervi vidiani) is located more posteriorly either within this canal or within the canalis caroticus palatinum, as seen in *Plesiochelys etalloni* and *Portlandemys gracilis*. The condition in *Portlandemys mcdowelli* is more complex to interpret and will require further analysis.

With this detailed review, we hope to demonstrate that, as far as the plesiochelyids are concerned, the position of the foramen anterius canalis carotici palatinum is probably not a reliable source of information for systematic purposes and that the question of the foramen nervi vidiani needs to be further investigated with modern techniques (CT-scan).

### Pila prootica

In *Plesiochelys etalloni*, a flat process extends ventromedially from the anteromedial wall of the prootic and articulates ventrally with the processus clinoideus of the basisphenoid. NMS 8739 and NMS 40871 clearly show that this band-like process is formed entirely by the prootic (Fig 4G and 4H). Gaffney [20], who first described this structure in *Plesiochelys etalloni*, considered that it resulted from the complete ossification of the dorsal part of the pila prootica, which usually remains cartilaginous in the adult turtle skull. *Plesiochelys etalloni* is unique among Mesozoic turtles in presenting a completely ossified pila prootica. In NMB 435 (a juvenile individual), the pila prootica is apparently not yet fully ossified, but a ventromedial process is present on the anteromedial surface of the prootic. Gaffney [20] suggested that *Plesiochelys planiceps* also had a completely ossified pila prootica, but that it was reduced in OUMNH J.1582 due to postmortem damage. Our observations contradict this proposition since there is clearly no flat process on the anteromedial surface of the prootic in this specimen. This character is therefore of major importance to diagnose *Plesiochelys etalloni*. Some specimens of the recent chelid *Emydura* Bonaparte, 1836 [61] also present a completely ossified pila prootica [41].

### Pterygoid fossa

The pterygoid fossa, or fossa pterygoidei, or fossa pterygoidea, or podocnemidid fossa, is a depression on the posteroventral surface of the pterygoid. This fossa probably accommodates some parts of the jaw adductor musculature, notably the M. adductor mandibulae internus Pars pterygoideus ventralis [56,62,63]. Besides plesiochelyids and eurysternids, this structure is present in a wide range of taxa, notably, but not restricted to, Xinjiangchelyidae Nessov in Kaznyshkin et al., 1990 [64], Baenidae Cope, 1882 [65], Solemydidae Lapparent de Broin and Murelaga, 1996 [66], and Bothremydidae Baur, 1891 [67] (e.g., [13,68–74]).

In plesiochelyids and eurysternids, the pterygoid fossa is usually triangular in outline in ventral view and is formed only by the pterygoid. It is bordered laterally by the ventral edge of the quadrate process of the pterygoid, and medially by a more or less pronounced sub-parasagittal ridge extending on the medial part of the ventral surface of the pterygoid. Posteriorly, it terminates at the posterior margin of the pterygoid. The development of the pterygoid fossa is variable. The pterygoid fossa is well-developed in *Plesiochelys etalloni* (more so in NMS 40870) and *Portlandemys gracilis*. However, it is proportionally deeper in *Portlandemys mcdowelli*, *Plesiochelys planiceps*, and '*Thalassemys*' *moseri*. *Portlandemys mcdowelli* is also remarkable because the pterygoid fossa is bordered posteriorly by a ridge, whereas in other species the posterior margin of the pterygoid simply curves dorsally. The pterygoid fossa is also relatively well-developed in the eurysternids *Parachelys eichstaettensis* (NHMUK 42888; JA, unpublish. data) and *Solnhofia parsonsi*, although it is posteriorly pinched in the latter species.

### Canalis caroticus internus

How the internal carotid artery penetrates the basicranium is usually regarded as a fundamental feature for turtle systematics (e.g., [41,75]). However, many studies in recent years have revealed that the picture is more complex than previously depicted (e.g., [13,21,76,77]). Gaffney [20] initially described the plesiochelyids as displaying the typical eucryptodiran condition with a canalis caroticus internus floored posteriorly up to the posterior part of the pterygoid. The flooring of the canalis caroticus internus was described as thin in all plesiochelyids, and the only difference between *Plesiochelys* and *Portlandemys* was considered to be the formation of the foramen posterius canalis carotici interni by the basisphenoid and pterygoid in the former, and by the pterygoid only in the latter.

Among the crania referred to *Plesiochelys etalloni*, only one (NMB 435) exhibits a completely floored canalis caroticus internus. Gaffney [20] interpreted all of the other crania as being in various states of deterioration regarding this feature. Rieppel [39] seriously questioned this interpretation. Considerations regarding the plesiomorphic condition of eucryptodires aside, the description of NMS 40870 and NMS 40871 presented herein confirms the interpretation of Rieppel [39]. NMS 40870 is the only known specimen referred to *Plesiochelys etalloni* in which this area is preserved in pristine state. The posterior half of the canalis caroticus internus is floored by a thin ventromedial flap of the pterygoid. The anterior half of the canalis caroticus internus is superficial and open ventrally up to the point where the cerebral and palatine branches of the internal carotid artery split and enter there own canals. We believe this is the most common condition in *Plesiochelys etalloni* since it is observed in most specimens. Based on the available specimens, Gaffney [20] also described the foramen posterius canalis carotici interni as being formed by the basisphenoid and pterygoid in *Plesiochelys etalloni*. However, NMS 40870 reveals that the foramen posterius canalis carotici interni is actually formed only by the pterygoid.

The canalis caroticus internus of *Plesiochelys planiceps* is not discussed in details in Gaffney [20]. Based on the published reconstruction of the holotype OUMNH J.1582 ([19], fig 15), the canalis caroticus internus is completely floored up to the posterior margin of the pterygoid. The foramen posterius canalis carotici interni is not visible in this reconstruction. Examination of the specimen reveals that the basisphenoid-pterygoid suture is somewhat damaged. The suture is slightly open on the left hand side, and partly broken on the right. This may give the misleading impression that the canalis caroticus internus is at least partly open ventrally in this specimen. However, close observation reveals that there is no trace of the canalis caroticus internus in the open sutures between the basisphenoid and the pterygoids. The canalis caroticus internus is actually located deeper within the bone, which contrasts with the condition in *Plesiochelys etalloni* where the canalis is more superficial. Furthermore, the foramen posterius canalis carotici interni does not open on the ventral surface of the basicranium. It is formed entirely by the pterygoid and is located on the posteromedial margin of the bone close to the basisphenoid. This is another difference between *Plesiochelys planiceps* and other plesiochelyids.

In *Portlandemys mcdowelli*, the foramen posterius canalis carotici interni is formed entirely by a fold of the pterygoid and located posteromedially on the ventral surface of this bone. In NHMUK R2914, the canalis caroticus internus is entirely floored up to the foramen posterius canalis carotici interni. This condition is superficially similar to that of *Plesiochelys etalloni* where a ridged ventromedial flap of the pterygoid encloses the internal carotid artery. However, this ridged flap of the pterygoid is broken on both sides of NHMUK R3164 and reveals that the canalis caroticus internus is not as shallow as in *Plesiochelys etalloni*, but occurs more deeply within the pterygoid and basisphenoid as in *Plesiochelys planiceps*. In other words, the ridged ventromedial flap of the pterygoid does not floor the canalis caroticus internus in *Portlandemys mcdowelli*.

In *Portlandemys gracilis*, the foramen posterius canalis carotici interni is formed entirely by the pterygoid and lies within the posterior margin of the bone relatively far from the basisphenoid-pterygoid suture. It is barely visible in ventral view, which contrasts with the condition in *Portlandemys mcdowelli*. The only known cranium (MJSN BSY009-708) reveals very little information regarding the canalis caroticus internus. From the foramen posterius canalis carotici interni, the canalis caroticus internus curves anteromedially and appears to be set relatively deep within the pterygoid bone.

Among plesiochelyids, *Plesiochelys etalloni* is therefore the only taxon in which the canalis caroticus internus is superficial and usually only partly floored by the pterygoid. It should be noted that this condition is similar to that of '*Thalassemys*' *moseri* and is probably plesiomorphic for the group (e.g., [77]).

### Infolding ridge on quadrate

Gaffney [20] was the first to note the presence of a prominent, ventrally-infolding ridge on the posterior surface of the quadrate below the incisura columellae auris in *Plesiochelys* and *Portlandemys*. He suggested that this ridge may serve for the attachment of the M. depressor mandibulae or M. pterygoideus portio ventralis (M. adductor mandibulae internus Pars pterygoideus ventralis of Werneburg [56]). However, the M. depressor mandibulae seldom originates from the quadrate, and when it does it is usually from the posterior process of the quadrate, posterior to the incisura columellae auris [56,78]. The M. adductor mandibulae internus Pars pterygoideus ventralis usually originates mostly from the ventral surface of the pterygoid, but it does extends to the posterior surface of the mandibular process of the quadrate in some turtles, notably in cheloniids [78,79]. It is therefore plausible that this ridge served for the attachment of the Pars pterygoideus ventralis. The fact that this ridge and the depression that it forms on the posterior surface of the mandibular process of the quadrate are continuous medially with the well-developed pterygoid fossa found in these taxa (see above) is also consistent with this interpretation.

Rugosities are relatively common in this area and might be also linked to the attachment of the eustachian tube [41], but the ridge developed in *Plesiochelys* and *Portlandemys* is a unique structure in turtles. This ridge is present in *Plesiochelys etalloni*, *Plesiochelys planiceps*, *Portlandemys mcdowelli*, and *Portlandemys gracilis*, but also in '*Thalassemys*' *moseri*, and in the eurysternids *Solnhofia parsonsi* [53] and *Parachelys eichstaettensis* (NHMUK 42888; JA, unpublish. data). Like the presence of three cervical scales on the carapace [80], this feature strongly suggests that these Late Jurassic coastal marine turtles are probably closely related.

### Condylus mandibularis

The condylus mandibularis of the quadrate is rarely described in details, although it can be relatively different from one species to another. For example, the lateral lobe of the condylus is much larger than the medial one in the eurysternid *Solnhofia parsonsi* [53]. In *Plesiochelys etalloni*, the lateral and medial margins of the condylus mandibularis dip moderately ventrally, but the rest of the structure is mostly flat. In *Plesiochelys planiceps*, the two facets are well developed and concave, and they are separated by a wide, well-developed parasagittal furrow. In *Portlandemys gracilis*, the two facets are also well developed and concave, but they are separated by a low parasagittal ridge (see above). Additionally, the lateral margin of the condylus appears to dip more strongly ventrally than the medial one in this taxon. The morphology of the condylus mandibularis may not hold significant phylogenetic information at that taxonomic scale, but it may at least help with the identification of species. Unfortunately, the condition in *Portlandemys mcdowelli* and '*Thalassemys*' *moseri* is unknown at the moment.

## Phylogenetic Analysis

### New cranial characters

The five following cranial characters have been added to the original character-taxon matrix of Joyce [10] in an attempt to evaluate their impact on the relationships of plesiochelyid turtles. A complete list of characters is provided as supporting information (S1 File).

#### Character 13 (Parietal D)

Parietal sending a posteroventral process forming most of the posterior margin of the foramen nervi trigemini; this process contacts the pterygoid ventrally and excludes the prootic from the margin of the foramen nervi trigemini in lateral view: absent (0); present (1).

This character is present in all scored plesiochelyids (skull not known in *Tropidemys langii*), as well as in '*Thalassemys*' *moseri*, *Solnhofia parsonsi*, and *Ordosemys leios* Brinkman and Peng, 1993 [81] [82]. As noted above (see Foramen Nervi Trigemini), *Plesiochelys planiceps* differs from other plesiochelyids in having a more reduced posteroventral process of the parietal that still excludes the prootic from the posterior margin of the foramen nervi trigemini, but does not reach the quadrate ventrally. However, this difference is irrelevant giving the present character wording.

#### Character 37 (Quadrate G)

Infolding ridge on the posterior surface of the quadrate below the incisura columellae auris: absent (0); present (1).

This character is present in all scored plesiochelyids (skull not known in *Tropidemys langii*), as well as in '*Thalassemys*' *moseri* and the eurysternid *Solnhofia parsonsi* (see Infolding Ridge on Quadrate).

#### Character 39 (Epipterygoid B)

Epipterygoid excluded from the margin of the foramen nervi trigemini by a contact between the parietal and pterygoid: absent (0); present (1).

This character is present in all scored plesiochelyids (skull not known in *Tropidemys langii*), as well as in '*Thalassemys*' *moseri*. The contribution of the epipterygoid to the anterior margin of the foramen nervi trigemini is variable in extant cryptodires and is absent in certain taxa [41]. Unfortunately, we lacked comparative material to score this character in many taxa.

#### Character 54 (Basisphenoid C)

Position of the sella turcica relative to the dorsum sellae: dorsum sellae overhangs the sella turcica posteriorly (0); dorsum sellae does not overhang the sella turcica, development of an anteriorly sloping surface below the dorsum sellae (1).

This character is present in all scored plesiochelyids (skull not known in *Tropidemys langii*), as well as in chelonioids, *Toxochelys latiremis* Cope, 1873 [52,83], and *Sandownia harrisi* Meylan et al., 2000 [84]. Interestingly, it is absent in '*Thalassemys*' *moseri* and *Solnhofia parsonsi* (see Dorsum Sellae and Sella Turcica).

#### Character 55 (Basisphenoid D)

Complete ossification of the pila prootica (sheet of bone connecting the processus clinoideus of the basisphenoid to the prootic): absent (0); present (1).

As mentioned above (see Pila Prootica), the complete ossification of the pila prootica is a unique characteristic of *Plesiochelys etalloni*. None of the currently-known plesiochelyids or closely-related taxa exhibit this condition. In the context of the present study, this is therefore an autapomorphy of *Plesiochelys etalloni*. However, it should be noted that a completely ossified pila prootica is also known in the basal turtles *Proganochelys quenstedti* Baur, 1887 [85] and *Palaeochersis talampayensis* Rougier at al., 1995 [86] [87,88], and may therefore represents the plesiomorphic condition for Testudinata Klein, 1760 [89] (sensu [4]).

### Results

**Fig 9:**
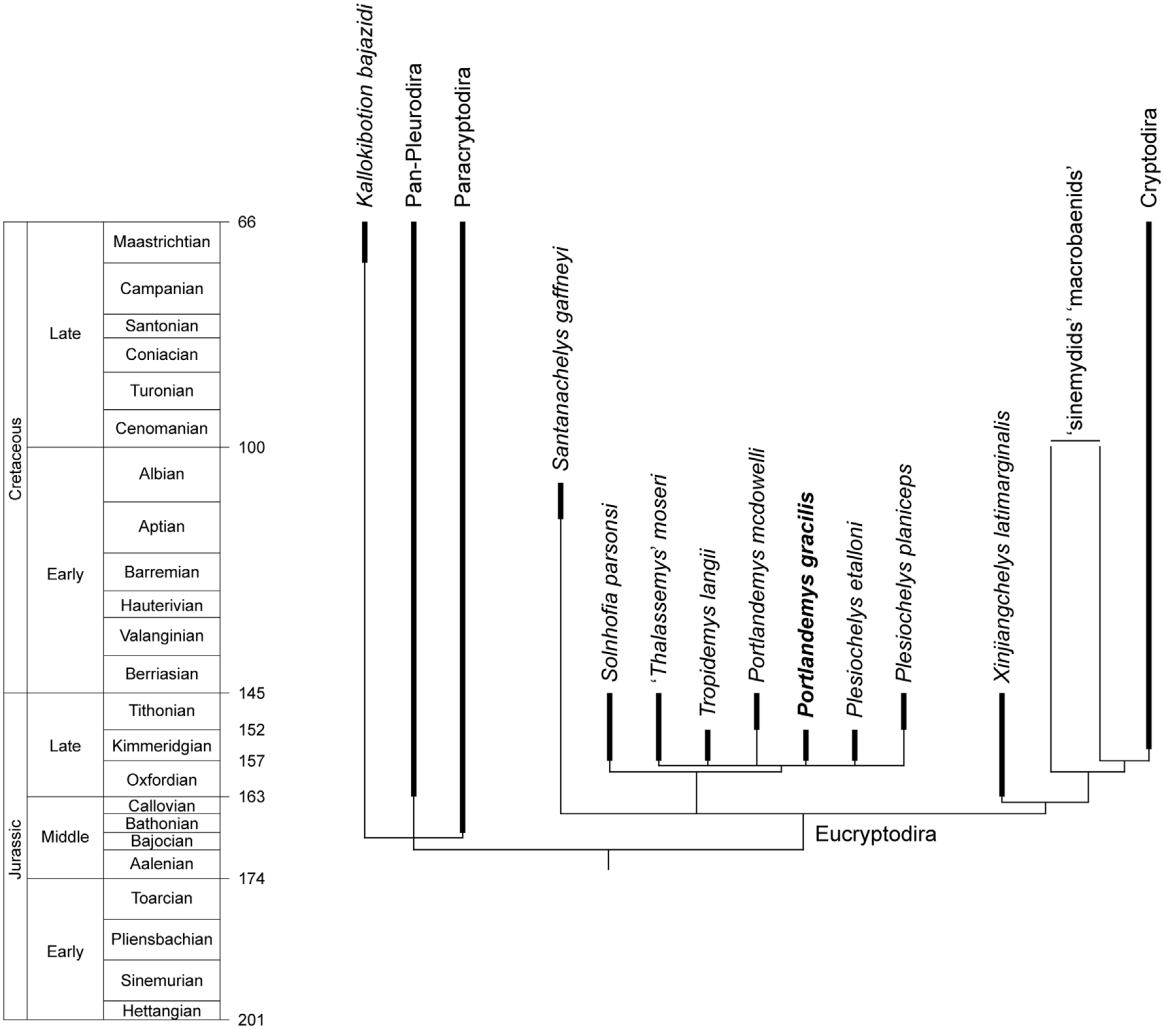
Phylogenetic relationships of plesiochelyid turtles. Partial version of the strict consensus of 180 Wagner trees (see S1 File for complete tree).

The primary parsimony analysis (see Material and Methods) resulted in 180 most parsimonious trees of 376 steps (CI = 0.463; RI = 0.813). The strict consensus tree (393 steps; CI = 0.443; RI = 0.797) is well resolved, but relatively poorly supported with most nodes having low Bremer values. Detailed results (strict consensus tree, list of synapomorphies, Bremer support values) are provided as supporting information (S1 File). A condensed version of the strict consensus tree showing only that part of the tree that is concerned with plesiochelyid relationships is provided as Fig 9.

For comparative purposes, our primary analysis follows the design of the second analysis of Joyce [10]: all terminal taxa are included and 15 morphoclinic multistate characters are ordered (see Material and Methods). Our strict consensus tree is better resolved and somewhat comparable to the second and third Adams consensus trees of Joyce [10]. However, the newly added cranial characters help to fix the position of plesiochelyids and closely-related taxa, such as '*Thalassemys*' *moseri* and *Solnhofia parsonsi* (Fig 9). These taxa form a clade supported by two synapomorphies: the presence of a long posteroventral process of the parietal forming the posterior margin of the foramen nervi trigemini and excluding the prootic from that foramen (character 13, new, state 1); and the presence of a ventrally-infolding ridge on the posterior surface of the quadrate below the incisura columellae auris (character 37, new, state 1). The polytomy uniting the plesiochelyids and '*Thalassemys*' *moseri* is supported by seven synapomorphies: epipterygoid excluded from the anterior margin of the foramen nervi trigemini (character 39, new, state 1); foramen palatinum posterius present but open laterally (character 45, state 1); dorsum sellae does not overhang the sella turcica (character 54, new, state 1); more than one cervical scale present (character 74, state 2); bridge osseous (character 79, state 0); axillary buttress contacts peripherals and first costal (character 88, state 1); inguinal buttress contacts peripherals and fifth costal (character 90, state 1).

Like most phylogenetic analyses of turtles based on morphological characters, our study fails to recover a pattern of relationships within Cryptodira Cope, 1868 [90] that is congruent with molecular data [45,91–95]. Most notably, pan-chelonioids are resolved as the sister group to all remaining cryptodires, whereas trionychians are highly nested within cryptodires (S1 File). As a result, there has been an increasing trend toward the use of a molecular scaffold in order to constrain morphology-based phylogenetic analyses of turtles [13,58,96,97]. In order to test the effect of such a constraint on plesiochelyid relationships, we also ran the analysis forcing the relationships among living cryptodires based on the results of Crawford et al. [45]. As far as the plesiochelyids are concerned, the results of this constrained analysis are identical to those of the unconstrained analysis (see S7 File).

With the exception of Gaffney and Meylan [5], all published phylogenetic analyses that considered plesiochelyids up to 2007 (e.g., [6,8,9,59]) grouped them as a single terminal taxon, forfeiting the test of their monophyly. Gaffney and Meylan [5] found a 'plesiochelyid' clade formed by *Plesiochelys etalloni*, '*Thalassemys*' *moseri*, and *Portlandemys mcdowelli*, but *Solnhofia parsonsi* (representing the Eurysternidae) was not included in their analysis. *Solnhofia parsonsi* was however used as a terminal taxon by Gaffney et al. [9] and found as the sister-group of a terminal Plesiochelyidae. The analysis of Joyce [10] was the first to include *Plesiochelys etalloni*, *Portlandemys mcdowelli*, '*Thalassemys*' *moseri*, and *Solnhofia parsonsi* as terminal taxa, but found this assemblage to be paraphyletic. Although all global phylogenetic analyses of turtles since Joyce [10] included these taxa as terminals (e.g., [96,97][11–13,60]), none found them as forming a monophyletic group. The present analysis is therefore the first phylogenetic analysis to find that plesiochelyids, '*Thalassemys*' *moseri*, and *Solnhofia parsonsi* (representing the Eurysternidae) form a monophyletic group at the base of Eucryptodira, with '*Thalassemys*' *moseri* more closely related to plesiochelyids (Fig 9). Four out of the five newly proposed cranial characters support these relationships. This demonstrates the importance of these characters for phylogenetic reconstructions. However, the detailed relationships of plesiochelyids and '*Thalassemys*' *moseri* are not yet resolved and will require further investigations.

Similarly to most recent phylogenetic analyses, two taxa stand out as needing serious reconsideration. The first is *Santanachelys gaffneyi* Hirayama, 1998 [7], which was originally interpreted as the oldest Chelonioidea Baur, 1893 [98]. Since Joyce [10], *Santanachelys gaffneyi* was often found outside Chelonioidea and usually resolved as a basal Eucryptodira (e.g., [11,12,60]). This is also the case of the present study, both for the unconstrained and constrained analyses. A detailed redescription of this material and the inclusion of more Protostegidae Cope, 1872 [99] into phylogenetic analyses are needed. The second taxon that requires revision is *Sandownia harrisi* [84]. This species was initially interpreted as a Trionychia Hummel, 1929 [100] [84], but recent studies incorporating additional closely-related taxa found *Sandownia harrisi* either to be closely related to *Solnhofia parsonsi* [101], or as potentially related to chelonioids [102]. In the present study (unconstrained and constrained analyses), *Sandownia harrisi* is recovered as a stem chelonioid (see S1 File and S7 File).

## Conclusions

This study represents the second published work on the diverse turtle assemblage from the late Kimmeridgian of Porrentruy (see [30]). Herein, we describe a new plesiochelyid turtle from the late Kimmeridgian of Porrentruy: *Portlandemys gracilis*. The new species is notably distinguished from the type-species *Portlandemys mcdowelli* (Tithonian, southern England) by a more gracile morphology, the presence of a reduced and narrow processus trochlearis oticum, the absence of midline contact between the prefrontals, a pterygoid fossa not bordered posteriorly by a ridge, foramina posterius canalis carotici interni located more laterally and barely visible in ventral view, the presence of a raised pedestal where the pterygoid meets the processus interfenestralis, and a basisphenoid ogival in outline in ventral view. A mandible tentatively referred to *Portlandemys gracilis* also exhibits numerous differences with that of *Portlandemys mcdowelli* (reduced triturating surface, low profile, broadly rounded symphyseal region, splenial triangular in outline). However, *Portlandemys gracilis* and *Portlandemys mcdowelli* share a unique combination of features (narrower skull, labial ridges forming an acute angle, presence of an elongated surface below the dorsum sellae greatly offsetting the position of the foramina anterius canalis carotici cerebralis) that clearly suggests close relationship of these two species relative to other plesiochelyids. The new species from Porrentruy therefore extends the stratigraphical and paleobiogeographical distribution of the genus *Portlandemys*.

The description of this new material from Porrentruy was also the occasion of reassessing the cranial anatomy of plesiochelyid turtles. In this matter, we were helped by the study of new specimens of *Plesiochelys etalloni* from Solothurn that allowed to extend comparisons and complete previously published descriptions. In the second part of this paper, we provide a detailed discussion of several important cranial characters among plesiochelyids. Although these characters are useful to differentiate the different species at hand, most of them are probably poor phylogenetic characters. At this taxonomic scale, fine anatomical differences often result in contradictory phylogenetic signals. However, some of these characters (e.g., configuration of the dorsum sellae and sella turcica; contribution of the parietal to the margins of the foramen nervi trigemini; presence of an infolding ridge on the quadrate below the incisura columellae auris) have the potential to enlighten the phylogenetic relationships of plesiochelyids. Five of these characters were included in a previously published character-taxon matrix and, for the first time, provide support for a monophyletic group uniting plesiochelyids, '*Thalassemys*' *moseri*, and *Solnhofia parsonsi* (representing the Eurysternidae). This study is a first step toward a better understanding of the phylogenetic relationships of these Late Jurassic coastal marine turtles.

## Supporting Information

S1 File Supporting information. Including (I) Character list; (II) Data matrix; (III) Altered codings; (IV) TNT procedure; (V) Strict consensus tree with synapomorphies; (VI) Bremer supports; (VII) Literature cited.
(PDF)

S2 File Character-taxon matrix in TNT format. (ZIP)

S3 File Textured 3D model of cranium NMS 40870. High-resolution model available on figshare: http://dx.doi.org/10.6084/m9.figshare.1306563
(PDF)

S4 File Textured 3D model of basicranium NMS 40871. High-resolution model available on figshare: http://dx.doi.org/10.6084/m9.figshare.1306569
(PDF)

S5 File Textured 3D model of cranium MJSN BSY009-708. High-resolution model available on figshare: http://dx.doi.org/10.6084/m9.figshare.1306590
(PDF)

S6 File Textured 3D model of mandible MJSN SCR011-441. High-resolution model available on figshare: http://dx.doi.org/10.6084/m9.figshare.1306570
(PDF)

S7 File Phylogenetic analysis with molecular scaffold. Topology used to constrain relationships among living cryptodires and results of the constrained analysis.
(ZIP)
