# Supplementary material for: Portlandemys gracilis n. sp., a New Coastal Marine Turtle from the Late Jurassic of Porrentruy (Switzerland) and a Reconsideration of Plesiochelyid Cranial Anatomy
Source: PLoS One. 2015 Jun 24;10(6):e0129193. doi: 10.1371/journal.pone.0129193 (PMC4480851; doi:10.1371/journal.pone.0129193)
Supplement: S1 File — Including (I) Character list; (II) Data matrix; (III) Altered codings; (IV) TNT procedure; (V) Strict consensus tree with synapomorphies; (VI) Bremer supports; (VII) Literature cited. (PDF) [file pone.0129193.s001.pdf]

S1 File — Supporting Information

Portlandemys gracilis n. sp., a new coastal marine turtle from the Late Jurassic of Porrentruy (Switzerland) and a reconsideration of plesiochelyid cranial anatomy

Jérémy Anquetin*, Christian Püntener and Jean-Paul Billon-Bruyat

Section d'archéologie et paléontologie, Office de la culture, République et Canton du Jura, 2900 Porrentruy, Switzerland

*Corresponding author: j.anquetin@gmail.com

Contents.

I. Character list
II. Data matrix
III. Codings altered compared to the original matrix of Joyce (2007)
IV. Analysis with TNT
V. Strict consensus tree with listed synapomorphies
VI. Bremer supports
VII. Literature cited

I. Character list

The following character list is provided as a convenience to the reader. All of the characters but characters 13, 37, 39, 54, and 55 are from Joyce (2007), and the reader is referred to this publication for more information. Characters 13, 37, 39, 54, and 55 (in **bold** below) are new and are discussed in the main text of the present article. Character numbering follows conventions in TNT (i.e., starts at 0).

0. Nasal A — Nasals: present (0); absent (1).
1. Nasal B — Nasals contact one another medially along their entire length (0); medial contact of nasals partially or fully hindered by long anterior frontal process (1).
2. Nasal C — Dorsal exposure of nasals: large (0); greatly reduced relative to that of frontals (1).
3. Prefrontal A — Medial contact of prefrontals on dorsal skull surface: absent (0); present (1).
4. Prefrontal B — Prefrontal-vomer contact: absent (0); present (1).
5. Prefrontal C — Prefrontal-palatine contact: present (0); absent (1).
6. Prefrontal D — Dorsal prefrontal exposure: large (0); reduced (1); absent or near absent (2).
7. Prefrontal E — Prefrontals heavily sculptured: present (0); absent (1).
8. Lacrimal A — Lacrimal: present (0); absent (1).
9. Frontal A — Frontal contribution to orbit: absent, prefrontal-postorbital contact present (0); present (1).
10. Parietal A — Parietal-squamosal contact: present (0); absent (1).
11. Parietal B — Parietal contact with pterygoid, epipterygoid, or palatine: absent, trigeminal foramen only developed as a notch (0); present, trigeminal foramen enclosed by bone (1).
12. Parietal C — Anterior extension of lateral braincase wall: short, only a narrow strut anterior to trigeminal foramen (0); elongate, extended process anterior to trigeminal foramen (1).
13. **Parietal D** — Posteroventral process of the parietal forming most of the posterior margin of the trigeminal foramen, contacting the pterygoid, and excluding the prootic from the foramen margin: absent (0); present (1).
14. Jugal A — Jugal-squamosal contact: present (0); absent (1).
15. Jugal B — Jugal participation to upper temporal rim: absent (0); present, extensive

upper temporal emargination (1).

16. Quadratojugal A — Quadratojugal: present (0); absent (1).
17. Quadratojugal B — Quadratojugal-maxilla contact: absent (0); present (1).
18. Squamosal B — Squamosal-postorbital contact: present (0); absent, temporal roofing well developed but postorbital short (1); absent, due to lower temporal emargination (2); absent, due to upper temporal emargination (3).
19. Squamosal B — Squamosal-supraoccipital contact: absent (0); present (1).
20. Postorbital A — Postorbital-palatine contact: absent (0); present, foramen palatinum posterius situated posterior to orbital wall (1).
21. Supratemporal A — Supratemporal: present (0); absent (1).
22. Premaxilla A — Subdivision of external nares by premaxilla: present (0); absent (1).
23. Premaxilla B — Fusion of premaxillae: absent (0); present (1).
24. Premaxilla C — Foramen praepalatinum: present (0); absent, premaxillae well ossified (1); absent, foramen intermaxillaris present (2).
25. Premaxilla D — Exclusion of premaxillae from apertura narium externa: absent (0); present (1).
26. Premaxilla E — Premaxillary hook: absent (0); present (1).
27. Vomer A — Vomer: paired (0); single (1); single, greatly reduced or absent (2).
28. Vomer B — Vomer-pterygoid contact in palatal view: present (0); absent, medial contact of palatine present (1).
29. Vomer C — Vomerine and palatine teeth: present (0); absent (1).
30. Palatine A — Palatine contribution to anterior extension of braincase wall: absent (0); present, well-developed (1).
31. Quadrate A — Flooring of the cavum acustico-jugulare and recessus scalae tympani: absent (0); fully or partially present, produced by the posterior process of the pterygoid (1); fully or partially present, produced by the ventral of the quadrate or the prootic, or both (2).
32. Quadrate B — Central constriction of the middle ear: absent (0); present (1).
33. Quadrate C — Cavum tympani: absent (0); partially developed (1); fully developed (2).
34. Quadrate D — Precolumellar fossa: absent (0); large and deep (1).
35. Quadrate E — Antrum postoticum: absent (0); present, quadrate does not fully enclose the anterior perimeter of the antrum (1); present, quadrate fully encloses the anterior perimeter of the antrum (2).
36. Quadrate F — Incisura columellae auris: absent (0); present, but open

posteroventrally (1); present and closed, but only enclosing the stapes (2); present and closed, enclosing stapes and the Eustachian tube (3).

37. **Quadrate G** — Infolding ridge on the posterior surface of quadrate below the incisura columellae auris: absent (0); present (1).

38. Epipterygoid A — Epipterygoid: present (0); absent (1).

39. **Epipterygoid B** — Epipterygoid excluded from the margin of the foramen nervi trigemini by a parietal-pterygoid contact: absent (0); present (1).

40. Pterygoid A — Pterygoid teeth: present (0); absent (1).

41. Pterygoid B — Basipterygoid articulation: open (0); fused (1).

42. Pterygoid C — Interpterygoid vacuity: present (0); absent (1).

43. Pterygoid D — Pterygoid-basioccipital contact: absent (0); present (1).

44. Pterygoid E — Processus trochlearis pterygoideus: absent (0); present (1).

45. Pterygoid F — Foramen palatinum posterius: present (0); present, but open laterally (1); absent (2).

46. Pterygoid G — Medial contact of pterygoids: present (0); absent (1).

47. Pterygoid H — Pterygoid contribution to foramen palatinum posterius: present (0); absent (1).

48. Supraoccipital A — Crista supraoccipitalis: poorly developed (0); protruding significantly posterior to the foramen magnum (1).

49. Supraoccipital B — Large supraoccipital exposure to dorsal skull roof: absent (0); present (1).

50. Exoccipital A — Medial contact of exoccipitals dorsal to foramen magnum: absent (0); present (1).

51. Opisthotic A — Processus paroccipitalis: loosely articulated to squamosal and quadrate (0); tightly sutured to squamosal and quadrate (1).

52. Basisphenoid A — Rostrum basisphenoidale: flat (0); rod-like, thick, and rounded (1).

53. Basisphenoid B — Paired pits on ventral surface of basisphenoid: absent (0); present (1).

54. **Basisphenoid C** — Position of sella turcica relative to dorsum sellae: dorsum sellae overhangs sella turcica (0); dorsum does not overhang sella turcica, development of an anteriorly sloping surface below dorsum sellae (1).

55. **Basisphenoid D** — Complete ossification of pila prootica: absent (0); present (1).

56. Hyomandibular Nerve A — Path of hyomandibular branch of the facial nerve: through cranio-quadrate space parallel to vena capitis lateralis (0); independent from vena capitis lateralis (1).

57. Stapedial Artery A — Position of stapedio-temporal canal: posterior to fenestra ovalis between paroccipital process and quadrate (0); anterior to fenestra ovalis between quadrate and prootic (1).
58. Stapedial Artery B — Size of foramen stapedio-temporale: relatively large, the size of a large blood foramen (0); significantly reduced in size, the size of a nerve foramen (1); absent (2).
59. Jugular Foramina A — Anterior and posterior jugular foramina defined by bone: absent (0); present (1).
60. Canalis Caroticus A — Foramen posterius canalis carotici interni: formed by basisphenoid only (0); formed by basisphenoid and pterygoid halfway along the basisphenoid-pterygoid suture (1); formed by prootic only (2); formed mostly or fully by pterygoid (3).
61. Fenestra Perilymphatica A — Fenestra perilymphatica: large (0); reduced in size to that of a small foramen (1).
62. Dentary A — Medial contact of dentaries: fused (0); sutured only (1).
63. Splenial A — Splenial: present (0); absent (1).
64. Carapace A — Carapacial scutes: present (0); partially present (1); absent (2).
65. Carapace B — Tricarinate carapace: absent (0); present, but only poorly developed (1); present and pronounced (2).
66. Nuchal A — Articulation of nuchal with neural spine of eighth cervical vertebra: blunt facet (0); articulation absent (1); raised pedestal (2).
67. Nuchal B — Elongate costiform processes of nuchal: absent (0); present, process often contacts peripheral 2 and sometimes peripheral 3 (1).
68. Neural A — Neural formula 6>4<6<6<6<6: absent (0); present (1).
69. Peripheral A — Number of peripherals: more than 11 pairs (0); 11 pairs (1); 10 pairs (2); less than 10 pairs (3).
70. Peripheral B — Anterior peripherals incised by musk ducts: absent (0); present (1).
71. Costal A — Medial contact of costal 1: absent (0); present (1).
72. Costal B — Medial contact of posterior costals: absent (0); medial contact of up to three posterior costals (1); medial contact of all costals (2).
73. Costal C — Reduction of costal ossification: absent, costals fully or almost fully ossified, costal fontanelles small or absent (0); present, costals ossified only two thirds the length of the costal ribs, costal fontanelles well developed (2).
74. Cervical A — Cervical: one cervical present (0); cervical absent, carapacial scutes otherwise present (1); more than one cervical present (2).

75. Supramarginal A — Supramarginals: complete row present (0); partial row present (1); absent (2).
76. Vertebral A — Number of vertebrals: four present (0); five or more present (1).
77. Vertebral B — Shape of vertebrals: vertebrals 2 to 4 significantly broader than pleurals (0); vertebrals 2 to 4 as narrow or narrower than pleurals (1).
78. Vertebral C — Position of vertebral 3–4 sulcus in taxa with five vertebrals: sulcus positioned on neural 6 (0); sulcus positioned on neural 5 (1).
79. Plastron A — Connection between carapace and plastron: osseous (0); ligamentous (1).
80. Plastron B — Central plastral fontanelle: absent in adult individuals (0); present, even in adult individuals (1).
81. Plastron C — Plastral kinesis: absent (0); present (1).
82. Entoplastron A — Anterior entoplastral process: present, medial contact of epiplastra absent (0); absent, medial contact of epiplastra present (1).
83. Entoplastron B — Size of posterior entoplastral process: posterior process long (0); posterior process reduced in length (1).
84. Entoplastron C — Distinct posterolateral entoplastral processes: present (0); absent (1).
85. Entoplastron D — Entoplastron: massive and cross- to diamond-shaped (0); strap like and V-shaped (1).
86. Entoplastron E — Entoplastron: present (0); absent (1).
87. Epiplastron A — Shape and contacts of epiplastra: epiplastra squarish in shape, minor posterior contact with hyoplastra (0); epiplastra elongate in shape, long posteromedial contact with hyoplastra (1).
88. Hyoplastron A — Contacts of axillary buttresses: peripherals only (0); peripherals and first costal (1).
89. Mesoplastron A — Number and size of mesoplastra: one or two pairs of mesoplastra present that fully hinder any contact between hyo- and hypoplastra (0); one reduced pair of mesoplastra present that allows partial contact between the hyo- and hypoplastra (1); mesoplastra absent (2).
90. Hypoplastron A — Contacts of inguinal buttresses: peripherals only (0); peripherals and costal 5 (1); peripherals, costal 5, and costal 6 (2).
91. Xiphiplastron A — Distinct anal notch: absent (0); present (1).
92. Xiphiplastron B — Xiphiplastral shape: elongate rectangles (0); narrow struts that frame a xiphiplastral fontanelle (1).

93. Plastral Scutes A — Plastral scutes: present (0); absent (1).
94. Plastral Scutes B — Midline sulcus: straight (0); distinctly sinuous (1).
95. Gular A — Gulars: one, medially situated pair of scutes present (0); one, medially situated scute present (1).
96. Extragular A — Extragulars: present (0); absent (1).
97. Extragular B — Medial contact of extragulars: absent (0); present, contacting one another anterior to gular(s) (1); present, contacting one another posterior to gular(s) (2).
98. Extragular C — Anterior plastral tuberosities: present (0); absent (1).
99. Intergular A — Intergulars: absent (0); present (1).
100. Humeral A — Humerals: one pair present (0); two pairs present, subdivided by plastral hinge (1).
101. Pectoral A — Pectorals: present (0); absent (1).
102. Abdominal A — Abdominals: present, in medial contact with one another (0); present, medial contact absent (1); absent (2).
103. Anal A — Anals: only cover parts of the xiphiplastra (0); anteromedially overlap onto hypoplastra (1).
104. Inframarginal A — Inframarginal scutes: more than two pairs present (0); two pairs present, axillary and inguinal (1); absent (2).
105. Cervical Rib A — Cervical ribs: large cervical ribs present (0); cervical ribs reduced or absent (1).
106. Cervical Vertebra A — Position of transverse processes: middle of the centrum (0); anterior end of the centrum (1).
107. Cervical Vertebra B — Posterior cervicals with strongly developed ventral keels: absent (0); present (1).
108. Cervical Vertebral C — Cervical centrum 8 significantly shorter than 7: absent (0); present (1).
109. Cervical Articulation A — Cervical central articulations: articulations not formed (0); articulations formed (1).
110. Cervical Articulation B — Articulation between cervicals 2 and 3: 2(3 (0); 2)3 (1).
111. Cervical Articulation C — Articulation between cervicals 3 and 4: 3(4 (0); 3)4 (1).
112. Cervical Articulation D — Articulation between cervicals 4 and 5: 4(5 (0); 4)5 (1).
113. Cervical Articulation E — Articulation between cervicals 5 and 6: 5(6 (0); 5)6 (1).
114. Cervical Articulation F — Articulation between cervicals 6 and 7: 6(7 (0); 6)7 (1).
115. Cervical Articulation G — Articulation between cervicals 7 and 8: 7(8 (0); 7)8 (1).

116. Cervical Articulation H — Articulation between cervical 8 and the first dorsal: 8(dorsal (0); 8)dorsal (1); none, vertebrae only meet at zygapophyses (2).

117. Dorsal Rib A — Length of first dorsal rib: long, extends full length of first costal and may even contact peripherals distally (0); intermediate, in contact with well-developed anterior bridge buttresses (1); intermediate to short, extends less than halfway across first costal (2).

118. Dorsal Rib B — Contact of dorsal ribs 9 and 10 with costals: present (0); absent (1).

119. Dorsal Rib C — Dorsal rib 10: dorsal rib 10 long, spanning full length of costals and contacting peripherals distally (0); dorsal rib 10 short, not spanning farther distally than pelvis (1).

120. Dorsal Vertebra A — Anterior articulation of first dorsal centrum: faces at most slightly anteroventrally (0); faces strongly anteroventrally (1).

121. Chevron A — Chevrons: present on nearly all caudals (0); absent, or only poorly developed, along the posterior caudals (1).

122. Caudal A — Tail club: present (0); absent (1).

123. Caudal B — Caudal centra: all centra amphicoelous (0); all centra more or less pronounced procoelous (1); all centra more or less opisthocoelous (2); anterior few centra procoelous, posterior centra predominantly opisthocoelous (3).

124. Cleithrum A — Cleithra: present and in contact with the carapace (0); present, osseous contact with carapace absent (1); absent (2).

125. Scapula A — Length of acromial process: less than one half the length of scapular process (0); more than one half the length of scapular process (1).

126. Scapula B — Acromial ridge: present, contacts the glenoid proximally (0); absent (1).

127. Scapula C — Glenoid neck present on scapula: absent (0); present (1).

128. Coracoid A — Coracoid foramen: present (0); absent (1).

129. Pelvis A — Sutural articulation of pelvis to shell: absent (0); present (1).

130. Ilium A — Elongated iliac neck: absent (0); present (1).

131. Ilium B — Iliac scar: extends from costals onto peripherals and pygal (0); positioned on costals only (1).

132. Ilium C — Shape of ilium articular site: narrow and pointed posteriorly (0); oval (1).

133. Ilium D — Posterior notch in acetabulum: absent (0); present (1).

134. Ischium A — Ischial contacts with plastron: contact by way of a large central tubercle (0); contact by way of two separate ischial processes (1).

135. Hypoischium A — Hypoischium: present (0); absent (1).

136. Manus A — Phalangeal formula of manus and pes: most digits with three elongate

phalanges (0); most digits with two short phalanges (1).

137. Manus B — Paddles: absent (0); short paddles present (1); elongate paddles present (2).

138. Manus C — Flippers: absent (0); short flippers present (1); elongate flippers present (2).

139. Pes A — Claw of fifth digit: present (0); absent (1).

140. Pes B — Metatarsal 5 and "functional metatarsal 5": metatarsal 5 functions as a true metatarsal (0); metatarsal 5 functions as a tarsal, with the first phalanx of digit 5 functioning as a metatarsal (1).

II. Data matrix

The character-taxon matrix consists of 141 and 71 taxa. It was assembled on MorphoBank (O'Leary and Kaufman, 2012) and is available there as project P1166 (http://morphobank.org/permalink/?P1166). The matrix is also provided in TNT format as S2 Dataset.

```
nstates num 4;
xread
'File output by Morphobank v3.0 (http://www.morphobank.org); 2015-01-16 08.16.27'
141 71

Hypothetical_ancestor                 000000000000000000000000000000000000000?
000000000000000?
000000000000000000000000000000000000000000000000000000-------000000000000000000000000
Proganochelys_quenstedti              000000000000--000000000000000000000000??
0000000000000001000000000000?000000000-0000000000000000000000000?
00000-------00000000000000--00000000
Proterochersis_robusta                ??????????????????????????????????????????????????????
??????????00??00??0?110?00000100000010000000000000???????????????????0?????1??00??????
Palaeochersis_talampayensis           00000?01000???00000000??00001?0?10010??0100000000?1?
001?100???00????0??0?0?0?00000?00?0000000???0????0?0???0-------0????1?000?0?0??01000000
Australochelys_africanus              ???????10?0????00?0???0??00?0??0010010???10000000??1?
0???100?0???????????????????????????????????????????????????????????????????????????????
Kayentachelys_aprix                   00001011110???10000001100001010012 0110??
010000000001000??1010000000001000002100000001000000000000001000000?00?
0-------00000101100101--011?????
Meiolania_platyceps
00001001100100100000101[0,1]00001010112013000111 1000[0,1]1101000011013000000 0?100000210?
110101001 0?00000000100????00001001111100?000[2,3]1100101--01?000?0
Mongolochelys_efremovi                0010100110010?1000010110000101011201100?111000001101?
0???101000000000100000210111010100100000000000100000000001011100100000131100101--0??10011
Kallokibotion_bajazidi                0010??011?01?010000001000001010112013000 1111??0??
0010000?1010?0?0000010000?21000001?10001110000000100000 0?0??0-------0???0?01??0?0?--????????
Platychelys_oberndorferi              ??????????????????????????????????????????????????????
?????????00100100000110001011100011110001001000002?0??1???????10101???????110001??????
Caribemys_oxfordiensis                ??????????????????????????????????????????????????????
?????????00100?0??0?2???01011?000111?00010010000?2?0??1????????0??????????1?10????????
Notoemys_laticentralis                ??????????????????????????????2120??0??11?0??????01?
0???1010???0010010000021000101 1?00011?1000???1?00002?0??100????201?????????1?11???10011
Elseya_dentata
01100111110110101-201110000101021202301-11101000100100001101200000100100201211-00011100012110
0010010000021000100011012010111211111111101110011
Chelodina_siebenrocki
01100111111110101-201110000101021202301-11101000001100001101201000100100200211-00011100012010
0010110000021000100011012010111211111111101110011
Phrynops_geoffroanus
01100111110110101-201110000101021202301-1110100000110000110120100010010010021110001110001 2110
```

00100100000210001000110120101112111111101110011
Erymnochelys_madagascariensis
1--101011111101000101110000211021212301-111010001001000011010001001001001012111000111000111110
00100100000210001111111120101112110111110111001 1
Pelomedusa_subrufa
1--101011111101000301110000211021212301-11101000100100001101200100100100101211100011100011110
0010010000021000111111112010111211011111011100 11
Podocnemis_expansa
1--101011111101000101110000211021212301-11101001100100001101000100100100101211100011100011110
00100100000210001111111120101112111111101110011
Dorsetochelys_delairi 001010011101??1000000110000101?11201100?111100000001?
0??01011??
Pleurosternon_bullockii 0110??11110110100000?1100001?
1011201100011110010000100000101 11??00?0010000021010001??000?0?00001001000000?0??
0-------0???????????1??0???????
Glyptops_plicatulus 01101?1111?110100????110?011?101120?100?111100100??
1000001011?000010010000021110001110001010000000010000 00?0000-------0010??1111101--011?????
Dinochelys_whitei 0?10??1111????10000???10?01??????20?1???????????????
0????101??0?00??01000002101000111000?0?000000010000[0,1]010??0-------0?1??1?1???????????10011
Neurankylus_eximius ???0??11?1?110????001??????????1120[1,2]101-11110?0?
0001000001011 1?0001001000002111000111000101?0000001000000????????????101?????????0?--????????
Trinitichelys_hiatti 0010102111111010000000110000101011 20?
101-11110000000100??0101110000100?00?0021110001110001 01?00000010000?0?0000-------??
10???????????????????
Plesiobaena_antiqua 0010102111111010000000110000101011 20?
101-11110000000100??01011100001001000002111000111000101000000210000101000 1???11111010010?
111101--01110011
Boremys_pulchra
0010102111111010000000110000101011201101-1111000100 0100??01011100001001000022111000111000101?
00000210000?010000-------10100122????0?--????????
Baena_arenosa 1??0102111011010000000110000101011 20?
101-1111000000010000010111010010010000221110001110001110000002100001 0????????????1010???2????
0?--011?????
Chisternon_undatum
0010102111011010000000110000101011201101-11110001000100000101110100100100002211-00011100010100
000021000010?0001001111101001 2?111101--?11?????
Portlandemys_mcdowelli 0011100111?101??????0110000101011201110111110000?
001001001013000???
Plesiochelys_etalloni 0011100111?
101100000011000010101120111011111010010010011010130000010010000221010[0,1]0111000121000000010
00000????????????0010????111101--01110011
Plesiochelys_planiceps ???1100111?101??????0??00??1?101120?
1101111101001001001001013000???
????????????
MJSN_BSY009_708 0010??11?1?1?1??0??????????????112011101?111??0??
001001001013?00???
Tropidemys_langii ??
??????????00100100002211[0,1]0[0,1]0??????121??00????00000?????????????
101?????????????????????
Solnhofia_parsonsi 0011100111?1011000?
0011000011101120111001111000010010000010130000 0??01?00002101110???0000200000????00000110?

```
0-------??1??10?11110?--?1110011
_Thalassemys_moseri
00101011110101100000011000011101120111011111010000010000010130??00100?00?0?2?0??10??????12???
00?????000?011??0-------1???????????????????????
Santanachelys_gaffneyi                 0010100111110010000001l0?0011101120110??111101001001?
0??01013?0?00?00100010210011011100002000?0??????????11??????????0?1?????11?10?--01111011
Xinjiangchelys_latimarginalis          ??????????????????????????????1??????????1??????????
1??????????0010010000021111001110000200000000100001O?1000-------20100131111101--011?????
Hangaiemys_hoburensis                  001110011101l?10000001100001010112?[1,2]10??
111100101001?1??01013???0010010000021111001110010200000001-10000[0,1]0111?100111112?
10????????????????????
Judithemys_sukhanovi                   ???1??0111?11?1000?0011???0101?1120[1,2]10??
1111000010010100010l3?0?0010010000021111001110010200000001-10000[0,1]0111?100111112?10113?11?
101--01110011
Dracochelys_bicuspis                   ???11?011111101000?0?110000101?1121[1,2]10??
111100001001????01013???????????????????1????????1?????
0???????????????????????????????????????????????
Sinemys_lens                           0010??1111111?100030?11??00??1?1121220??111000001001?
10?0101300?0010010000121111[0,1]?11100-0200000???1?000?0???0????????0????1??????????????10011
Ordosemys_leios                        0010100111?11110000000110000101011211100?
111100001001010?01013???0010010000021111l????00?0200000???1?0001011?01?01??010?10113?
111101--011?????
Toxochelys_latiremis                   001110011111?0100000011000010101120110??
1111000010010010010l3?01002001000102111110111001020010O???1?0?01011?110011111?010011?
11110?--01111011
Caretta_caretta                        1--110011[0,1]010010000001101001010112O1100?
1111020-1001101001013001002000000102111110111001020010001-11000001111100111112011111211110l--
01112011
Chelonia_mydas                         1--1?0011101001000000110101101011201100?
1111020-1001101001013001002001000102111110111001020010001-1100000111110011111201111121111O?--
01112011
Mesodermochelys_undulatus              ?????????????????????????????????????????????????
10????????????0110200l0001?2???110111001020O1?0??????????11101???????20101112111101--011?????
Dermochelys_coriacea
1--111011000--000000011000010101120l101-1111020-100110100101300120200 3-0-1-2---11-11100102001
10---1------11111001111120-01112111101--01112011
Protochelydra_zangerli                 1--11?0110111?1000000110000101?1120220??111100011001?
0??010130???????????0?????100111001020000O?1-1000110????????????????????2110101--0???????
Macroclemys_temminckii                 1--1100110111010000001100011010112O2200?111100?
11001000000101300100110100000111111011100102000000l-1000110111110011111201101321l0101--0111001
1
Chelydra_serpentina
1--1100110111010000001100011010112022000111100011001000000101300100110100000211111011100102000
0001-100011011111001111120110132110101--01110011
Platysternon_megacephalum              1--1100110111010010001100011010112O1200?
11110001100100000101300100100100000211110011100002010O001-100000011111001110120110132110101--
01110011
Mongolemys_elegans                     1--[0,1]10011111101000000110000101011202100?
1111000010?100??0101300100100100000211100011100012110O001-1000000????100111012?1101?
2110101--011100??
Gopherus_polyphemus                    1--1100111111010003001100001010112O2200?
```

11110001100100000101300100100100000211100011100012110000 1-1000001111110011101201111 2110101--
01100011
Chrysemys_picta 1--11001111101000300110000101011202100?
1110000110010000010130010010010000021110001110001211000 01-1000001111110011101201111 2110101--
01110011
Geoclemys_hamiltonii 1--11001101110110030011000010101 1202100?
11110001100100000101300101100100000211100011100012110000 1-1000001111110011101201111 2110101--
01110011
Emarginachelys_cretacea 1--1100111111010003001100001011 1120[1,2]100?
1111000110010000010 13???0111010000?21111001110000200000 01-100??0011??10011111??1?????
110101--01110011
Baptemys_wyomingensis 1--11001111101000300110000101111202100?
1111000[0,1]1001?0000-213001011001000002111000111000121000001-1001000111110111 1112?
111132110101--11110011
Dermatemys_mawii 1--110011[0,1]11101000300110000101111202100?
11110001100100000-213001001[0,1]0100100211100011100012010000 1-1101000111111111111201111121101
01--01110011
Hoplochelys_crassa ??
???????0??021101000002111000111000020000001-1001100????????????201????2??010?--????????
Staurotypus_triporcatus 1--1100110111?1101300110001101111202100?
11110001100100??
0111300102110210[0,1]002111001111000020000001-1001200111110111111211111 1211010?--11110011
Sternotherus_odoratus 1--11001101110100130011000010111 1202100?
1111000110010000011130010111021110021110011 11?
10020000001-1111200111110111111211111 12110101--11110011
Kinosternon_flavescens 1--1100110111?10013001100001011 11202100?
11110001100100??01113001011102111002111001111?
10020000001-111120011111011111121111 11211010?--11110011
Zangerlia_neimongolensis 1--11?0110?1??1?003001100001?1?1120[1,2]20??
111100001001?0??01013?0100??11??00?211?0001110000200001002100000011111000000 12??1????
110101--011000?1
Basilemys_variolosa 1--1??01111???100300110?00??1??120220??111?0??????
1??????????0100101100[0,1]002111000111000020000100[0,2]1000000111110 00000????????211?
101--011000??
Adocus_beatus 1--11001111101?003001100001011 11202100?
111100011001000001013001001011001002111000111000020000100010000000111110 0000002?11???
2110101--01110011
Peltochelys_durlstonensis ??
??????????0010020010?2110000111000020000010010000 00????????????????????????????????????
Sandownia_harrisi 1--1100111011010000001 1[0,1]10011101120?20??
11110000000100100101300??
????????
Apalone_spinifera 1--111011111101100300111210111111201200?
111100111001000001013001201003-01--2---11-11110-0200-1----1------110110000002201-1112110101--
01110111
Lissemys_punctata 1--111011111101100300111210111111201200?
111100111001000001013001201003-01--2---11-11110-0200-1----1------110110000002201-1112110101--
01110111
Anosteira_ornata 1--1??01111???10003001 1?200111?1120220??
1111001[0,1]1001?0??010130??10100200100211?10-111000020001----1------????????????????????11??
0?--?1110211

```
Carettochelys_insculpta                1--11101111110100130011120011111120220 0?
11110011100100??
010130011010020010-2---10-111000020001----1------110110000001201111121101 01--01110211
;
```

III. Codings altered compared to the original matrix of Joyce (2007)

Some of the original codings of Joyce (2007) were altered in the course of the present study. Details are provided below. Most of the following modifications concern plesiochelyids and closely related taxa, or *Sandownia harrisi*, since several recent studies provided new information on this species (Mateus et al., 2009; Tong and Meylan, 2013).

Character 10 (Parietal A). This character codes for the presence/absence of a parietal-squamosal contact. *Plesiochelys etalloni* and *Solnhofia parsonsi* were initially scored as polymorphic (0,1). The condition in these taxa is not polymorphic, but uncertain. They are therefore scored as uncertain (?) in the present study.

Character 12 (Parietal C). This character code for the length of the anterior extension of the lateral braincase wall. '*Thalassemys*' *moseri* sensu Rieppel (1980) was initially scored as elongate (1). However, the anterior extension of the lateral braincase wall is rather short in this taxon and the interorbital foramina are well developed, a condition similar to that of plesiochelyids. We therefore scored this taxon as short (0) in the present study.

Character 24 (Premaxilla C). This character codes for the presence/absence of the foramen praepalatinum. *Sandownia harrisi* was initially scored as unknown (?). However, according to Tong and Meylan (2013), the foramen praepalatinum is absent in *Sandownia harrisi* and the premaxilla is well ossified. This taxon is therefore scored with state 1 in the present study.

Character 30 (Palatine A). This characters codes for the presence/absence of a palatine contribution to the anterior extension of the lateral braincase wall. *Sandownia harrisi* was initially scored as having a palatine contribution (1). However, the palatine does not truly contribute to the anterior extension of the braincase wall in *Sandownia harrisi* and closely-related taxa (Mateus et al., 2009; Tong and Meylan, 2013). We therefore scored this taxon as lacking a palatine contribution (0) in the present study.

Character 35 (Quadrate E). This multistate character codes for the absence/presence of an antrum postoticum and the contribution of the quadrate to its anterior perimeter. *Portlandemys mcdowelli* and *Sandownia harrisi* were initially scored as polymorphic (1,2). However, uncertainty was meant instead of polymorphism. Based on personal observations,

Portlandemys mcdowelli is scored as having an antrum postoticum with a quadrate that does not fully enclose its anterior perimeter (1) in the present study. *Sandownia harrisi* is scored as uncertain (?).

Character 38 (Epipterygoid A). This character codes for the presence/absence of the epipterygoid. *Sandownia harrisi* was initially scored as lacking an epipterygoid (1). However, the original publication (Meylan et al., 2000) is unclear on the matter and an epipterygoid is apparently present in closely-related taxa (Mateus et al., 2009; Tong and Meylan, 2013). Therefore, we decided to score *Sandownia harrisi* as uncertain (?) in the present analysis.

Character 45 (Pterygoid F). This character codes for the presence/absence of a foramen palatinum posterius. *Sandownia harrisi* was initially scored as having a foramen palatinum posterius that remains open laterally (1). Meylan et al. (2000) and Tong and Meylan (2013) both interpreted the foramen palatinum posterius as being fully enclosed by bone in *Sandownia harrisi*. In the present study, the foramen palatinum posterius is therefore scored as present (0) in this taxon.

Character 47 (Pterygoid H). This character codes for the presence/absence of a pterygoid contribution to the foramen palatinum posterius. *Plesiochelys etalloni*, '*Thalassemys*' *moseri* sensu Rieppel (1980), *Santanachelys gaffneyi*, and *Sandownia harrisi* were initially scored as "non-applicable" (—). In the three first taxa, the foramen palatinum posterius remains open laterally, but the pterygoid still contributes to its posterolateral margin. This is confirmed by the original literature (Gaffney, 1976; Rieppel, 1980; Hirayama, 1998), and by firsthand observations in the case of the two first taxa. *Plesiochelys etalloni*, '*Thalassemys*' *moseri* sensu Rieppel (1980), and *Santachelys gaffneyi* were therefore scored as having a pterygoid contribution to the foramen palatinum posterius (0) in the present study. The original scoring of *Sandownia harrisi* for character 47 (Pterygoid H) is linked to the interpretation of the condition of the foramen palatinum posterius (see Character 45 above) in this taxon. Based on the literature (Meylan et al., 2000; Tong and Meylan, 2013), the foramen palatinum posterius is entirely enclosed by bone with a contribution from the pterygoid in *Sandownia harrisi*. This taxon is therefore scored as having a pterygoid contribution to the foramen palatinum posterius (0) in the present study.

Character 48 (Supraoccipital A). This character codes for the development of the crista supraoccipitalis. *Portlandemys mcdowelli* was initially scored as having a crista

supraoccipitalis that significantly protrudes posterior to the foramen magnum (1). Available specimens (see main text) do not allow to reach such a conclusion, although it is probably a safe one. *Portlandemys mcdowelli* is therefore scored as uncertain (?) in the present study.

Character 63 (Splenial A). This characters codes for the presence/absence of the splenial. *Sandownia harrisi* was initially scored as lacking a splenial (1). In their original publication, Meylan et al. (2000) were unsure regarding the presence or absence of the splenial in this taxon. Indeed, the bone labelled as the angular in their figure 5 (Meylan et al. 2000) could easily be alternatively interpreted as the splenial. Furthermore, the splenial is apparently present in *Brachyopsemys tingitana*, a close relative of *Sandownia harrisi* (Tong and Meylan, 2013). In order to avoid favoring any hypothesis, *Sandownia harrisi* is scored as uncertain (?) in the present study.

Character 117 (Dorsal Rib A). This character codes for the development of the first dorsal rib. *Tropidemys langii* was initially scored as having an intermediate to short first dorsal rib extending less than halfway across the first costal (2) (Püntener et al., 2014). The condition in *Tropidemys langii* is however not truly different from that of *Plesiochelys etalloni* and '*Thalassemys*' *moseri* sensu Rieppel (1980). In the present study, *Tropidemys langii* is therefore scored as having an intermediate first dorsal rib that contacts a well-developed axillary buttress (1).

IV. Analysis with TNT

The character-taxon matrix was analysed using the Mac OS 64 bits command-driven version of TNT v. 1.1 (Goloboff et al., 2008) with all of the characters equally weighted. Branches were set to collapse if they lack unambiguous support (rule 1 of Coddington and Scharff, 1994). As in the original phylogenetic analysis (Joyce, 2007), the following 15 characters were ordered: 6 (Prefrontal A), 27 (Vomer A), 33 (Quadrate C), 35 (Quadrate E), 58 (Stapedial Artery B), 64 (Carapace A), 65 (Carapace B), 69 (Peripheral A), 72 (Costal B), 75 (Supramarginal A), 89 (Mesoplastron A), 102 (Abdominal A), 124 (Cleithrum A), 137 (Manus B), and 138 (Manus C). The hypothetical ancestor (taxon 0 in TNT) was set as outgroup. A heuristic search of 1000 replicates followed by TBR branch swapping keeping 10 trees per replicate was conducted. The best trees obtained at the end of this initial search were subjected to a final round of TBR branch swapping. Bremer support values were calculated using an iterative procedure where successively longer suboptimal trees are computed and compared to the strict consensus tree.

For the sake of reproducibility, the command lines corresponding to the above procedure are reported below.

```
# Opening files and increasing memory
mxram 100;
proc matrix.tnt;
log results.out;
hold 100000;

# Set branches to collapse if lacking unambiguous support
collapse 3;

# Define ordered characters and outgroup, and display taxon names and node numbers
ccode +6 27 33 35 58 64 65 69 72 75 89 102 124 137 138;
outgroup 0;
taxname=;
naked-;

# Initial heuristic search
```

```
mult=tbr replic 1000 hold 10;

# Final round of TBR branch swapping
bbreak=tbr;

# Discard duplicate trees
unique;

# Compute strict consensus tree and keep in memory
nelsen*;

# Display strict consensus tree, show length and list of synapomorphies (replace N by tree
number)
tplot N;
length N;
apo N; apo- N;

# Calculate CI and RI (with script STATS.RUN)
stats;

# Calculate Bremer support values
hold 1000; sub 1; bbreak=tbr;
hold 2000; sub 2; bbreak=tbr;
hold 3000; sub 3; bbreak=tbr;
hold 4000; sub 4; bbreak=tbr;
hold 5000; sub 5; bbreak=tbr;
hold 6000; sub 6; bbreak=tbr;
hold 7000; sub 7; bbreak=tbr;
hold 8000; sub 8; bbreak=tbr;
bsupport;
```

V. Strict consensus tree with listed synapomorphies

The search procedure (see above) resulted in 180 most parsimonious trees of 376 steps (CI = 0.463; RI = 0.813). The strict consensus tree (393 steps; CI = 0.443; RI = 0.797) of these 180 MPTs is presented below with a list of synapomorphies.

Strict consensus tree:

```
Strict consensus of 180 MPTs with node numbers:
     ,--0 Hypothetical_ancestor
     |    ,--1 Proganochelys_quenstedti
|--71|    |    ,--4 Australochelys_africanus
     |    |    |--3 Palaeochersis_talampayensis
     `--72|    |--2 Proterochersis_robusta
          |    |    ,--5 Kayentachelys_aprix
          |    |    |         ,--7 Mongolochelys_efremovi
          `--73|    |    ,--75---6 Meiolania_platyceps
               |    |    |         ,--8 Kallokibotion_bajazidi
               |    |    |         |    ,--9 Platychelys_oberndorferi
               |    |    |         |    |    ,--10 Caribemys_oxfordiensis
               `--74|    |         |--79|    |    ,--11 Notoemys_laticentralis
                    |    |         |    `--80|    |         ,--17 Podocnemis_expansa
                    |    |         |         `--81|    ,--86|    ,--16 Pelomedusa_subrufa
                    |    |    ,--77|              |    |    `--85---15 Erymnochelys_madagascariensis
                    |    |    |    |              `--83|    ,--12 Elseya_dentata
                    `--76|    |    |                   `--82|    ,--14 Phrynops_geoffroanus
                         |    |    |                        `--84---13 Chelodina_siebenrocki
                         |    |    |    ,--18 Dorsetochelys_delairi
                         |    |    |    |         ,--19 Pleurosternon_bullockii
                         |    |    `--87|    ,--88|    ,--21 Dinochelys_whitei
                         |    |         |    |    `--90---20 Glyptops_plicatulus
                         |    |         `--89|    ,--22 Neurankylus_eximius
                         |    |              `--91|    ,--23 Trinitichelys_hiatti
                         `--78|                   `--92|    ,--24 Plesiobaena_antiqua
                              |                        `--93|    ,--25 Boremys_pulchra
                              |                             `--94|    ,--27 Chisternon_undatum
                              |                                  `--95---26 Baena_arenosa
                              |    ,--35 Santanachelys_gaffneyi
                              |    |    ,--33 Solnhofia_parsonsi
                              |    |    |    ,--34 _Thalassemys_moseri
                              |    |--97|    |--32 Tropidemys_langii
                              |    |    |    |--31 MJSN_BSY009_708
                              |    |    `--96+--30 Plesiochelys_planiceps
                              `--98|         |--29 Plesiochelys_etalloni
                                   |         `--28 Portlandemys_mcdowelli
                                   |    ,--36 Xinjiangchelys_latimarginalis
                                   |    |     ,--37 Hangaiemys_hoburensis
                                   |    |     |           ,--38 Judithemys_sukhanovi
                                   `--99|     |     ,--101|     ,--39 Dracochelys_bicuspis
                                        |     |     |     `--103|     ,--41 Ordosemys_leios
                                        `--100|     |           `--104---40 Sinemys_lens
                                              |     |           ,--42 Toxochelys_latiremis
                                              |     |           |     ,--66 Sandownia_harrisi
                                              `--102|     ,--105|     |           ,--46 Dermochelys_coriacea
                                                    |     |     `--109|     ,--110---45 Mesodermochelys_undulatus
                                                    |     |           `--108|     ,--44 Chelonia_mydas
                                                    |     |                 `--107---43 Caretta_caretta
                                                    `--106|           ,--47 Protochelydra_zangerli
                                                          |     ,--111|     ,--49 Chelydra_serpentina
                                                          |     |     `--113---48 Macroclemys_temminckii
                                                          |     |     ,--50 Platysternon_megacephalum
                                                          `--112|     |     ,--54 Geoclemys_hamiltonii
                                                                |     |     |--53 Chrysemys_picta
                                                                |     |     |--52 Gopherus_polyphemus
                                                                `--114|     |--51 Mongolemys_elegans
                                                                      |     |     ,--55 Emarginachelys_cretacea
                                                                      |     |     |           ,--57 Dermatemys_mawii
                                                                      |     |--116|     ,--117---56 Baptemys_wyomingensis
```

```
`--115|     `--118|     ,--58 Hoplochelys_crassa
      |           `--119|     ,--59 Staurotypus_triporcatus
      |                 `--120|     ,--61 Kinosternon_flavescens
      |                       `--121---60 Sternotherus_odoratus
      |           ,--64 Adocus_beatus
      |     ,--123|     ,--63 Basilemys_variolosa
      |     |     `--122---62 Zangerlia_neimongolensis
      `--124|     ,--65 Peltochelys_durlstonensis
            |     |           ,--70 Carettochelys_insculpta
            `--125|     ,--128---69 Anosteira_ornata
                  `--127|     ,--68 Lissemys_punctata
                        `--126---67 Apalone_spinifera
```

List of synapomorphies:

```
Node 72 :
      No synapomorphies
   Node 73 :
      Char. 7: 0 --> 1
      Char. 29: 0 --> 1
      Char. 33: 0 --> 1
      Char. 36: 0 --> 1
      Char. 41: 0 --> 1
      Char. 51: 0 --> 1
      Char. 57: 0 --> 1
      Char. 76: 0 --> 1
      Char. 84: 0 --> 1
      Char. 122: 0 --> 1
   Node 74 :
      Char. 4: 0 --> 1
      Char. 8: 0 --> 1
      Char. 21: 0 --> 1
      Char. 22: 0 --> 1
      Char. 27: 0 --> 1
      Char. 32: 0 --> 1
      Char. 33: 1 --> 2
      Char. 35: 0 --> 1
      Char. 59: 0 --> 1
      Char. 69: 0 --> 1
      Char. 75: 01 --> 2
      Char. 98: 0 --> 1
      Char. 124: 0 --> 1
      Char. 125: 0 --> 1
      Char. 128: 0 --> 1
      Char. 135: 0 --> 1
   Node 75 :
      Char. 19: 0 --> 1
      Char. 49: 0 --> 1
      Char. 87: 0 --> 1
      Char. 109: 0 --> 1
      Char. 123: 0 --> 3
   Node 76 :
      Char. 31: 0 --> 1
      Char. 40: 0 --> 1
      Char. 42: 0 --> 1
      Char. 48: 0 --> 1
```

```
   Char. 82: 0 --> 1
Node 77 :
   Char. 12: 0 --> 1
   Char. 88: 0 --> 1
   Char. 90: 0 --> 1
Node 78 :
   Char. 66: 0 --> 1
   Char. 83: 0 --> 1
   Char. 89: 0 --> 1
   Char. 105: 0 --> 1
   Char. 119: 0 --> 1
   Char. 126: 0 --> 1
   Char. 127: 0 --> 1
Node 79 :
   Char. 91: 0 --> 1
   Char. 95: 0 --> 1
   Char. 104: 0 --> 2
   Char. 109: 0 --> 1
   Char. 121: 0 --> 1
   Char. 129: 0 --> 1
Node 80 :
   Char. 131: 0 --> 1
Node 81 :
   Char. 132: 0 --> 1
Node 82 :
   Char. 6: 0 --> 1
   Char. 16: 0 --> 1
   Char. 60: 0 --> 2
   Char. 89: 1 --> 2
   Char. 112: 1 --> 0
   Char. 115: 1 --> 0
Node 83 :
   Char. 72: 0 --> 1
   Char. 77: 0 --> 1
   Char. 80: 1 --> 0
Node 84 :
   Char. 48: 1 --> 0
   Char. 50: 0 --> 1
   Char. 62: 0 --> 1
Node 85 :
   Char. 127: 1 --> 0
Node 86 :
   Char. 0: 0 --> 1
   Char. 3: 0 --> 1
   Char. 10: 0 --> 1
   Char. 27: 1 --> 2
   Char. 28: 0 --> 1
   Char. 34: 0 --> 1
   Char. 63: 0 --> 1
   Char. 110: 0 --> 1
   Char. 111: 0 --> 1
```

```
Node 87 :
   Char. 48: 1 --> 0
   Char. 60: 0 --> 1
Node 88 :
   Char. 1: 0 --> 1
   Char. 46: 0 --> 1
Node 89 :
   Char. 6: 0 --> 1
Node 90 :
   Char. 26: 0 --> 1
Node 91 :
   Char. 38: 0 --> 1
   Char. 77: 0 --> 1
   Char. 117: 0 --> 1
Node 92 :
   Char. 6: 1 --> 2
Node 93 :
   Char. 97: 0 --> 2
Node 94 :
   Char. 74: 0 --> 2
   Char. 123: 0 --> 2
Node 95 :
   Char. 10: 1 --> 0
   Char. 63: 0 --> 1
Node 96 :
   Char. 39: 0 --> 1
   Char. 45: 0 --> 1
   Char. 54: 0 --> 1
   Char. 74: 0 --> 2
   Char. 79: 1 --> 0
   Char. 88: 0 --> 1
   Char. 90: 0 --> 1
Node 97 :
   Char. 13: 0 --> 1
   Char. 37: 0 --> 1
Node 98 :
   Char. 60: 0 --> 3
   Char. 89: 1 --> 2
   Char. 106: 0 --> 1
Node 99 :
   Char. 53: 0 --> 1
   Char. 77: 0 --> 1
   Char. 103: 0 --> 1
   Char. 117: 0 --> 2
   Char. 123: 0 --> 3
Node 100 :
   Char. 87: 0 --> 1
   Char. 96: 0 --> 1
   Char. 107: 0 --> 1
   Char. 109: 0 --> 1
Node 101 :
```

```
   Char. 121: 0 --> 1
Node 102 :
   Char. 10: 0 --> 1
Node 103 :
   Char. 34: 0 --> 1
Node 104 :
   Char. 3: 1 --> 0
Node 105 :
   Char. 54: 0 --> 1
   Char. 66: 1 --> 2
   Char. 73: 0 --> 1
   Char. 80: 0 --> 1
   Char. 92: 0 --> 1
   Char. 123: 3 --> 1
   Char. 137: 0 --> 1
Node 106 :
   Char. 53: 1 --> 0
   Char. 108: 0 --> 1
Node 107 :
   Char. 120: 0 --> 1
Node 108 :
   Char. 45: 0 --> 2
   Char. 52: 0 --> 1
Node 109 :
   Char. 10: 1 --> 0
Node 110 :
   Char. 64: 0 --> 1
Node 111 :
   Char. 102: 0 --> 1
Node 112 :
   Char. 47: 0 --> 1
   Char. 120: 0 --> 1
   Char. 127: 1 --> 0
Node 113 :
   Char. 26: 0 --> 1
   Char. 80: 0 --> 1
Node 114 :
   Char. 87: 1 --> 0
   Char. 91: 0 --> 1
   Char. 103: 1 --> 0
   Char. 115: 1 --> 0
Node 115 :
   Char. 18: 0 --> 3
   Char. 79: 1 --> 0
   Char. 88: 0 --> 1
   Char. 90: 0 --> 1
   Char. 121: 0 --> 1
   Char. 123: 3 --> 1
Node 116 :
   Char. 30: 0 --> 1
   Char. 65: 0 --> 1
```

```
   Char. 90: 1 --> 0
   Char. 91: 1 --> 0
   Char. 115: 0 --> 1
Node 117 :
   Char. 58: 1 --> 2
Node 118 :
   Char. 58: 0 --> 1
   Char. 111: 0 --> 1
Node 119 :
   Char. 102: 0 --> 1
Node 120 :
   Char. 69: 1 --> 2
   Char. 70: 0 --> 1
   Char. 81: 0 --> 1
   Char. 102: 1 --> 2
   Char. 118: 0 --> 1
Node 121 :
   Char. 71: 0 --> 1
   Char. 86: 0 --> 1
   Char. 99: 0 --> 1
   Char. 100: 0 --> 1
Node 122 :
   Char. 136: 1 --> 0
Node 123 :
   Char. 68: 0 --> 1
   Char. 94: 0 --> 1
Node 124 :
   Char. 30: 0 --> 1
   Char. 88: 1 --> 0
   Char. 90: 1 --> 0
   Char. 91: 1 --> 0
   Char. 96: 1 --> 0
   Char. 112: 1 --> 0
   Char. 113: 1 --> 0
   Char. 114: 1 --> 0
Node 125 :
   Char. 69: 1 --> 2
Node 126 :
   Char. 15: 0 --> 1
   Char. 25: 0 --> 1
   Char. 35: 2 --> 1
   Char. 64: 1 --> 2
   Char. 69: 2 --> 3
   Char. 80: 0 --> 1
   Char. 85: 0 --> 1
   Char. 116: 1 --> 2
Node 127 :
   Char. 64: 0 --> 1
   Char. 79: 0 --> 1
   Char. 93: 0 --> 1
Node 128 :
```

```
Char. 138: 1 --> 2
```

VI. Bremer supports

Bremer support values were calculated using the iterative procedure detailed above (see IV. Analysis with TNT). They are reported on the strict consensus tree below.

Bremer support values:

```
Bremer supports (from 8000 trees, cut 0)
   ,-- Hypothetical_ancestor
   |    ,-- Proganochelys_quenstedti
|--|    |   ,-- Australochelys_africanus
   |    |   |-- Palaeochersis_talampayensis
   `--8?|   |-- Proterochersis_robusta
        |   |   ,-- Kayentachelys_aprix
        |   |   |       ,-- Mongolochelys_efremovi
        `--3|   |   ,--3--- Meiolania_platyceps
            |   |   |       ,-- Kallokibotion_bajazidi
            |   |   |       |   ,-- Platychelys_oberndorferi
            |   |   |       |   |   ,-- Caribemys_oxfordiensis
            `--4|   |       |--3|   |   ,-- Notoemys_laticentralis
                |   |       |   `--1|   |       ,-- Podocnemis_expansa
                |   |       |       `--1|   ,--2|   ,-- Pelomedusa_subrufa
                |   |   ,--1|           |   |   `--1--- Erymnochelys_madagascariensis
                |   |   |   |           `--1|   ,-- Elseya_dentata
                `--5|   |   |               `--2|   ,-- Phrynops_geoffroanus
                    |   |   |                   `--2--- Chelodina_siebenrocki
                    |   |   |   ,-- Dorsetochelys_delairi
                    |   |   |   |       ,-- Pleurosternon_bullockii
                    |   |   `--1|   ,--1|   ,-- Dinochelys_whitei
                    |   |       |   |   `--1--- Glyptops_plicatulus
                    |   |       `--1|   ,-- Neurankylus_eximius
                    |   |           `--1|   ,-- Trinitichelys_hiatti
                    `--1|               `--1|   ,-- Plesiobaena_antiqua
                        |                   `--1|   ,-- Boremys_pulchra
                        |                       `--1|   ,-- Chisternon_undatum
                        |                           `--1--- Baena_arenosa
                        |   ,-- Santanachelys_gaffneyi
                        |   |   ,-- Solnhofia_parsonsi
                        |   |   |   ,-- _Thalassemys_moseri
                        |   |--1|   |-- Tropidemys_langii
                        |   |   |   |-- MJSN_BSY009_708
                        |   |   `--1+-- Plesiochelys_planiceps
                        `--1|       |-- Plesiochelys_etalloni
                            |       `-- Portlandemys_mcdowelli
                            |   ,-- Xinjiangchelys_latimarginalis
                            |   |   ,-- Hangaiemys_hoburensis
                            |   |   |       ,-- Judithemys_sukhanovi
                            `--1|   |   ,--1|   ,-- Dracochelys_bicuspis
                                |   |   |   `--1|   ,-- Ordosemys_leios
                                `--1|   |       `--1--- Sinemys_lens
                                    |   |       ,-- Toxochelys_latiremis
                                    |   |       |   ,-- Sandownia_harrisi
                                    `--1|   ,--1|   |       ,-- Dermochelys_coriacea
                                        |   |   `--1|   ,--1--- Mesodermochelys_undulatus
                                        |   |       `--1|   ,-- Chelonia_mydas
                                        |   |           `--1--- Caretta_caretta
                                        `--1|       ,-- Protochelydra_zangerli
                                            |   ,--1|   ,-- Chelydra_serpentina
                                            |   |   `--2--- Macroclemys_temminckii
                                            |   |   ,-- Platysternon_megacephalum
                                            `--1|   |   ,-- Geoclemys_hamiltonii
                                                |   |   |-- Chrysemys_picta
                                                |   |   |-- Gopherus_polyphemus
                                                `--1|   |-- Mongolemys_elegans
                                                    |   |   ,-- Emarginachelys_cretacea
                                                    |   |   |       ,-- Dermatemys_mawii
                                                    |   |--1|   ,--1--- Baptemys_wyomingensis
                                                    `--1|   `--1|   ,-- Hoplochelys_crassa
                                                        |       `--1|   ,-- Staurotypus_triporcatus
```

```
|           `--4|   ,-- Kinosternon_flavescens
|               `--4--- Sternotherus_odoratus
|       ,-- Adocus_beatus
|   ,--1|   ,-- Basilemys_variolosa
|   |   `--1--- Zangerlia_neimongolensis
`--2|   ,-- Peltochelys_durlstonensis
    |   |       ,-- Carettochelys_insculpta
    `--1|   ,--1--- Anosteira_ornata
        `--3|   ,-- Lissemys_punctata
            `--7--- Apalone_spinifera
```

VII. Literature cited

Coddington J, Scharff N. Problems with zero-length branches. Cladistics. 1994;10: 415–423.

Gaffney ES. Cranial morphology of the European Jurassic turtles *Portlandemys* and *Plesiochelys*. Bull Am Mus Nat Hist. 1976;157: 487–544.

Goloboff PA, Farris JS, Nixon KC. TNT, a free program for phylogenetic analysis. Cladistics. 2008;24: 774–786.

Hirayama R. Oldest known sea turtle. Nature. 1998;392: 705–708.

Joyce WG. Phylogenetic relationships of Mesozoic turtles. Bull Peabody Mus Nat Hist. 2007;48: 3–102.

Mateus O, Jacobs L, Polcyn M, Schulp AS, Vineyard D, Buta Neto A, et al. The oldest African eucryptodiran turtle from the Cretaceous of Angola. Acta Palaeontol Pol. 2009;54: 581–588.

Meylan PA, Moody RTJ, Walker CA, Chapman SD. *Sandownia harrisi*, a highly derived trionychoid turtle (Testudines: Cryptodira) from the Early Cretaceous of the Isle of Wight, England. J Vertebr Paleontol. 2000;20: 522–532.

O'Leary MA, Kaufman SG. MorphoBank 3.0: Web application for morphological phylogenetics and taxonomy. 2012. Available: http://www.morphobank.org

Püntener C, Billon-Bruyat J-P, Bocat L, Berger J-P, Joyce WG. Taxonomy and phylogeny of the turtle *Tropidemys langii* Rütimeyer, 1873 based on new specimens from the Kimmeridgian of the Swiss Jura Mountains. J Vertebr Paleontol. 2014;34: 353–374.

Rieppel O. The skull of the Upper Jurassic cryptodire turtle *Thalassemys*, with a reconsideration of the chelonian braincase. Palaeontogr Abt A. 1980;171: 105–140.

Tong H, Meylan P. Morphology and Relationships of *Brachyopsemys tingitana* gen. et sp. nov. from the Early Paleocene of Morocco and Recognition of the New Eucryptodiran Turtle Family: Sandownidae. In: Brinkman DB, Holroyd PA, Gardner JD, editors. Morphology and Evolution of Turtles. Dordrecht: Springer Netherlands; 2013. pp. 187–212.
