# Supplementary material for: Portlandemys gracilis n. sp., a New Coastal Marine Turtle from the Late Jurassic of Porrentruy (Switzerland) and a Reconsideration of Plesiochelyid Cranial Anatomy
Source: PLoS One. 2015 Jun 24;10(6):e0129193. doi: 10.1371/journal.pone.0129193 (PMC4480851; doi:10.1371/journal.pone.0129193)
Supplement: Supplementary file 2 [file pone.0129193.s003.pdf]

NMS 40870
